# In Search of Effective Treatments Targeting α-Synuclein Toxicity in Synucleinopathies: Pros and Cons

**DOI:** 10.3389/fcell.2020.559791

**Published:** 2020-09-04

**Authors:** Maria Fouka, Panagiota Mavroeidi, Grigoria Tsaka, Maria Xilouri

**Affiliations:** Center of Clinical Research, Experimental Surgery and Translational Research, Biomedical Research Foundation of the Academy of Athens, Athens, Greece

**Keywords:** α-synuclein, autophagy, immunotherapy, propagation, protein aggregation, proteasome, synucleinopathies, therapeutics

## Abstract

Parkinson’s disease (PD), multiple system atrophy (MSA) and Dementia with Lewy bodies (DLB) represent pathologically similar, progressive neurodegenerative disorders characterized by the pathological aggregation of the neuronal protein α-synuclein. PD and DLB are characterized by the abnormal accumulation and aggregation of α-synuclein in proteinaceous inclusions within neurons named Lewy bodies (LBs) and Lewy neurites (LNs), whereas in MSA α-synuclein inclusions are mainly detected within oligodendrocytes named glial cytoplasmic inclusions (GCIs). The presence of pathologically aggregated α-synuclein along with components of the protein degradation machinery, such as ubiquitin and p62, in LBs and GCIs is considered to underlie the pathogenic cascade that eventually leads to the severe neurodegeneration and neuroinflammation that characterizes these diseases. Importantly, α-synuclein is proposed to undergo pathogenic misfolding and oligomerization into higher-order structures, revealing self-templating conformations, and to exert the ability of “*prion-like*” spreading between cells. Therefore, the manner in which the protein is produced, is modified within neural cells and is degraded, represents a major focus of current research efforts in the field. Given that α-synuclein protein load is critical to disease pathogenesis, the identification of means to limit intracellular protein burden and halt α-synuclein propagation represents an obvious therapeutic approach in synucleinopathies. However, up to date the development of effective therapeutic strategies to prevent degeneration in synucleinopathies is limited, due to the lack of knowledge regarding the precise mechanisms underlying the observed pathology. This review critically summarizes the recent developed strategies to counteract α-synuclein toxicity, including those aimed to increase protein degradation, to prevent protein aggregation and cell-to-cell propagation, or to engage antibodies against α-synuclein and discuss open questions and unknowns for future therapeutic approaches.

## Introduction

α-synuclein is a neuronal presynaptic protein, which physiologically regulates neurotransmitter release, whereas its pathological accumulation is the key histopathological hallmark of certain neurodegenerative disorders with similar clinical phenotypes, designated as synucleinopathies (Spillantini and Goedert, 2000). Specifically, in Parkinson’s disease (PD) and dementia with Lewy bodies (DLB), α-synuclein mostly accumulates in Lewy bodies (LBs) and Lewy neurites (LNs) in neurons (Spillantini et al., 1997, 1998), whereas in multiple system atrophy (MSA) α-synuclein deposits mostly within the cytoplasm of oligodendrocytes forming glial cytoplasmic inclusions (GCIs) (Wakabayashi et al., 1998a,b; Nakamura et al., 2015). It is widely accepted that PD, DLB and MSA pathogenesis is the result of complex molecular events and that common pathogenic mechanisms may lead to α-synuclein deposition in these disorders. However, the diversity of α-synuclein pathology observed in α-synucleinopathies is attributed to various events, such as the presence of more than one α-synuclein strain, the intracellular milieu, the interaction of α-synuclein with multiple molecular partners, and the propagation of α-synuclein within different brain regions (Peng et al., 2018b; Longhena et al., 2019). The precise genetic and/or environmental trigger for α-synuclein misfolding still remains unknown; however, genetic mutations, mitochondrial dysfunction, proteolytic systems failure and neuroinflammation have been proposed to facilitate α-synuclein spread in the diseased brain (Polymeropoulos et al., 1997; Bose and Beal, 2016; Rocha et al., 2018). Under physiological conditions, neuronal α-synuclein is found either in the cytosol in a soluble and natively unfolded monomeric or tetrameric form (Weinreb et al., 1996; Bartels et al., 2011; Wang et al., 2011; Fauvet et al., 2012) or in a membrane-bound or vesicle-associated state (Pirc and Ulrih, 2015; Gustafsson et al., 2018; Lautenschlager et al., 2018). On the other hand, pathological α-synuclein in oligomeric, pre-fibrillar and fibrillar form can spread within the diseased brain via various cell-to-cell transmission mechanisms, which are responsible either for its release from neurons or its uptake by neighboring cells. The tendency of α-synuclein to form aggregates lies in the core of its neurotoxic potential and strategies seeking to alleviate the total protein load represent an obvious therapeutic approach.

Current therapeutic approaches for PD and related synucleinopathies can provide only palliative treatment, aiming to control the motor symptoms and delay disease progression. The lack of reliable *in vivo* markers and appropriate animal models to recapitulate the symptoms of these diseases challenge therapy development; however numerous studies have suggested various therapeutic efforts to counteract α-synuclein-related pathology. In the current review, we summarize advances in understanding the pivotal role of α-synuclein in the pathogenesis of synucleinopathies and critically discuss the potential of current therapeutic approaches favoring pathology amelioration with the pros and cons of each strategy.

## The Structure, Function and Aggregation of α-Synuclein

The synuclein protein was originally identified through several and independent lines of investigation. In 1985, a neuron-specific protein of 143 amino acids (aa) was identified in Torpedo californica cholinergic synaptic vesicles (Maroteaux et al., 1988). Later studies in amyloid plaques from an Alzheimer’s disease (AD) brain discovered two unknown peptides, in addition to the major amyloid beta fragment, which were named NAC (non-A beta component of AD amyloid) peptide and its precursor, NACP (Ueda et al., 1993) and identified two proteins of 140 and 134 aa, which were highly expressed in the human brain (Jakes et al., 1994). These results revealed the existence of a new protein family expressed predominantly in presynaptic nerve terminals. The 140 aa protein was named α-synuclein, while the 134 aa protein β-synuclein (Jakes et al., 1994). The third and last protein of the family, γ-synuclein, was found to be highly expressed in ovarian and breast carcinomas (Ji et al., 1997; Bruening et al., 2000).

**FIGURE 1:**
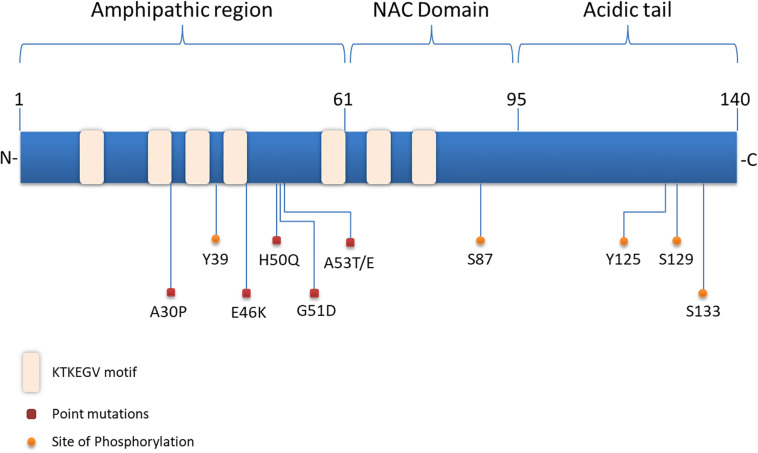
Structure of α-synuclein. The N-terminal domain of α-synuclein is characterized by the presence of repeated lipid-binding sequences and contains the mutation sites linked with familial PD. The central NAC domain is mainly hydrophobic and favors the aggregation process of the protein. The C-terminal acidic tail carries the majority of α-synuclein phosphorylation sites.

Structurally, α-synuclein encoded by the *SNCA* gene, lacks a single stable 3D structure in aqueous solutions, transmembrane domain or lipid anchor, concluding that it may behave as a peripheral membrane protein (Weinreb et al., 1996). α-synuclein is composed of three distinct domains namely N-terminal lipid-binding domain, amyloid-binding central region (NAC) and C-terminal binding domain (Figure 1). The N-terminal domain is a positively charged lysine-rich region characterized by the presence of a series of seven imperfect amphipathic 11 aa repeats containing a highly conserved KTKEGV hexameric motif, which enable the protein to acquire alpha-helical structure, thus reducing the tendency to form ß-structure and modulating the interactions with membranes (Chandra et al., 2003; Ulmer et al., 2005; Sode et al., 2006). The central NAC region is composed of nonpolar side-chains and assembles cross b-structures, which are involved in fibril formation and aggregation. Based on that, it has been proven that the deletion of specific residues (74–84) within the core region can abolish α-synuclein aggregation (Giasson et al., 2001; Rodriguez et al., 2015). Lastly, the C-terminal domain is a highly acidic tail reported to interact with metals, small molecules, proteins and other α-synuclein domains (Kim et al., 2002; Ly and Julian, 2008).

Even though α-synuclein is considered a natively unfolded, intrinsically disordered amyloid protein, it can adopt an a-helical conformation in the presence of membranes enriched with acidic phospholipid headgroups and high curvature (Davidson et al., 1998; Pirc and Ulrih, 2015) and form fibrillar assembles by converting soluble monomers into β-sheet-like secondary structures. The existence of the protein above a crucial concentration, along with its thermodynamically unstable innate behavior, favors the aggregation and accumulation process, which is closely related to its neurotoxic potential (Ferreon and Deniz, 2007; Afitska et al., 2019). Up to date the native state of α-synuclein remains controversial. Although some studies have reported that α-synuclein purified from human cells is a helically folded dynamic tetramer (Bartels et al., 2011; Wang et al., 2011; Gould et al., 2014) that resists aggregation (Bartels et al., 2011), other studies suggested that α-synuclein exists predominantly as an unfolded monomer (Fauvet et al., 2012). Interestingly, it was suggested that the PD-linked mutations A53T and E46K shift native tetramers to monomers and this underlies the disease initiation (Dettmer et al., 2015). Nonetheless, it is widely accepted that α-synuclein in the cellular milieu exists in various conformations and oligomeric states in a dynamic equilibrium, which can be affected by factors that alter the aggregation process (Cremades et al., 2012). Which particular species of α-synuclein are toxic has been debated, since some consider the amyloid-like insoluble fibrils as the mediators of α-synuclein-induced toxicity (Conway et al., 1998), whereas others suggest that oligomers or protofibrils are the toxic ones (Danzer et al., 2007; Karpinar et al., 2009; Winner et al., 2011).

Slight alterations in the physicochemical features of α-synuclein via post-translational modifications (PTMs), truncations and solution condition modifications could favor the aggregation process (Uversky et al., 2001). Interestingly, the majority of PTMs occur in the acidic tail of the protein (Figure 1) and the serine (Ser) and tyrosine phosphorylation are the most extensively studied ones. Experiments utilizing ESI-MS (electrospray ionization mass spectrometry) technology revealed several modifications of α-synuclein in PD brains, such as N- or C-terminal truncations and phosphorylation at Ser129 (Kellie et al., 2014). In healthy brain, only a small proportion of α-synuclein is phosphorylated (Gonzalez et al., 2019), whereas under pathological conditions phosphorylated α-synuclein -mostly at Ser129- is increased in the majority of pathological inclusions, including LBs in PD and DLB, GCIs in MSA and LB-type inclusions of AD (Fujiwara et al., 2002; Saito et al., 2003; Nishie et al., 2004; Waxman et al., 2009). Even though initially phosphorylation of α-synuclein was considered to act prophylactically to protein aggregation, it is now widely accepted that it precedes fibril formation (Shahpasandzadeh et al., 2014; Tenreiro et al., 2014; Oueslati, 2016). Another common phosphorylation site is at Ser87, which is also found to be increased in synucleinopathies (Paleologou et al., 2010). Tyrosine phosphorylation of α-synuclein (Y39) seems to have both prophylactic and harmful effects (Mahul-Mellier et al., 2014; Brahmachari et al., 2016) and phosphorylation at Y125 and Y133 has been suggested to be protective against α-synuclein toxicity (Kleinknecht et al., 2016; El Turk et al., 2018). Beyond phosphorylation, other posttranslational modifications have been shown to affect the aggregation process of α-synuclein, such as nitration, oxidation, acetylation and SUMOylation, but possibly with a protective manner (Krumova et al., 2011; Dikiy and Eliezer, 2014; Vinueza-Gavilanes et al., 2020). Regarding truncation, it is known that truncated forms of α-synuclein account for 10–30% of total protein in patient-derived LB inclusions. In comparison with the full-length protein, C-truncated α-synuclein forms fibrils more rapidly, with distinct coil structures than the linear fibrils formed by the full-length protein. Additionally, α-synuclein can be oxidized through interaction with dopamine, generating dopamine-modified α-synuclein adducts, leading to a decrease in fibril formation and a subsequent increase in protofibril accumulation (Conway et al., 2001), which may enhance toxicity (Norris et al., 2003). Up to date, the physiological or pathological significance of α-synuclein cleavage remains unclear.

The physiological role of α-synuclein is still poorly understood and only its contribution to certain cellular functions is known so far. The presence of α-synuclein in the presynaptic terminals denotes a potential role of the protein in synaptic function and vesicle trafficking (Jensen et al., 1998). The ability of α-synuclein to promote, as well as to sense, membrane curvature immediately suggested a possible relationship of the protein with the synaptic vesicle cycle. Overexpression of human α-synuclein was found to induce loss of synaptic vesicles and expansion of the plasma membrane through inhibition of both slow and fast vesicle endocytosis (Busch et al., 2014; Xu et al., 2016) and to promote dilation of the fusion pore, thereby accelerating the discharge of cargo (Logan et al., 2017). Importantly, α-synuclein interacts with the soluble N-ethylmaleimide-sensitive factor attachment protein receptor (SNARE) complex, while the loss or overexpression of the protein causes reduction or redistribution of the complex, respectively (Burre et al., 2010, 2014; Garcia-Reitbock et al., 2010) α-synuclein was also reported to promote vesicle clustering through its interaction with vesicle associated membrane protein 2 (VAMP2)/ synaptobrevin-2 and phospholipids (Diao et al., 2013).

Through its predominant role in vesicle trafficking α-synuclein can affect neurotransmitter release and particularly dopamine neurotransmission (Butler et al., 2017), via regulation of dopamine biosynthesis and trafficking of dopamine transporter (DAT), as well as, via modulation of DAT-mediated dopamine efflux (Butler et al., 2017). More specifically, α-synuclein was found to down-regulate indirectly the activity of tyrosine hydroxylase (TH) via inhibition of PP2A phosphatase, thus modulating dopamine production (Peng et al., 2005). However, the main effect of α-synuclein involves the regulation of DAT, where overexpressed α-synuclein enhances its interaction with DAT, altering the ionic conductance of the transporter and influencing the action potential-independent dopamine release, thus resulting in overall decrease of dopamine uptake (Swant et al., 2011; Butler et al., 2015). α-Synuclein also develops physical and functional interactions with other monoamine transporters (MATs), such as NET and SERT. In both cases, α-synuclein has been shown to negatively modulate the cell-surface expression and uptake activity of the transporters in a NAC-domain dependent manner (Wersinger et al., 2006a,b; Jeannotte and Sidhu, 2007). This finding indicates that α-synuclein may exert a homeostatic role, thus supporting that normal MAT expression may depend upon a certain baseline level of α-synuclein–MAT interaction. However, the mechanism by which α-synuclein expression alters MAT distribution should be further investigated. An opposite reported effect of α-synuclein on the dopaminergic pathway is the suppression of apoptosis of dopaminergic neurons (Jin et al., 2011). Supportive of a protective role of α-synuclein at the synapse are also findings showing a cooperative function of the protein with the synaptic vesicle protein cysteine-string protein-alpha (CSPalpha) and SNARE proteins, resulting in protection of nerve terminals against injury (Chandra et al., 2005).

Furthermore, α-synuclein can also act as a molecular chaperone, due to its high homology and interaction with the 14-3-3 proteins and their ligands, such as molecules of the Ras signaling pathway (Xu et al., 2013), thus possibly contributing to neuronal differentiation (Fu et al., 2000). Moreover, α-synuclein is part of a chaperone complex containing the Hsc70/Hsp70 chaperones, participating in the efficient neurotransmitter release (Witt, 2013). The chaperone activity of α-synuclein operates via its N-terminal interaction with the substrate protein, whereas the C-terminal region is responsible for the solubilization of the chaperone complex (Park et al., 2002).

## α-Synuclein as the Primary Culprit for Synucleinopathies

Substantial genetic, neuropathological and biochemical evidence implicates α-synuclein in the pathogenesis of PD and related synucleinopathies. Copy number variations, such as duplication or triplications of the *SNCA* gene encoding for α-synuclein, as well as point mutations and single nucleotide polymorphisms (SNPs) cause PD and DLB or increase the risk of developing the disease (Singleton et al., 2003; Chartier-Harlin et al., 2004; Zarranz et al., 2004; Nalls et al., 2014; Orme et al., 2018). Up to date six missense mutations in the *SNCA* gene are associated with autosomal dominant PD (Figure 1): Ala53Thr (A53T), Ala30Pro (A30P), Glu46Lys (E46K), His50Gln (H50Q), Gly51Asp (G51D), and Ala53Glu4 (A53E) (Polymeropoulos et al., 1997; Kruger et al., 1998; Zarranz et al., 2004; Kiely et al., 2013; Proukakis et al., 2013; Pasanen et al., 2014). The PD-linked mutations identified so far are located in the N-terminus of α-synuclein further underscoring the contribution of this region to protein aggregation. Most of them are described to cause early onset PD with rapid disease progression and additional clinical features, such as hallucinations, dementia, pyramidal tract impairment, and autonomic failure. Both mutant A53T and A30P α-synuclein mutations are disordered in dilute solution (like the wild-type protein). However, at higher concentrations, LB-like fibrils and discrete spherical assemblies are formed most rapidly by the A53T mutant (Conway et al., 1998). The A53T mutation has a moderate effect in a small region around the site of mutation, resulting in a local structural tendency for oligomerization (Conway et al., 2000; Bussell and Eliezer, 2001). On the other hand, the A30P mutation is associated with reduced formation of LB inclusions and it seems to promote formation of oligomers, rather than fibrils (Conway et al., 2000; Lazaro et al., 2014). The E46K mutation, through its conformational changes in the monomeric protein enhances the contacts between N- and C-terminus of the protein and promotes fibrillization with an increased tendency to inclusion formation (Fredenburg et al., 2007; Rospigliosi et al., 2009; Lazaro et al., 2014). The H50Q point mutation was discovered at the same year with the G51D and was linked with a late-onset phenotype of PD. H50Q was directly associated with increased α-synuclein aggregation and toxicity (Khalaf et al., 2014). Some of the families harboring these rare mutations have clinical manifestations or neuropathological features of both PD and MSA (Fanciulli and Wenning, 2015; Kiely et al., 2015). In particular, the A53T, A53E and G51D mutations, as well as the *SNCA* gene triplications are associated with a more aggressive MSA-like clinical and pathological phenotype (Kiely et al., 2015).

The genetic link between mutations and copy number variations of the *SNCA* locus and MSA is still controversial, given that common variation in the *SNCA* gene was first identified as a risk factor for MSA in 2009 (Scholz et al., 2009), but this association was not confirmed in subsequent genome-wide association studies (GWAS) (Chen et al., 2015; Sailer et al., 2016). It is interesting to note that the G51D and A53E α-synuclein mutations seem to play an essential role in the inclusion formation in both neuronal and oligodendroglial cells (Pasanen et al., 2014; Whittaker et al., 2017). During the last decade, GWAS of risk in idiopathic PD revealed that *SNCA* is the major contributor (Satake et al., 2009). However, variability in *LRRK2* (leucine-rich repeat kinase 2), *GAK* (cyclin G-associated kinase) and *MAPT* (microtubule-associated protein tau) has been also implicated, with variants in *SNCA* and *MAPT* found to represent also risk factors for MSA (Scholz et al., 2009; Simon-Sanchez et al., 2009; Vilarino-Guell et al., 2011). Overall, it is apparent that all point mutations of α-synuclein alter its secondary structure, indicating that a single mutation in the *SNCA* gene is adequate for the development of a PD-like phenotype.

α-synuclein is the main component of the proteinaceous inclusions that represent the main histopathological hallmark of synucleinopathies, designated as LBs and LNs in PD and DLB (Spillantini et al., 1997, 1998) and GCIs in MSA (Wakabayashi et al., 1998a,b). Moreover, *in vitro* and *in vivo* overexpression of wild-type or mutant α-synuclein in neurons results in protein aggregation and toxicity, thus leading to phenotypes resembling PD (Giasson et al., 2002; Kirik et al., 2002; Lo Bianco et al., 2002; Vekrellis et al., 2009). Similarly, overexpression of human wild-type α-synuclein in oligodendroglial cell lines (Stefanova et al., 2005; Kragh et al., 2009) or *in vivo* (Kahle et al., 2002; Shults et al., 2005; Yazawa et al., 2005; Stemberger et al., 2010) results in the formation of fibrillar α-synuclein forms, which may cause toxicity in oligodendrocytes and/or in neurons (in the animal models) or increase cell susceptibility to oxidative stress, thus recapitulating many features of MSA. Such data clearly demonstrate that the total protein load and aggregation of α-synuclein is a critical determinant of its neurotoxic potential, giving rise to the “α-synuclein burden hypothesis” which pinpoints a critical role of the protein in idiopathic PD pathogenesis, either through enhanced transcription or through impaired degradation (Vekrellis and Stefanis, 2012). There are conflicting results regarding the mRNA levels of α-synuclein in PD and MSA brains, probably due to the difficulty to maintain a proper RNA integrity in tissues undergone extensive neurodegeneration. Even though the factors controlling *SNCA* transcription *in vivo* remain largely unknown, a number of regulatory transcriptional elements have been identified in neuronal cells, such as the GATA-1 and -2 transcription factors that enhance *SNCA* transcription through binding in the Intron 1 region of *SNCA* gene (Scherzer et al., 2008). Subsequent studies surmised that a signal transduction pathway involving the MAPK 3 and PI3K pathways could be important for controlling *SNCA* transcription (Clough and Stefanis, 2007) and that the transcription factor ZSCAN21 (Zipro1) could play a significant role (Clough et al., 2009; Brenner et al., 2015; Dermentzaki et al., 2016; Lassot et al., 2018). Recently, the CCAAT/enhancer binding protein (C/EBP) δ was identified as a novel repressor of α-synuclein transcription, following its binding to the *SNCA* genomic region in both mice and humans (Valente et al., 2020). Post-transcriptional regulation through microRNAs (Junn et al., 2009; Doxakis, 2010; Surgucheva et al., 2013; Choi D.C. et al., 2018; Kim et al., 2018) or lncRNAs (Lin D. et al., 2018; Elkouris et al., 2019; Zou et al., 2020) has also been reported to alter *SNCA* mRNA levels.

The pathological effects of misfolded α-synuclein involve, amongst others, dysregulation of mitochondrial activity and endoplasmic reticulum (ER)-Golgi trafficking, plasma membrane integrity, synaptic vesicle trafficking and function of the ubiquitin-proteasome (UPS) and the autophagy-lysosome pathway (ALP) (Vekrellis et al., 2011). Overexpression of α-synuclein causes mitochondrial fragmentation (Kamp et al., 2010), event that is associated with an increase in mitochondrial fission rather than a fusion deficiency (Nakamura et al., 2011). Recently, it was reported that α-synuclein is normally localized at mitochondrial-associated membranes, while under pathological conditions α-synuclein dislocates from its sites and affects mitochondrial morphology (Guardia-Laguarta et al., 2014). Another study showed that under pathological conditions and in contrast with the native monomeric α-synuclein, aggregated forms of the protein preferentially bind to mitochondria, leading to mitochondrial dysfunction and cellular respiration limitation (Wang et al., 2019). Moreover, aggregated α-synuclein alters the membrane fusion and fission processes of mitochondria, resulting in fragmentation of the organelle and mitophagy inhibition (Chen and Chan, 2009). This mitochondrial damage is followed by a series of events, such as reactive oxygen species (ROS) production, electron leakage and caspase activation leading eventually to neuronal death (Ganjam et al., 2019). It has also been proposed that ROS production leads to a LRKK2-mediated impairment of endosomal and lysosomal function, resulting in pSer129 α-synuclein accumulation (Di Maio et al., 2018). Since pSer129 α-synuclein is considered an inhibitor of mitochondrial protein import, its aggregation is directly linked to mitochondrial senescence and ROS production, thus creating a positive feedback loop (Di Maio et al., 2018). Along with the mitochondrial dysfunction, α-synuclein aggregation also affects the activity of the ER, inducing protein-folding abnormalities, impaired ER-Golgi transport and calcium leakage, which ultimately lead to further aggregation of the protein (Volles and Lansbury, 2002; Thayanidhi et al., 2010; Colla et al., 2012; Colla, 2019). However, one of the main mechanisms through which α-synuclein causes neurotoxicity is the abnormal interaction of various protein assemblies with membranes, causing membrane disruption, lipid bilayer thinning and vesicle trafficking dysregulation (Hellstrand et al., 2013; Wang and Hay, 2015; Fusco et al., 2017). In the healthy brain, monomeric α-synuclein plays an important role in synaptic function; however, in pathological conditions, α-synuclein aggregation impairs the SNARE complex assembly, through its abnormal interaction with essential proteins for the synaptic vesicle cycle function (Dalfo et al., 2004; Gitler et al., 2008; Choi et al., 2013). It has been also shown that fibrillar α-synuclein may induce synaptic vesicle endocytosis and blockage of vesicle recycling, further contributing to the development of synaptopathy, which characterizes PD and related synucleinopathies (Nemani et al., 2010; Busch et al., 2014; Xu et al., 2016). Formation of aberrant α-synuclein species has been widely shown to impair the function of the UPS and the ALP pathway, as discussed below. Similar effects are observed in MSA where the pathological accumulation of α-synuclein in oligodendrocytes causes severe disruption of most cellular functions, with the myelination process being a major target of the protein’s aberrant effects. The demyelination, along with the reduction of trophic factors, leads to a secondary neuronal cell loss (Shults et al., 2005; Ubhi et al., 2010; Stefanova and Wenning, 2016).

Emerging evidence also suggest that activation of the inflammatory process due to the presence of abnormal α-synuclein species plays a central role in the development of synucleinopathies. Postmortem brain examination, brain imaging and animal studies converged that both the innate and adaptive immune systems are activated in PD contributing to disease progression (Tansey and Romero-Ramos, 2019). Activated microglia, which are considered the most efficient scavengers of extracellular α-synuclein aggregates (Lee et al., 2008b), increase the production of pro-inflammatory cytokines and induce an oxidative stress response (Klegeris et al., 2008; Su et al., 2008; Couch et al., 2011) even in the absence of neuronal loss (Sanchez-Guajardo et al., 2010; Barkholt et al., 2012; Watson et al., 2012). Increased pro-inflammatory mediators such as tumor necrosis factor alpha (TNF-α), interleukin-1-β (IL-1β), interleukin-6 (IL-6) have been shown in the cerebral spinal fluid (CSF) and in the striatum of human PD brains (Mogi et al., 1994a,b; Vawter et al., 1996; Nagatsu et al., 2000), supporting a chronic pro-inflammatory milieu in the brain of PD patients. Apart from α-synuclein mediated activation of microglia in the CNS, a more complex relationship between gut microbial-induced inflammation and α-synuclein expression and aggregation has been proposed to occur in the periphery (Chen S.G. et al., 2016; Choi J.G. et al., 2018). Clinical, epidemiological and animal studies suggest a complex cross-talk between intestinal inflammation and PD pathology initiation and progression (Houser and Tansey, 2017; Chen et al., 2019). Similarly, α-synuclein-evoked microglial activation is commonly detectable in the brains of MSA patients (Ishizawa et al., 2004) and MSA experimental models (Stefanova et al., 2007; Vieira et al., 2015; Monzio Compagnoni and Di Fonzo, 2019). Even though the activation of the inflammatory cascade in synucleinopathies may not represent a primary event but a secondary in response to other phenomena, it definitely contributes to the neuronal degeneration that characterizes these diseases.

## α-Synuclein and Protein-Degradation Pathways: A Complicated Relationship

**FIGURE 2:**
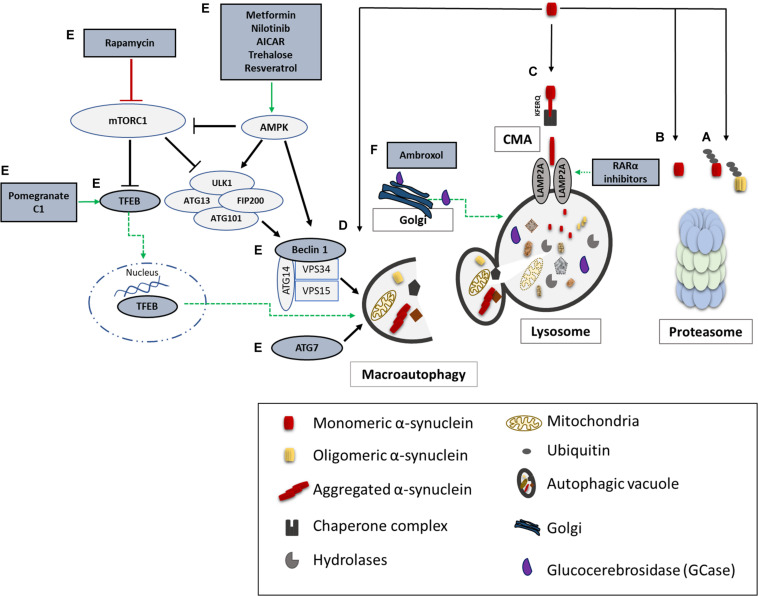
α-synuclein degradation pathways and reported therapeutic approaches to enhance protein clearance. A schematic representation of the main proteolytic pathways implicated in α-synuclein clearance and the proposed targets for potential therapeutic interventions (highlighted in dark grey and green). Wild type and mutant α-synuclein can undergo both ubiquitin-dependent **(A)** and ubiquitin-independent **(B)** degradation via the 20S/26S proteasome. Monomeric wild-type α-synuclein is degraded via CMA, following its binding to the CMA-specific receptor, LAMP2A **(C)**. Molecular upregulation of LAMP2A expression or chemical enhancement of CMA through retinoic acid receptor alpha (RARα) antagonists has been proven successful in alleviating α-synuclein-associated toxicity. Macroautophagy has been also proposed to degrade mutant and aggregated forms of α-synuclein **(D)**. Boosting macroautophagy via mTOR-dependent (rapamycin) or mTOR-independent pharmacological and nutritional modulators (Metformin, Nilotinib, AICAR, Trehalose, Resveratrol, Pomegranate, C1) enhance autophagosome formation, lysosome biogenesis, and lysosome function thus promoting α-synuclein clearance **(E)**. Molecular modulation of macroautophagy via Atg7, Beclin1 or TFEB overexpression is also reported to exert beneficial effects on α-synuclein-related toxicity **(E)**. Lastly, restoration of proper enzymatic activity of GCase has been shown to improve lysosomal function and lessen α-synuclein levels **(F)**.

Unraveling the pathway involved in the degradation of α-synuclein is crucial in understanding the pathogenetic mechanisms underlying its aberrant accumulation in synucleinopathies. Both the UPS and the ALP have been proposed to clear α-synuclein (Figure 2); however to a different extent and in a cell-, conformation- and tissue- specific manner (Bennett et al., 1999; Webb et al., 2003; Vogiatzi et al., 2008). Initial studies in purified systems and in neuronal cells, have demonstrated that α-synuclein can undergo both ubiquitin-dependent (Rott et al., 2011; Haj-Yahya et al., 2013) and ubiquitin-independent (Tofaris et al., 2001; Liu et al., 2003) degradation via the 26S/20S proteasome. Additional studies performed in PC12, HEK293 and primary mesencephalic cells failed to detect significant α-synuclein accumulation following pharmacological proteasomal inhibition (Rideout et al., 2001; Rideout and Stefanis, 2002; Vogiatzi et al., 2008). Others have found that only a small proportion of soluble-cell-derived intermediate α-synuclein oligomers, not including monomeric α-synuclein, are targeted to the 26S proteasome for degradation (Emmanouilidou et al., 2010b). In an elegant *in vivo* study it was shown that the UPS is the main degradation pathway for α-synuclein under normal conditions, while with increased α-synuclein burden the ALP is recruited (Ebrahimi-Fakhari et al., 2011). We and others have shown that only the wild-type α-synuclein and not the PD-linked A53T and A30P forms, the phosphorylated or the dopamine-modified α-synuclein, is degraded via the selective process of chaperone-mediated autophagy (CMA) (Cuervo et al., 2004; Martinez-Vicente et al., 2008; Vogiatzi et al., 2008; Mak et al., 2010). CMA can degrade only monomeric or dimeric forms of the protein, whereas macroautophagy is the only process that can clear oligomeric α-synuclein (Xilouri et al., 2013b). Not only mutations but also post-translational modifications such as phosphorylation, sumoylation and ubiquitination may also alter the partitioning of α-synuclein to proteasomal or lysosomal degradation (Xilouri et al., 2016b).

The UPS and the ALP not only degrade α-synuclein but can also be a direct target of the protein’s aberrant effects (Xilouri et al., 2016b; Zondler et al., 2017). Initial studies indicated that overexpression of the A30P and A53T mutants make cells more vulnerable to proteasomal inhibition-mediated cell death compared to cells overexpressing the wild-type protein (Tanaka et al., 2001). Additional evidence suggested that overexpression of mutant α-synuclein variants inhibits the activity of the 20S/26S proteasome leading to UPS failure thus, contributing to α-synuclein aggregation (Stefanis et al., 2001), although another study showed that overexpression of wild-type or mutant (A30P, A53T) α-synuclein in PC12 cells or in transgenic mice did not significantly affect proteasomal function (Martin-Clemente et al., 2004). More recently, it was shown that the function of the 20S proteasome was not affected upon administration of recombinant α-synuclein oligomers and fibrils or upon transient expression of wild-type or mutant α-synuclein (Zondler et al., 2017).

Studies in human post-mortem material also indicate that proteasome function is impaired in the substantia nigra of PD patients (Bentea et al., 2017), further cementing a role of a proper UPS function in PD pathogenesis. Beyond the UPS, increased α-synuclein has been reported to impair macroautophagy both *in vitro* and *in vivo* (Winslow et al., 2010), possibly through its interaction with Rab1a, which causes the mislocalization of Atg9, an autophagosome formation-related protein (Winslow et al., 2010). Similarly, Atg9 mislocalization and impaired autophagosome formation has been observed in cells expressing the PD-linked mutant form of the retromer protein VPS35 (Zavodszky et al., 2014). There is also evidence suggesting that the PD-linked α-synuclein mutations could have a different impact on macroautophagy machinery. In particular, the E46K α-synuclein mutation impairs autophagy at an early stage of autophagosome formation via dysregulation of the JNK1/Bcl2, an mTOR-independent pathway (Yan et al., 2014). Also, the A30P mutant α-synuclein inhibits autophagosome formation via activation of the autophagy transcriptional repressor ZKSCAN3 in a JNK-dependent manner (Lei et al., 2019). Furthermore, the A53T mutation has been shown to dysregulate mitophagy (Choubey et al., 2011), resulting in massive mitochondrial removal accompanied by bioenergetics deficits and neuronal degeneration. However, another study in A53T α-synuclein transgenic mice showed that α-synuclein accumulation leads to activation of the p38 MAPK pathway, which in turn directly phosphorylates Parkin thus inhibiting Parkin-mediated mitophagy (Chen et al., 2018). Several independent studies have proposed that autophagy is controlled by the GTP-ase–p38 MAPK signaling (Obergasteiger et al., 2018), a pathway that may be disturbed in PD. Additionally, mutations in the *GBA1* gene, which encodes for the lysosomal enzyme β-glucocerebrosidase (GCase) and cause Gaucher’s disease (GD), are among the most common known genetic risk factors for PD and DLB (Mata et al., 2008; Clark et al., 2009). Various studies in cell culture and animal models and in human post-mortem material suggest an inverse relationship between α-synuclein accumulation and GCase protein levels and activity where reduced GCase activity coincides with increased levels of α-synuclein (Mazzulli et al., 2011; Osellame et al., 2013; Sardi et al., 2013; Murphy et al., 2014; Du et al., 2015; Liu et al., 2015; Rocha et al., 2015; Chen M. et al., 2016).

The first connection between CMA and PD was established in 2004, where monomeric and dimeric wild-type α-synuclein species were shown to be CMA substrates, whereas the A30P and A53T PD-linked mutant α-synuclein forms bound more tightly to LAMP2A, CMA’s specific receptor, but were not up taken and degraded within lysosomes, thus becoming toxic by inhibiting the CMA-mediated degradation of other cytosolic substrate proteins (Cuervo et al., 2004). Subsequently, it was shown that post-translational modifications of wild-type α-synuclein, such as oxidation and nitration of the protein, alter its binding and uptake into lysosomes, while phosphorylation and dopamine-modification almost completely prevents its CMA-dependent degradation (Martinez-Vicente et al., 2008; Xilouri et al., 2009). Reciprocally, CMA inhibition is reported to lead to the formation of detergent-insoluble or high molecular weight oligomeric α-synuclein conformations *in vitro* (Vogiatzi et al., 2008; Xilouri et al., 2009), or to increased intracellular α-synuclein accumulation in nigral neurons *in vivo* (Xilouri et al., 2016b). Furthermore, the protein levels of the two key CMA markers LAMP2A and HSC70, were reported to be decreased in the human substantia nigra and amygdala of PD brains compared to controls (Alvarez-Erviti et al., 2010), while, more recently, decreased protein levels of LAMP2A correlated with increased α-synuclein accumulation were found in PD brain regions harboring α-synuclein pathology (anterior cingulate cortex) and not in other regions that are spared (occipital cortex) (Murphy et al., 2014). Importantly, these decreases correlated directly to CMA activity, since the protein levels of the other two *LAMP2* isoforms (2B and 2C) that do not participate in CMA, were found unaltered between PD and control brains (Murphy et al., 2015). Macroautophagy alterations have also been found in nigral neurons of PD brains (Anglade et al., 1997).

Beyond PD, evidence from human postmortem material from MSA brains suggests the possibility of macroautophagic alterations linked to α-synuclein accumulation in GCIs. More particularly, the detection of SQSTM1/p62 and, in some cases, of LC3 (but not of more mature lysosomal markers) and of the autophagic adaptor protein NBR1 within GCIs together with α-synuclein suggests a possible initial induction of macroautophagy and a subsequent defect in macroautophagy maturation in MSA brains (Chiba et al., 2012; Odagiri et al., 2012; Schwarz et al., 2012; Tanji et al., 2013). In addition, the FBXO27 gene, which encodes a protein associated with ubiquitination and protein degradation, was identified in a recent GWAS as a potential risk factor for MSA (Sailer et al., 2016). These neuropathological studies have also suggested the possibility of impaired proteasomal function as a driving force for GCI formation. Studies in oligodendroglial cells showed that, upon pharmacological proteasomal inhibition, p62 and LC3 accumulate in forming aggregates, in an apparent compensatory response of macroautophagy activation (Schwarz et al., 2012). In addition, dysfunctional macroautophagy evoked through mitochondrial impairment or macroautophagy inhibition resulted in the accumulation of α-synuclein in oligodendroglial cells (Pukass et al., 2015), whereas more recently it was reported that macroautophagy block through genetic or pharmacological inhibition of autophagy was inefficient to increase intracellular accumulation of α-synuclein in oligodendrocytes exposed to monomeric or fibrillar α-synuclein (Fellner et al., 2018). Nonetheless, additional work is needed to elucidate the precise role of UPS and ALP dysfunction in the accumulation of α-synuclein-rich GCIs in MSA brains.

## Cell-To-Cell Propagation of α-Synuclein Pathology in Synucleinopathies: The Strain Hypothesis

According to Braak staging, Lewy pathology manifested by positive α-synuclein inclusions spreads throughout the brain as PD progresses, primarily affecting the brainstem and olfactory system, thereafter gradually invading the neocortex (Braak et al., 2003). Amongst numerous studies that have tested this hypothesis (Braak et al., 2006; Lebouvier et al., 2010; Pouclet et al., 2012; Grathwohl et al., 2013; Paillusson et al., 2013; Holmqvist et al., 2014), recently it was shown that α-synuclein pre-formed fibrils (PFFs) injected into the duodenal and pyloric muscularis layer evoked a spread of pathologic Ser129 phosphorylated α-synuclein in various brain regions. Interestingly, truncal vagotomy prevented the gut-to-brain transmission of α-synuclein pathology, supporting the Braak hypothesis of a prion-like templating mechanism (Kim et al., 2019). Similarly, a cohort study of vagotomized patients supported that the vagus nerve is involved in the development of PD (Svensson et al., 2015). Recently, α-synuclein inclusions were detected in stomach and heart of a bacterial artificial chromosome (BAC) transgenic rat model injected into the gut wall of the pylorus and duodenum with α-synuclein PFFs. Their findings suggest a secondary anterograde (Dorsal Motor nucleus of the Vagus [DMV]-to-stomach) spreading of α-synuclein pathology, followed by a primary retrograde (duodenum-to-DMV) spreading (Van Den Berge et al., 2019).

However, there are studies presenting controversial results, suggesting that α-synuclein transmission from a peripheral injection site reaches the dorsal nucleus of vagus nerve, but does not further spread in the CNS (Manfredsson et al., 2018; Uemura et al., 2018). The hypothesis of α-synuclein prion-like propagation has gained attention in the recent years, since it has been shown that transplantation of healthy fetal mesencephalic neurons in PD patients led to the formation of LB-like inclusions, indicating the direct transfer of pathogenic α-synuclein from host brain to grafted neurons (Kordower et al., 2008; Li et al., 2008). Similar studies have verified that neurons inside the grafts were positive for LB-like α-synuclein aggregates (Li et al., 2008, 2010; Kordower et al., 2011; Kurowska et al., 2011). Moreover, inoculation of brain extracts from PD and DLB patients into the striatum and substantia nigra of mice (Masuda-Suzukake et al., 2013) and non-human primates induced α-synuclein aggregation and neurodegeneration (Recasens et al., 2014).

**FIGURE 3:**
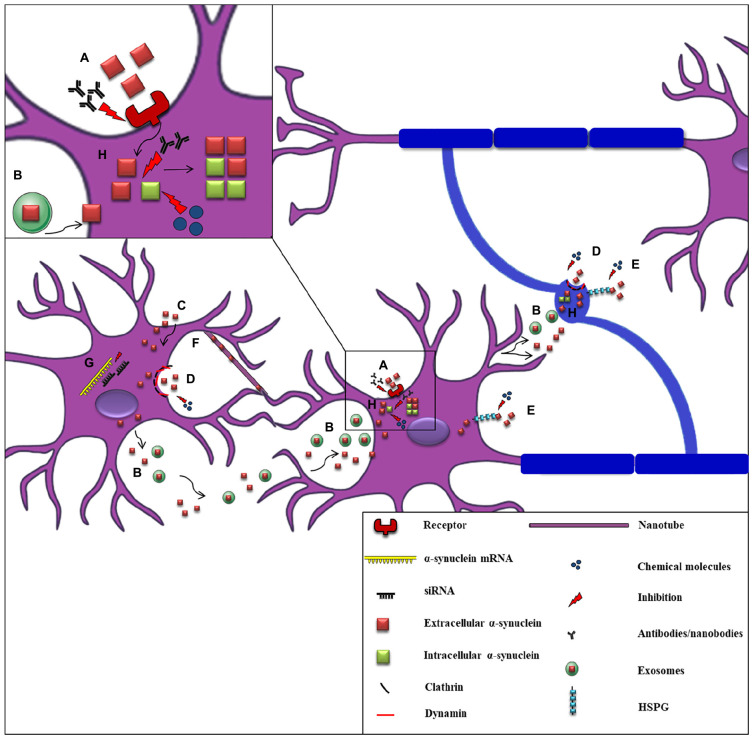
Proposed mechanisms of α-synuclein cell-to-cell propagation and application of candidate therapeutic strategies. A schematic representation of α-synuclein transfer from neurons to neurons (purple) or to oligodendrocytes (blue) via various mechanisms: **(A)** Neuronal receptors (i.e., LAG3) interact with extracellular α-synuclein and mediate its internalization via endocytosis. Antibodies against these receptors effectively inhibit α-synuclein propagation. **(B)** Neuronally-derived, free or exosomal-bound α-synuclein enters neighboring neurons or oligodendrocytes. **(C)** α-synuclein is taken-up by cells via passive diffusion or direct penetration of their plasma membrane. **(D)** Clathrin- or dynamin- mediated endocytosis is responsible for α-synuclein internalization in neurons and oligodendrocytes. Inhibitors targeting these endocytic pathways have been effectively used. **(E)** Heparan Sulfate ProteoGlycans (HSPGs) regulate α-synuclein uptake via macropinocytosis in neurons and oligodendrocytes. Disruption of HSPGs by chemical molecules (heparin or chloral hydrate) inhibits α-synuclein uptake by cells. **(F)** Tunneling nanotubes (thin membranous bridges) have been also proposed as a possible cell-to-cell transmission mechanism of α-synuclein. **(G)** siRNAs (small interfere RNAs) designed against α-synuclein mRNA are used for the reduction of α-synuclein production as an effective therapeutic strategy. **(H)** α-synuclein entrance in neuronal or oligodendroglial cells is followed by its aggregation and the spread of α-synuclein pathology (seeding), finally leading to the formation of aberrant protein species. Various antibodies targeting the NAC or the C-terminal region of α-synuclein and chemical molecules and compounds (i.e., NPT200–11, NPT100-18A, NPT088 etc.) inhibiting α-synuclein aggregation have been used to prevent α-synuclein misfolding.

The first clinical evidence to support α-synuclein spread throughout the nervous system was the detection of the protein in human CSF, indicating that α-synuclein can be released into the extracellular space (El-Agnaf et al., 2003). Subsequent studies verified the secretion of α-synuclein from neuronal cells, in part via vesicle- and exosomal-related trafficking (Lee et al., 2005; Emmanouilidou et al., 2010a; Jang et al., 2010; Danzer et al., 2012). On the other hand, conventional endocytosis (Sung et al., 2001; Lee et al., 2008a; Desplats et al., 2009; Hansen et al., 2011; Angot et al., 2012), exosomal transport (Emmanouilidou et al., 2010a; Delenclos et al., 2017), receptor-mediated internalization (Shrivastava et al., 2015; Mao et al., 2016; Ihse et al., 2017), passive diffusion (Ahn et al., 2006), or even direct penetration of the plasma membrane (Kayed et al., 2004; Jao et al., 2008; Lee et al., 2008a; Tsigelny et al., 2012) have been proposed as the main pathways for α-synuclein uptake (Figure 3). Amongst the receptors that have been proposed to mediate the uptake of the protein by neurons is the FcγRIIB inhibitory Fc receptor, which has been shown to be responsible for fibrillar α-synuclein cell-to-cell transmission mediated by the FcgRIIB-SHP-1/2 signaling (Choi Y.R. et al., 2018) and the LAG3 receptor reported to interact with fibrillar but not monomeric α-synuclein (Mao et al., 2016). Moreover, heparan sulfate proteoglycans (HSPGs) seem to regulate α-synuclein uptake via macropinocytosis in neurons, whereas GM1 ganglioside is responsible for α-synuclein entry in microglial cells (Park et al., 2009; Holmes et al., 2013). Fibrillar α-synuclein has been shown to interact *in vitro* with HSPGs via specific sequence motifs and thus be effectively endocytosed (Zhang et al., 2020). Recent data revealed that N-linked glycans on the cell surface of neurons interact with acetylated α-synuclein and mediate its internalization and subsequent pathological aggregation (Birol et al., 2019).

It has also been reported that neurons can take up naked α-synuclein following it’s binding to specific membrane proteins, which are then partially localized within the lysosomal compartment (Lee et al., 2008a; Karpowicz et al., 2017). Disruption of the lysosomal function has been shown to play an important role in the transmission of α-synuclein pathology and the subsequent neurodegeneration (Xilouri et al., 2016a; Jiang et al., 2017; Klein and Mazzulli, 2018; Minakaki et al., 2018). Importantly, in cases of compromised cell proteolytic machineries, vesicle-associated α-synuclein release seems to be enhanced (Jang et al., 2010; Alvarez-Erviti et al., 2011; Danzer et al., 2012; Lee et al., 2013; Fussi et al., 2018). However, cell-to-cell spread of α-synuclein pathology does not necessarily require cell contacts, since tunneling nanotubes may also represent a possible transmission mechanism (Abounit et al., 2016; Figure 3). Exogenous α-synuclein, once taken up by neuronal or glial cells is directed to their cytosol where it recruits the aggregation of the endogenous α-synuclein into the formation of aberrant species, via an up-to-date unknown mechanism (Volpicelli-Daley et al., 2014; Rey et al., 2016; Karampetsou et al., 2017; Karpowicz et al., 2017; Kaji et al., 2018; Luna et al., 2018; Mavroeidi et al., 2019). Many *in vitro* and *in vivo* studies have proposed that exogenously added human α-synuclein PFFs are used as a template and recruit the endogenous soluble monomeric α-synuclein into the formation of insoluble LB-like inclusions (Luk et al., 2009, 2012a; Volpicelli-Daley et al., 2011; Masuda-Suzukake et al., 2013; Sacino et al., 2013a, 2014a,b; Wu et al., 2019). Similar results were obtained *in vivo* when PD brain- or symptomatic α-synuclein transgenic mice-derived homogenates were delivered in the living brains of mice and monkeys (Mougenot et al., 2012; Recasens et al., 2014; Schweighauser et al., 2015).

Two seeding processes have been demonstrated to govern the aggregation of the endogenous α-synuclein: homotypic (or self-seeding) and heterotypic seeding. The term “homotypic seeding” is referred to the sequence-specific templating of α-synuclein that requires the presence of the hydrophobic NAC region (El-Agnaf et al., 1998; Giasson et al., 2001). On the other hand, heterotypic seeding is a process that involves other proteins (such as tau, huntingtin and Aβ) in the initiation of α-synuclein recruitment and fibrillization (Charles et al., 2000; Masliah et al., 2001; Giasson et al., 2003; Pletnikova et al., 2005; Badiola et al., 2011; Tomas-Zapico et al., 2012). Many studies utilizing the α-synuclein PFF-brain inoculation as a PD model, suggest the “connectomic” transmission of pathological α-synuclein from the injection site to various interconnected brain regions (Luk et al., 2012a; Masuda-Suzukake et al., 2013, 2014; Paumier et al., 2015; Peelaerts et al., 2015; Ulusoy et al., 2015; Rey et al., 2016) and that this α-synuclein spread based on the anatomical connections between brain areas (Luk et al., 2012b; Rey et al., 2013, 2016; Peelaerts et al., 2015), party occurs via gap junction channels composed of connexins, formed between adjacent cells (Diaz et al., 2019; Reyes et al., 2019). Finally, a recent study pinpoints 14-3-3 proteins as potential regulators of α-synuclein transmission, proposing that they normally prevent α-synuclein oligomerization and resultant toxicity, whereas 14-3-3 protein dysfunction mediates α-synuclein oligomerization and seeding, that govern PD pathology (Wang et al., 2018). Numerous *in vitro* and *in vivo* studies utilizing α-synuclein PFFs as seeds, have proposed an induction in the endogenous neuronal α-synuclein aggregation, thus favoring the prion-like hypothesis of α-synuclein spread (Luk et al., 2009, 2012a; Volpicelli-Daley et al., 2011; Masuda-Suzukake et al., 2013; Sacino et al., 2014b; Paumier et al., 2015; Peelaerts et al., 2015; Abdelmotilib et al., 2017). Interestingly, not only PFFs but also isolated exosomes from the CSF of PD and DLB patients, containing pathological species of α-synuclein, were able to transfer the disease pathology when applied in human H4 neuroglioma cells (Stuendl et al., 2016).

Beyond PD, the self-propagation of α-synuclein resulting in the formation of insoluble aggregates within the cytoplasm of oligodendrocytes termed MSA also as a prion-like disease. Prusiner et al. originally reported that TgM83^+/–^ mice expressing human A53T α-synuclein when inoculated with MSA, but not PD, brain homogenates developed neurodegeneration, suggesting that a distinct strain of α-synuclein displays prion characteristics during the development of MSA (Watts et al., 2013; Prusiner et al., 2015). Accordingly, treatment of HEK293T cells stably expressing fluorescently-tagged α-synuclein with either healthy-control brain extracts or brain samples derived from PD or MSA patients, revealed that only in the case of MSA-added material, cells developed α-synuclein accumulation (Woerman et al., 2015). The prion-like transmission of α-synuclein pathology was further supported by intrastriatal delivery of MSA homogenates in the brain of human-wild type α-synuclein-expressing mice and detection of pathological α-synuclein aggregates formed in many brain regions (Bernis et al., 2015). Two possible scenarios have been proposed to explain the origin of α-synuclein in oligodendrocytes and the mechanisms underlying α-synuclein accumulation in GCIs present in MSA brains: either oligodendrocytes pathologically overexpress α-synuclein in the context of MSA (Asi et al., 2014) or they take up neuronally derived protein from their environment (Kisos et al., 2012; Konno et al., 2012; Rockenstein et al., 2012; Ettle et al., 2014; Pukass and Richter-Landsberg, 2014; Reyes et al., 2014; Pukass et al., 2015; Kaji et al., 2018). We have recently suggested that the presence of endogenous oligodendroglial α-synuclein, however minute in amount, is a critical factor for the generation of pathological GCI-like α-synuclein structures within oligodendrocytes and myelin dysregulation evoked by PFF-administration both *in vitro* and *in vivo* (Mavroeidi et al., 2019).

Furthermore, as already mentioned, neuroinflammation is a key mediator of α-synuclein-related toxicity, since microglia and astroglia activation, gliosis and increased secretion of pro-inflammatory factors are often observed in synucleinopathies (Nagatsu and Sawada, 2005; Politis et al., 2012; Tufekci et al., 2012; Fellner and Stefanova, 2013; Kaufman et al., 2013). Many studies focused on α-synuclein transfer between neurons and glial cells (Lee et al., 2010; Sacino et al., 2013b; Reyes et al., 2014). It has been proposed that α-synuclein attracts and activates microglia; however these cells fail to clear α-synuclein, thus leading to an excessive pro-inflammatory response via Toll-like receptor (TLR) and finally to neurodegeneration (Stefanova et al., 2011; Kim et al., 2013; Roodveldt et al., 2013; Tang and Le, 2016). A recent study suggests that the differential activation state of microglial cells plays a crucial role in neuron-to-neuron α-synuclein spread and that IL-4-activated microglia seems to engulf extracellular α-synuclein, thus reducing neuron-to-neuron α-synuclein transmission (George et al., 2019). Others have suggested that pathological α-synuclein spreads through tunneling nanotubes in macrophages and possibly in microglial cells as well (Onfelt et al., 2004). The neuron-to-astrocyte and astrocyte-to-astrocyte transmission of α-synuclein has been shown both in *in vitro* and *ex vivo* experiments, however α-synuclein aggregates are preferably formed within neurons rather than in astrocytes (Lee et al., 2010; Loria et al., 2017). This fact could be attributed to the protective role of astrocytes against α-synuclein aggregation, via enhanced proteolytic processing of exogenously added PFFs (Loria et al., 2017).

Interestingly, it has been proposed that α-synuclein joins into distinct polymorphisms possibly responsible for the variety of disease phenotypes giving rise to the strain hypothesis, underlying the pathogenesis of synucleinopathies. Based on this scenario, the central idea is that α-synuclein fibrils behave as strains with discrete biochemical and structural characteristics, into distinct brain regions and cell types (Cremades et al., 2012; Bousset et al., 2013; Kim et al., 2016; Ma et al., 2016; Pieri et al., 2016; Lau et al., 2020). Supportive of this hypothesis are findings showing that pathological α-synuclein in PD and MSA inclusions is conformationally and biologically distinct and different α-synuclein strains are generated in discrete intracellular milieus (Peng et al., 2018a). We have also shown that the pathology-related S129 α-synuclein phosphorylation in primary cultures and human post-mortem brain material might involve different α-synuclein strains present in oligodendroglial and neuronal synucleinopathies (Mavroeidi et al., 2019).

Most importantly, it is reported that α-synuclein fibrils amplified from the brains (Strohaker et al., 2019) or CSF (Shahnawaz et al., 2020) of PD patients are structurally different than those from MSA, further suggesting that distinct conformational strains of α-synuclein may underlie the different pathology detected in two synucleinopathies. Therefore, it is surmised that based on the diversity of the human strains and via the protein misfolding cyclic amplification (PMCA) technique, we can discriminate between PD and MSA pathology in patient-derived CSF samples with high sensitivity (Shahnawaz et al., 2020). These strains can recruit and seed endogenous α-synuclein, and also propagate by imprinting their unique structural properties on its non-pathogenic counterpart (Bousset et al., 2013; Peelaerts et al., 2015; Mavroeidi et al., 2019). Through their neurotoxic behavior as oligomeric or fibrillar assemblies, α-synuclein strains are the crucial pathogens responsible for the induction of α-synuclein - and tau-specific disease phenotypes (Guo et al., 2013; Peelaerts et al., 2015; Candelise et al., 2019). In addition, it has been recently reported that MSA strains show several similarities with PD strains, but are significantly more potent in inducing motor deficits, nigrostriatal neurodegeneration, α-synuclein pathology, spreading, and inflammation, reflecting the aggressive nature of this disease (Van der Perren et al., 2020). In contrast, DLB-amplified strains displayed very modest neuropathological features.

## Therapeutic Approaches to Halt α-Synucleinopathy

Unfortunately, up-to-date no disease-modifying therapies exist for α-synucleinopathies only symptomatic therapies to relief motor impairment, including dopamine replacement, deep brain stimulation and pharmacological treatment of non-motor symptoms (Aarsland et al., 2002; Fahn et al., 2004; Ravina et al., 2005; Burn et al., 2006; Okun, 2012). Given the critical role of α-synuclein levels to disease pathogenesis, one obvious approach is to curtail total protein levels, either by reducing protein production or by enhancing protein degradation. Another approach would be to inhibit protein aggregation and misfolding or to alter α-synuclein post-translational modifications such as phosphorylation, which are suggested to affect the aggregation process and the development of toxic species. Targeting the extracellular levels of the protein with antibodies and intervening in the proposed mechanisms of uptake is also an attractive approach, since this may combat α-synuclein propagation and disease progression.

## Reducing α-Synuclein Production

Decreasing the production and the cytoplasmic levels of α-synuclein with the use of RNA interference (RNAi) technology represents an attractive approach for therapy in synucleinopathies (Figure 3). Specifically, there are *in vivo* studies showing that delivery of either naked small interfering RNAs (siRNAs) or lentiviral-mediated RNAi for *SNCA* silencing in the rodent brain, can effectively reduce α-synuclein levels (Sapru et al., 2006; Lewis et al., 2008). Accordingly, similar siRNA delivery in the substantia nigra of squirrel monkeys led to a significant suppression of α-synuclein expression with no toxic effects in the animal physiology (McCormack et al., 2010). Another study also suggested a non-toxic but rather a neuroprotective role of α-synuclein reduction in rats injected with shRNAs against *SNCA* via adeno-associated virus (AAV) (Zharikov et al., 2015).

*In vitro* and *in vivo* experiments using an amido-bridged nucleic acid (AmNA)-modified antisense oligonucleotide (ASO) resulted in decreased mRNA and protein levels of α-synuclein and improved motor deficits in a PD mouse model (Uehara et al., 2019). In addition, AAV-mediated delivery of an anti- α-synuclein ribozyme (rAAV-SynRz) prevented the death of the nigral dopaminergic neurons in the rat MPTP intoxication model (Hayashita-Kinoh et al., 2006). The rescue of dopaminergic cells loss via gene silencing has also been studied using an AAV harboring a short-hairpin (sh)RNA targeting human *SNCA* in the rat striatum, previously injected with AAV-hSNCA for human α-synuclein overexpression (Khodr et al., 2011). *In vitro* studies from the same group revealed that the miR-30-embedded shRNA silencing vector successfully decreased α-synuclein levels (Han et al., 2011); yet delivery of AAV-mir30-hSNCA in the rat brain did not produce encouraging results towards a potential PD therapy (Khodr et al., 2014).

However, there are controversial studies that present degenerative outcomes following a reduction of α-synuclein levels in the substantia nigra of rats and non-human primates (Gorbatyuk et al., 2010; Kanaan and Manfredsson, 2012; Collier et al., 2016; Teki and Griffiths, 2016; Benskey et al., 2018). siRNA-mediated reduction of α-synuclein has been also shown to regulate dopamine release in SH-SY5Y cells and to be used as a protective mechanism against MPP^+^-induced neurotoxicity (Fountaine and Wade-Martins, 2007). Moreover, exosomal-associated siRNA intravenous-delivery targeting α-synuclein mRNA in both wild type and α-synuclein transgenic mice, prevented protein aggregation (Cooper et al., 2014). Additionally, α-synuclein knockdown in the brain of wild-type mice, using siRNAs and an anti-sense oligonucleotide molecule (ASO), did not display any harmful effects on neuronal function (Alarcon-Aris et al., 2018). Finally, beta-2-adrenoreceptor (beta-2AR) ligands were shown to modulate *SNCA* transcription through histone 3 lysine 27 acetylation of *SNCA* promoter and enhancers. Beta-2AR agonists (clenbuterol and salbutamol) when used in various cellular and *in vivo* models, led to the reduction of α-synuclein expression, whereas beta-2AR antagonist propranolol increased *SNCA* transcription and α-synuclein production (Mittal et al., 2017).

## Enhancing α-Synuclein Degradation

Given the well-recognized role of ALP in α-synuclein degradation (Figure 2) the mammalian target of rapamycin (mTOR) has emerged as a therapeutic target for PD. Towards this direction, rapamycin, an inhibitor of mTOR, was shown to reduce α-synuclein accumulation in WT, A30P and A53T α-synuclein-overexpressing PC12 cells (Webb et al., 2003), to attenuate dopaminergic degeneration in neurotoxin-induced (Dehay et al., 2010; Malagelada et al., 2010; Liu et al., 2013) and α-synuclein-overexpressing PD models (Crews et al., 2010) and to improve motor function in A53T α-synuclein transgenic mice (Bai et al., 2015). However, a considerable limitation of rapamycin is that it interferes with numerous other autophagy independent pathways, including immunosuppression (Staats et al., 2013) and that prolonged exposure to rapamycin inhibits mTORC2 (Schreiber et al., 2015), thus leading to the stimulation of other important cellular pathways. To overcome these unwanted side effects, studies focused on TFEB, a down-stream target of mTOR, and showed that TFEB overexpression promoted the clearance of pathologic α-synuclein and restored neurodegeneration in PD animal models (Dehay et al., 2010; Decressac et al., 2013). Similarly, oligodendroglial-targeted TFEB overexpression efficiently prevented α-synuclein accumulation and rescued nigrostriatal neurodegeneration in the PLP-α-synuclein MSA mouse model (Arotcarena et al., 2019).

Moreover, several compounds associated with the activation of the AMP-activated protein kinase (AMPK)-dependent autophagy, such as metformin (Dulovic et al., 2014; Patil et al., 2014; Lu et al., 2016), or nilotinib (Hebron et al., 2013; Karuppagounder et al., 2014; Mahul-Mellier et al., 2014), have been reported to inhibit α-synuclein accumulation and to exert neuroprotection in several PD models. The tyrosine-kinase inhibitor nilotinib -a medication widely used for the treatment of chronic myelogenous leukemia- has now been repurposed for the treatment of PD and a Phase 1 clinical trial in 11 PD and DLB patients showed cognitive and motor improvement following nilotinib administration (NCT02281474). A larger Phase 2 clinic trial including 75 PD patients is currently being conducted with so far promising results (NCT02954978). Comparable findings have been reported upon application of 5-aminoimidazole-4-carboxamide ribonucleotide (AICAR) (a drug used for the treatment of acute lymphoblastic leukemia) in various PD models (Ng et al., 2012; Dulovic et al., 2014). Trehalose, also leads to an AMPK-dependent and mTOR-independent induction of autophagosome biogenesis and has been also shown to exert beneficial effects on cell survival and autophagy-dependent α-synuclein clearance in cellular (Sarkar et al., 2007; Casarejos et al., 2011; Lan et al., 2012) and animal PD models (Sarkar et al., 2014; Tanji et al., 2015; Wu et al., 2015; He et al., 2016). Collectively, given the pleiotropic actions and the limited specificity of these agents for the autophagic process, it’s challenging to determine whether the observed beneficial effects are mediated by AMPK activation or are a result of other off-target effects, which limit their potential therapeutic utility in synucleinopathies.

In search of non-chemical methods to modulate autophagy, experimental evidence shows that the natural inducer of autophagy curcumin, counteracted the accumulation of the A53T α-synuclein through down-regulation of the mTOR/p70S6K signaling pathway in SH-SY5Y cells (Pandey et al., 2008; Jiang et al., 2013) and conferred neuroprotection in rotenone-treated dopaminergic neurons (Satish Bollimpelli and Kondapi, 2015). A curcumin analog, C1, has been recently identified as a novel mTOR-independent activator of TFEB (Song et al., 2016), resulting in enhanced autophagy and increased lysosome biogenesis in the rat brain (Song et al., 2016). Likewise, the natural compound pomegranate has been shown to enhance TFEB activity and activate mTOR-independent autophagy and mitophagy (Tan et al., 2019). The natural plant phenol resveratrol, possibly following the interaction with its direct target SIRT1, was also shown to induce an AMPK-dependent autophagy and exert beneficial effects in several *in vitro* and *in vivo* PD models (Wu Y. et al., 2011; Ferretta et al., 2014; Lin et al., 2014; Guo et al., 2016; Ur Rasheed et al., 2016). A major concern of broad macroautophagy enhancement is the multifunctional role and status of activation in different cell types and tissues and the fact that excessive stimulation of macroautophagy under specific circumstances can exert detrimental effects (Yang et al., 2007; Choi et al., 2010; Xu et al., 2014). We have previously shown that CMA inhibition conferred by aberrant α-synuclein overexpression in neuronal cells resulted to a compensatory induction of macroautophagy and subsequent death, whereas pharmacological and molecular macroautophagy inhibition exerted a protective effect (Xilouri et al., 2009). Furthermore, the activation of mitophagy in primary cortical neurons overexpressing A53T α-synuclein caused mitochondrial destruction and neuronal degeneration that could be rescued by inhibition of macroautophagy (Choubey et al., 2011).

Another promising approach implicates the restoration of proper GCase activity as means to facilitate α-synuclein degradation. *In vivo* studies demonstrated that enhancing GCase activity (either pharmacologically or molecularly) could prevent or diminish formation of toxic α-synuclein species and related toxicity (Sardi et al., 2011, 2013). Enzyme-replacement therapies for GD showed that GCase does not cross the blood-brain barrier (BBB), therefore, recent strategies focused on the development of small-molecule chaperones to correct the folding of GCase, enhance GCase activity and restore lysosomal function to facilitate α-synuclein clearance (Schapira and Gegg, 2013; Blanz and Saftig, 2016). Ambroxol is a drug commonly used as an anti-mucolytic respiratory agent (Malerba and Ragnoli, 2008) and has been shown to restore lysosomal function and reduce oxidative stress in *GBA1* mutant fibroblasts (McNeill et al., 2014; Ambrosi et al., 2015). More recently, ambroxol treatment was found to increase GCase levels, improve autophagy and decrease α-synuclein levels in neural crest stem cell-derived dopaminergic neurons from *GBA1* mutation patients (Yang et al., 2017) and α-synuclein transgenic mice (Migdalska-Richards et al., 2016). The same group reported that oral administration of ambroxol increased GCase activity in the non-human primate brain indicating that ambroxol represents a promising novel disease modifying therapy for the treatment of PD and neuropathic GD (Migdalska-Richards et al., 2017). A phase 2 clinical trial assessing the safety and efficacy of ambroxol to improve motor and cognitive features of PD-GD patients has been recently completed (NCT02914366) (Silveira et al., 2019). Moreover, another clinical trial of PD patients (with or without GBA1 mutations) treated with up to 420 mg/day of ambroxol at 5 intra-dose escalations over the course of 6 months, confirmed the safety, tolerability and CSF penetration of this drug (NCT02941822) (Mullin et al., 2020). Furthermore, treatment with LTI-29, another activator of GCase activity, has been shown to reduce glucosylceramide levels *in vivo*^1^ and the safety and tolerability of this therapeutic candidate are being tested in a phase 1 clinical trial in *GBA*-PD patients (NTR6960, EudraCT2017 004086 27). Finally, new glucosylceramide synthase inhibitors capable of crossing the BBB and prevent the substrate buildup in mouse models have arisen as another strategy for intervention (Sardi et al., 2017, 2018). Toward this direction, a phase 1 clinical trial assessing the safety and tolerability of the glycosylsynthase inhibitor Venglustat (GZ/SAR402671) in *GBA*-PD patients has successfully been completed (NCT02906020) and this therapeutic approach is currently into phase 2 (Judith Peterschmitt et al., 2019).

Restoration of CMA activity could also provide therapeutic benefit in synucleinopathies, by not only promoting the clearance of α-synuclein, but also by mitigating its detrimental effects on lysosomal function. To this end, we have shown that overexpression of LAMP2A, CMA’s rate-limiting step in human neuroblastoma SH-SY5Y cells, rat primary cortical neurons and nigral dopaminergic neurons *in vivo*, was capable of alleviating α-synuclein-related toxicity (Xilouri et al., 2013a). Similarly, LAMP2A overexpression promoted autophagic flux and prevented α-synuclein-induced PD-like symptoms in the Drosophila brain (Issa et al., 2018). Moreover, pharmacological manipulation of the CMA pathway using AR7, a retinoic acid receptor alpha (RARα) antagonist, in LRRK2^R1441G^ mutant mouse fibroblasts restored the impaired lysosomal function and attenuated the progressive accumulation of both intracellular and extracellular α-synuclein oligomers, surmising that CMA activation could successfully prevent the accumulation of such species (Ho et al., 2020). Based on the findings supporting that pathogenic forms of α-synuclein lead to abnormal LAMP2A binding and disruption of the receptor’s assembly (Cuervo et al., 2004; Martinez-Vicente et al., 2008) modulation of LAMP2A dynamics at the lysosomal membrane may also represent a fruitful strategy. CMA activity is regulated by the lysosomal mTORC2/PHLPP1/Akt axis (Arias et al., 2015), suggesting that available drugs acting as inhibitors of mTORC2 or Akt, or as activators of PHLPP1 that can modulate the assembly/disassembly rate of the LAMP2A translocation complex could become attractive targets for selective modulation of CMA.

## Inhibiting α-Synuclein Aggregation

Prevention of α-synuclein aggregation and misfolding is a key player in disease confronting (Figure 3). The selective specificity of intrabodies/nanobodies allows them to bind to specific regions of different α-synuclein species (monomers, oligomers, fibrils) and modulate aggregation, therefore attenuating the disease pathology (Bhatt et al., 2013). The nanobodies VH14^∗^PEST and NbSyn87^∗^PEST target the NAC and the C-terminal regions of α-synuclein, respectively, and have been efficiently used against the formation of pathological pSer129 α-synuclein species following their delivery using viral vectors in the rat substantia nigra (Chatterjee et al., 2018). Three small molecules, NPT200–11, NPT100-18A and NPT088 have been reported as inhibitors of either oligomeric or proteinase K-resistant α-synuclein aggregates, both *in vitro* and *in vivo* (Krishnan et al., 2014; Wrasidlo et al., 2016; Price et al., 2018). Moreover, an emerging number of compounds tested in cellular and mouse models of PD exerted a protective role against α-synuclein pathology. Specifically, polyphenol (-)-epi-gallocatechin-3-gallate (EGCG) is used in both AD and PD cases and acts as an inhibitor of α-synuclein and amyloid beta fibril-maturation, by converting large amyloid fibrils into smaller non-toxic aggregates (Bieschke et al., 2010). Anle138b [3-(1,3-benzodioxol-5-yl)-5-(3-bromophenyl)-1H-pyrazole] is an oligomer modulator shown to prevent the formation of pathological aggregates *in vitro* and *in vivo* of both prion protein PrP(Sc) and α-synuclein (Wagner et al., 2013; Levin et al., 2014). Behavioral and histological analysis of the PLP-α-synuclein transgenic mice treated with anle138b revealed that this aggregation inhibitor effectively attenuated the progression of the MSA-like pathology (Fellner et al., 2016; Heras-Garvin et al., 2019). Interestingly, this compound has gained attention as a promising fluorescent biomarker for the detection of aggregation-related epitopes (Deeg et al., 2015).

CLR01, another aggregation-inhibitor, prevented the formation of β-sheet-rich fibrils and had beneficial effects on the health and survival of a zebrafish model of α-synuclein toxicity (Prabhudesai et al., 2012). Moreover, when PLP- or Thy1-α-synuclein transgenic mice received treatment with CLR01, they displayed amelioration of the α-synuclein-related brain pathology and behavioral deficits (Richter et al., 2017; Herrera-Vaquero et al., 2019). Furthermore, KYP-2047, a prolyl oligopeptidase inhibitor, has also been effectively used against α-synuclein aggregation in both cellular and mouse models of PD (Myohanen et al., 2012), whereas porphyrin phthalocyanine tetrasulfonate, an inhibitor of protein aggregation through binding to vesicle-associated α-synuclein, is suggested to modulate α-synuclein misfolding and toxicity (Fonseca-Ornelas et al., 2014). There are also numerous chemical compounds belonging to polyphenols, phenothiazines, polyene macrolides, porphyrins, rifamycins, Congo red (and its derivatives) and terpenoids, that have been shown to decrease α-synuclein fibrillization (Masuda et al., 2006). Baicalein (flavone), delphinidin (anthocyanidin) and methylthioninium (monoamine oxidase inhibitor) are chemical molecules with inhibitory properties against α-synuclein filament formation (Zhu et al., 2004; Hung et al., 2016; Schwab et al., 2017; Javed et al., 2018) as proposed by *in vitro* and *in vivo* experiments. Similarly, mannitol, catechol-o-methyltransferase inhibitors, cinnamon extract, and ring-fused pyridones have anti-aggregatory properties and provide protection against α-synuclein toxicity (Di Giovanni et al., 2010; Shaltiel-Karyo et al., 2012, 2013; Horvath et al., 2013).

Synthetic peptides are also another therapeutic approach developed for β-sheet structure disruption and inhibition of α-synuclein accumulation (El-Agnaf et al., 2004; Kim et al., 2009; Shaltiel-Karyo et al., 2010). Moreover, the antibiotic rifampicin has been used as a destabilizer of α-synuclein fibrils (Li et al., 2004) and a reduction in monomeric, oligomeric and pathological pSer129 α-synuclein has been reported in a rifampicin-treated transgenic MSA mouse model (Ubhi et al., 2008). Rifampicin has been also tested in a clinical trial where 50 participants received a 12-month treatment with rifampicin (600 mg/day), with however, negative results (Low et al., 2014). Another anti-aggregation therapeutic strategy in the context of MSA would be the inhibition of β-III tubulin and the oligodendroglial-specific phosphoprotein TPPP/p25α, since both proteins are implicated in α-synuclein accumulation (Lindersson et al., 2005; Nakayama et al., 2012; Mavroeidi et al., 2019). Nocodazole, a synthetic tubulin-binding agent that inhibits tubulin polymerization, prevented α-synuclein accumulation in primary neuronal and glial cultures, pinpointing the crucial role of β-III tubulin/α-synuclein interaction in MSA pathogenesis (Nakayama et al., 2012). Unfortunately, up-to-date there are no available p25α inhibitors, although such an approach may exert beneficial effects against MSA.

## Targeting α-Synuclein Post-Translational Modifications

Taking into account that post-translational modifications of α-synuclein, such as phosphorylation, truncation or oxidation/nitration, are tightly associated with the development of neuropathology (Oueslati et al., 2010; Barrett and Timothy Greenamyre, 2015), modulating these modifications is another viable approach. Although the majority of studies link pSer129-α-synuclein with neuropathology (Smith et al., 2005; Chau et al., 2009; Ma et al., 2016; Grassi et al., 2018), others support that α-synuclein phosphorylation and the subsequent inclusion formation protects cells from toxicity (Chen and Feany, 2005; Paleologou et al., 2008; Wu B. et al., 2011; Kuwahara et al., 2012). Therefore, regulation of the expression and/or activity of kinases and phosphatases responsible for phosphorylation and de-phosphorylation of α-synuclein at Ser129 represent a main target. Specifically, overexpression of G-protein-coupled receptor kinase 6 (GRK6) proposed to phosphorylate α-synuclein via AAVs in the rat substantia nigra, led to extensive degeneration of dopaminergic neurons (Sato et al., 2011). Towards the same direction, mutation of Ser129 to alanine inhibited the G protein-coupled receptor kinase 2 (Gprk2)-mediated phosphorylation of α-synuclein and attenuated α-synuclein toxicity in a PD transgenic fly model (Chen and Feany, 2005). Moreover, *in vitro* and *in vivo* enhancement of α-synuclein de-phosphorylation via the phosphoprotein phosphatase-2A (PP2A) protected neurons against α-synuclein pathology (Lee et al., 2011).

Many studies have also proposed that both C- and N-terminal truncations of α-synuclein facilitate α-synuclein aggregation and misfolding and exhibit pathological properties (Ulusoy et al., 2010; Hall et al., 2015; Wang et al., 2016; Iyer et al., 2017; Ma et al., 2018; Terada et al., 2018). Monoclonal antibodies against the C-terminal truncation (1H7, 5C1, and 5D12) have been tested in a PD mouse model and the results showed reduced aggregation of α-synuclein and improved neurotoxic and behavioral deficits upon immunotherapy (Games et al., 2014). Other studies focused on calpain targeting by using specific calpain inhibitors *in vivo*, a strategy that resulted in amelioration of α-synuclein pathology (Diepenbroek et al., 2014; Hassen et al., 2018). However, enhancement of calpain activity did not result in the expected exacerbation of α-synuclein pathology (Diepenbroek et al., 2014).

## Reducing Uptake and Cell-To-Cell Transmission of α-Synuclein

As mentioned above, one proposed mechanism for α-synuclein uptake is endocytosis and inhibiting the protein endocytosis represents another strategy against α-synuclein propagation and spread (Figure 3). For example, experiments in α-synuclein-treated cells have shown that deletion of LAG3 or use of antibodies raised against this neuronal receptor effectively inhibits α-synuclein cell-to-cell transfer (Anderson et al., 2016; Mao et al., 2016). Moreover, heparin or chloral hydrate can also act as α-synuclein fibril uptake inhibitors, via disruption of heparan sulphate proteoglycans that normally bind amyloid proteins (Holmes et al., 2013; Karpowicz et al., 2017). Taking into consideration the fact that prion protein (PrPC) is suggested to be implicated in amyloid α-synuclein uptake and neurotoxicity, genetic knockout of PrPC in primary neurons and mice effectively reduced the uptake and aggregation of α-synuclein (Aulic et al., 2017; Ferreira et al., 2017).

Another therapeutic approach to combat α-synuclein cell-to-cell transmission is to target the signaling pathway downstream of the receptor responsible for α-synuclein uptake. For example, the inflammatory response of microglial cells to pathological α-synuclein via TLR signaling can be ameliorated by using TLR4 signaling inhibitors, such as resatorvid (TAK242) (Ii et al., 2006; Kawamoto et al., 2008). In another study, treatment of cells with TAK242 or RSLA (another TLR4 inhibitor) resulted in reduction of TNFα secretion from microglial cells and protection of neuronal cells against α-synuclein neurotoxicity (Hughes et al., 2019). However, when TAK242 was tested in a clinical trial, it failed to reduce serum cytokine levels in patients with severe sepsis and shock or respiratory failure (Rice et al., 2010). Finally, CU-CPT22 (another selective inhibitor for TLR1/2) also reduced TNFα production in primary microglia cells treated with oligomeric α-synuclein and blocked NF-κB nuclear translocation (Daniele et al., 2015). Although most of the existing therapeutic approaches target PD, one of the most promising strategies against MSA is the use of sertraline, a second-generation selective serotonin reuptake inhibitor (SSRI). Sertraline inhibits dynamin 1 and 2 thus blocking the endocytic pathway and it has been shown to inhibit α-synuclein uptake by oligodendrocytes and to prevent pathological α-synuclein spread (Konno et al., 2012). Another SSRI, paroxetine, has already been clinically tested at doses of 30 mg for 2 weeks in 20 patients with MSA and resulted into statistically significant motor improvement (Friess et al., 2006).

## Immunotherapy

**FIGURE 4:**
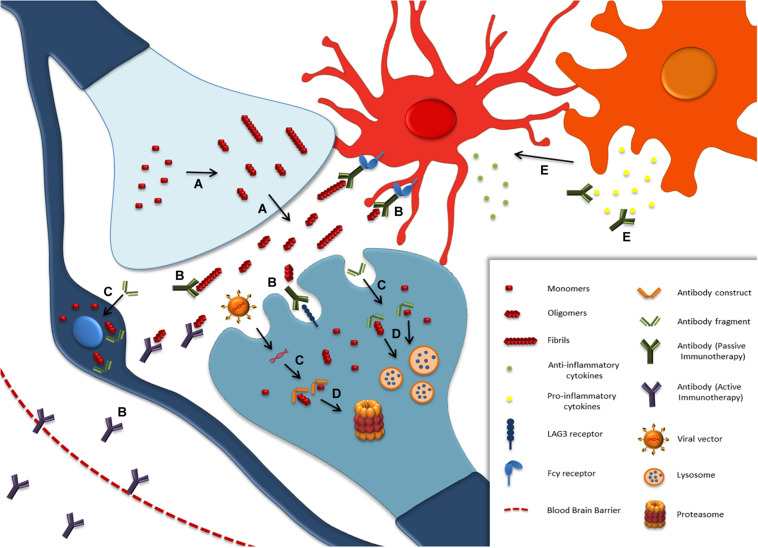
Immunotherapy approaches against α-synuclein. **(A)** Thermodynamically unstable monomers of α-synuclein misfold and aggregate into pathological species. The release of oligomeric and fibrillar α-synuclein into the extracellular space triggers α-synuclein propagation into non-affected cells, microglial activation and neuroinflammation. **(B)** Active or passive immunization mainly aims to lower the extracellular α-synuclein by microglial-mediated degradation and to prevent pathology propagation via antibody binding on receptors facilitating α-synuclein endocytosis. **(C)** The effective targeting of intracellular α-synuclein is achieved by antibody fragments paired with signaling peptides for endocytosis, or viral vector-derived antibody constructs, which are expressed within the cells. **(D)** The engineered intrabodies bind to α-synuclein toxic species and lead them to degradation through proteasomal or autophagic pathways. **(E)** Antibodies against immune system activation is another immunotherapy approach, which aims to reduce pro-inflammatory cytokine release, enhance anti-inflammatory microglial activity and, by that, prevent α-synuclein pathology progression.

The role of the immune system and neuroinflammation in the pathophysiology of PD and related synucleinopathies, along with the specificity of antigen-antibody binding, render immunotherapy (active and passive) one of the most promising therapeutic approaches (Figure 4). The first study of active immunization against α-synuclein utilized full-length α-synuclein vaccination in the PDGF transgenic α-synuclein mice and resulted in decreased α-synuclein accumulation in neuronal cell bodies and synapses (Masliah et al., 2005). Active vaccination of the PDGF and the Thy1 transgenic α-synuclein mice with the PD01A and PD03A vaccines (also known as AFFITOPE) reduced α-synuclein oligomers in axons and synapses, decreased caudo-putamen nucleus degeneration and memory deficits in both mouse models (Mandler et al., 2014). The same results, along with reduced demyelination were observed in an MSA transgenic mouse model (Mandler et al., 2015). Recently, the AFFITOPE was tested and successfully passed into the Phase 1 clinical trial in MSA patients, whereas the clinical trial for PD patients is ongoing^2^. More recently, it was shown that a novel vaccination modality combining an antigen-presenting cell-targeting glucan particle (GP) vaccine delivery system with encapsulated antigen (α-synuclein) plus rapamycin induced both strong anti-α-synuclein antibody titers and neuroprotective T regulatory (Tregs) responses in synucleinopathy models, being more effective than the humoral or cellular immunization alone (Rockenstein et al., 2018). In the same concept, prophylactic vaccinations with full-length recombinant α-synuclein in rats, which subsequent receive AAV-α-synuclein, prevented the accumulation of α-synuclein through the induction of Tregs and microglia activation (Christiansen et al., 2016).

Passive immunization with monoclonal antibodies targeting mostly the C- and N-terminal region of α-synuclein have been also used as means to sequester the extracellular protein, thus inhibiting the propagation of the disease (Masliah et al., 2011; Games et al., 2014; Tran et al., 2014; Shahaduzzaman et al., 2015). The first passive immunotherapy using the PRX002 or 9E4 antibody was tested in 2011, in the PDGF-α-synuclein transgenic mice, and resulted in reduced α-synuclein accumulation in axons and synapses, enhanced lysosomal degradation and improved behavioral and motor defects (Masliah et al., 2011). The promising preclinical results of two antibodies, the PRX002 and the BIIB054 (Weihofen et al., 2019), led to their testing in clinical trials where both of them were found safe and effective in preventing α-synuclein spread. Specifically, PRX002 (PRX002, initially developed by Perrigo, Allegan, MI, United States; patent number US7910333; NCT02157714 and NCT02095171) is a humanized IgG1 monoclonal antibody that resulted in 96.5% decrease of free serum α-synuclein in phase 1a clinical trial (Schenk et al., 2017) and 97% decrease following a single intravenous infusion of the highest dose (60 mg/kg) in phase 1b and these findings prompted the design of an ongoing phase 2 clinical trial (NCT03100149) (Jankovic et al., 2018). BIIB054 (licensed by Biogen) is a fully human-derived monoclonal antibody specifically raised against aggregated and fibrillar α-synuclein that displayed beneficial effects regarding α-synuclein aggregation in the PFF-inoculated PD mouse model (Weihofen et al., 2019). The results from phase 1 clinical trial in PD and healthy patients revealed that BIIB054 is safe and tolerable and have allowed the initiation of phase 2 trial (NCT03318523) (Brys et al., 2019). Finally, other monoclonal antibodies raised against misfolded α-synuclein (Syn211 and Syn303) have been efficiently used *in vivo* and inhibited α-synuclein uptake and pathology transmission (Tran et al., 2014). Other C-terminal targeting α-synuclein antibodies, such as 1H7, 5C1, A1-A6 and ab274 antibodies, were efficiently shown to decrease protein aggregation and exert neuroprotective effects (Bae et al., 2012; Games et al., 2014; Sahin et al., 2017).

The first reported N-terminal targeting antibody, AB1, prevented dopaminergic neuron loss and microgliosis in an AAV-α-synuclein rat model of PD (Shahaduzzaman et al., 2015). Subsequently, the aggregate-selective BIIB054 human α-synuclein antibody was reported to attenuate the spreading of α-synuclein pathology, to rescue motor impairments and to reduce the loss of dopamine transporter density in the nigrostriatal terminals in three different PFF-inoculated mouse models (Weihofen et al., 2019). BIIB054 was originally licensed by Biogen in 2010 and it was first tested in healthy volunteers in 2015. Since then, BIIB054 successfully passed through Phase 1 clinical testing for safety and tolerability and a Phase 2 clinical trial in 311 PD patients is ongoing^3^ (Brys et al., 2019). The aggregated forms of α-synuclein are also the target of the monoclonal antibodies Syn211 and Syn303, with promising reported results in cellular and animal α-synuclein PFF-models (Tran et al., 2014). Antibody targeting of the NAC region is another approach. As such, administration of the NAC32 antibody decreased formation of aberrant species and mitigated α-synuclein-related toxicity by 75% in an A53T mutant α-synuclein neuronal cell line. Importantly, the NAC32 established the prime basis of intrabodies manufacture, and open up new opportunities for intracellular α-synuclein targeting (Lynch et al., 2008). Direct targeting of oligomers is another way to inhibit further the aggregation process and promote the clearance of pathological α-synuclein assemblies. *In vitro* [D5 antibody, (Emadi et al., 2007)] and *in vivo* [Syn-10H antibody, (Emadi et al., 2009)] studies using such oligomer-specific antibodies reported promising anti-aggregation effects. In addition, the D5 antibody paired with the LDL receptor-binding domain of apolipoprotein B resulted in increased penetration of the construct intrabody through the endosomal sorting complex required for transport (ESCRT) and resulted in enhanced α-synuclein oligomer degradation *in vivo* (Spencer et al., 2014). Based on this, the combined administration of a CD5-D5 single-chain antibody prevented astrogliosis, microgliosis and α-synuclein aggregation in a mouse model of MSA (Valera et al., 2017).

Likewise, in the PLP-α-synuclein MSA mouse model, administration of the Rec47 antibody led to enhanced α-synuclein clearance, reduced intracellular seeding of the protein and limited microglial activation (Kallab et al., 2018). Furthermore, the oligomer-specific mAb47 antibody successfully prevented α-synuclein assembly formation in murine co-cultures of astrocytes, oligodendrocytes and neurons and, interestingly, protected astroglia from an oligomeric α-synuclein-mediated mitochondrial dysfunction (Gustafsson et al., 2017). Moreover, MEDI1341, a high affinity α-synuclein antibody that crosses the BBB and binds monomeric and aggregated α-synuclein, prevented cell-to-cell transmission of α-synuclein in PFF-treated neuronal cells and is currently tested in a Phase 1 clinical trial for α-synucleinopathies (ClinicalTrials.gov identifier NCT03272165) (Schofield et al., 2019). A phase 1 clinical study with the monoclonal antibody ABBV-0805 (or BAN0805) that selectively targets oligomeric and protofibrilar forms of α-synuclein has initiated, however no results have been published yet. A variety of antibodies against oligomeric and fibrillar forms of α-synuclein were recently reported. Syn-O1, Syn-O2 and Syn-O4 target oligomers and Syn-F1 and Syn-F2 recognize the fibrillar assemblies. Syn-O1, Syn-O4 and Syn–F1 antibodies limited α-synuclein aggregation with higher efficacy than the other constructs when tested in the PDGF α-synuclein mouse model (El-Agnaf et al., 2017).

Finally, many current immunotherapeutic approaches aim to modulate the immune system imbalance evoked by misfolded α-synuclein accumulation by promoting anti-inflammatory and neuroprotective conditions (Reynolds et al., 2010). Therapies, including administration of the granulocyte macrophage-colony stimulating factor (GM-CSF) and the vasoactive intestinal peptide (VIP), avert the inflammatory role of effector T cells (Teff) by inducing T regulatory cells (Tregs), which are severely diminished in PD patients (Saunders et al., 2012). The GM-CSF stimulating factor, sargramostim, is already in clinical trial phase 1 for PD providing promising results in motor defects improvement (Gendelman et al., 2017). Unfortunately, VIP is rapidly metabolized; hence a VIPR2 agonist, LBT3627, has been designed and used in α-synuclein overexpression rats and MPTP-intoxicated mice with beneficial results (Olson et al., 2015, 2016; Mosley et al., 2019). Therefore, its promising outcome in animal models renders VIP agonist as a therapeutic candidate for clinical testing in the future.

## Targeting the Gut

α-synuclein spread via the gut-brain axis has gained attention in the last years as a potential therapeutic target against α-synucleinopathies (Holmqvist et al., 2014; Lee et al., 2018; Manfredsson et al., 2018). Many studies have focused on gut microbiota as a regulator of α-synuclein misfolding and transmission towards the brain via the vagus nerve (Sampson et al., 2016; Bhattacharyya et al., 2019; Chiang and Lin, 2019) and results have revealed differences in gut microbiota between PD patients and healthy controls suggesting a role of the gut microbiome in PD pathogenesis (Scheperjans et al., 2015; Unger et al., 2016; Lin A. et al., 2018). To this end, the generation of a germfree α-synuclein PD mouse model resulted in decreased α-synuclein toxicity and neuroinflammation; however when the same mice received gut microbiota from PD patients, they exhibited motor impairment (Sampson et al., 2016). Gastrointestinal dysfunction is connected to PD and several approaches have been developed to alleviate α-synuclein pathological effects, such as therapeutic strategies to stimulate gastric motility (Moore et al., 2018), use of antibiotics and microbiota replacement (Fasano et al., 2015; Felice et al., 2016).

## Concluding Remarks and Future Perspectives

Undoubtedly, α-synuclein plays a crucial role in the initiation and progression of the neurodegenerative cascade characterizing PD and related synucleinopathies. Candidate attractive options for therapy aim to reduce protein production or enhance protein degradation, to inhibit protein aggregation and misfolding, to alter α-synuclein post-translational modifications or to target the extracellular levels of the protein with antibodies, thus intervening in the protein uptake and cell-cell propagation mechanisms. All approaches should be handled with caution, since uncontrolled manipulation of the global α-synuclein levels may lead to neurotoxicity, due to the prevailing role of the protein in synaptic neurotransmission. Regarding the degradation strategies, experimental findings surmise that such strategies imply extensive knowledge about dosage and timing of application, a fact that may limit their current therapeutic applicability. Others and we have found that under specific circumstances induction of macroautophagy can have detrimental effects, thus the therapeutic utility of chemical modulators of macroautophagy or even CMA should be examined with caution given that they may be involved in diverse range of processes. Our experience in regards to CMA enhancement showed that this approach represents a fruitful strategy for synucleinopathies, at least in rodent cellular and animal models. This therapeutic modality is currently being tested in a non-human primate model of α-synucleinopathy and if proven successful it may pave the way for its possible clinical utility for the treatment of PD and related synucleinopathies. In regards to the approaches targeting extracellular α-synuclein, they seem to represent a compelling strategy to slow or halt disease progression, by interfering to the cell-cell transmission mechanisms. However, it still remains to be elucidated whether this transmission involves only the neuronal connectome and, most importantly, which species or strains of the protein are the main culprits for the pathology transmission.

In addition, a growing body of evidence suggests that an underlying cause of the heterogeneity characterizing synucleinopathies is the presence of distinct α-synuclein strains in patient samples. As such, PD- and MSA- derived patient α-synuclein strains exhibit different biophysical/biochemical properties and evoke different responses in cultured cells and animal models. Furthermore, the cellular milieu seems to affect the pathogenetic properties of the engendered strains, suggesting that other co-factors may alter disease initiation and progression. Hence, the aforementioned therapeutic strategies targeting the degradation, modification, secretion and seeding of the distinct strains may result in different outcomes in neurons and oligodendrocytes, raising the possibility of the application of a precision medicine in synucleinopathies in the near future. By utilizing advanced structural biology techniques and cryo-electron microscopy we may attain a better understanding of the clinical heterogeneity amongst α-synucleinopathies, thus offering the opportunity for the future development of strain-specific therapies to halt or delay disease progression.

Collectively, we believe that further research is needed to better understand the precise mechanisms underlying the α-synuclein pathology enigma and to pinpoint the factors that differentiate the pathology observed in synucleinopathies.

## Author Contributions

MF, PM, and GT wrote the initial draft. MX revised and generated the final version of the manuscript. All authors contributed to the article and approved the submitted version.

## Conflict of Interest

The authors declare that the research was conducted in the absence of any commercial or financial relationships that could be construed as a potential conflict of interest.

## Footnotes

1 https://lti-staging.squarespace.com/our-science/#lti-291

2 http://sympath-project.eu/wp-content/uploads/PR_AFF009_V1.pdf

3 https://www.alzforum.org/therapeutics/biib054

## References

[B1] Aarsland D. Laake K. Larsen J. P. Janvin C. (2002). Donepezil for cognitive impairment in Parkinson’s disease: a randomised controlled study. *J. Neurol. Neurosurg. Psychiatry* 72 708–712. 10.1136/jnnp.72.6.708 12023410 PMC1737925

[B2] Abdelmotilib H. Maltbie T. Delic V. Liu Z. Hu X. Fraser K. B. (2017). alpha-Synuclein fibril-induced inclusion spread in rats and mice correlates with dopaminergic Neurodegeneration. *Neurobiol. Dis.* 105 84–98. 10.1016/j.nbd.2017.05.014 28576704 PMC5701756

[B3] Abounit S. Bousset L. Loria F. Zhu S. de Chaumont F. Pieri L. (2016). Tunneling nanotubes spread fibrillar alpha-synuclein by intercellular trafficking of lysosomes. *EMBO J.* 35 2120–2138. 10.15252/embj.201593411 27550960 PMC5048354

[B4] Afitska K. Fucikova A. Shvadchak V. V. Yushchenko D. A. (2019). alpha-Synuclein aggregation at low concentrations. *Biochim. Biophys. Acta Proteins Proteom.* 1867 701–709. 10.1016/j.bbapap.2019.05.003 31096048

[B5] Ahn K. J. Paik S. R. Chung K. C. Kim J. (2006). Amino acid sequence motifs and mechanistic features of the membrane translocation of alpha-synuclein. *J. Neurochem.* 97 265–279. 10.1111/j.1471-4159.2006.03731.x 16524375

[B6] Alarcon-Aris D. Recasens A. Galofre M. Carballo-Carbajal I. Zacchi N. Ruiz-Bronchal E. (2018). Selective alpha-synuclein knockdown in monoamine neurons by intranasal oligonucleotide delivery: potential therapy for Parkinson’s disease. *Mol. Ther.* 26 550–567. 10.1016/j.ymthe.2017.11.015 29273501 PMC5835151

[B7] Alvarez-Erviti L. Rodriguez-Oroz M. C. Cooper J. M. Caballero C. Ferrer I. Obeso J. A. (2010). Chaperone-mediated autophagy markers in Parkinson disease brains. *Arch. Neurol.* 67 1464–1472. 10.1001/archneurol.2010.198 20697033

[B8] Alvarez-Erviti L. Seow Y. Schapira A. H. Gardiner C. Sargent I. L. Wood M. J. (2011). Lysosomal dysfunction increases exosome-mediated alpha-synuclein release and transmission. *Neurobiol. Dis.* 42 360–367. 10.1016/j.nbd.2011.01.029 21303699 PMC3107939

[B9] Ambrosi G. Ghezzi C. Zangaglia R. Levandis G. Pacchetti C. Blandini F. (2015). Ambroxol-induced rescue of defective glucocerebrosidase is associated with increased LIMP-2 and saposin C levels in GBA1 mutant Parkinson’s disease cells. *Neurobiol. Dis.* 82 235–242. 10.1016/j.nbd.2015.06.008 26094596

[B10] Anderson A. C. Joller N. Kuchroo V. K. (2016). Lag-3, Tim-3, and TIGIT: co-inhibitory receptors with specialized functions in immune regulation. *Immunity* 44 989–1004. 10.1016/j.immuni.2016.05.001 27192565 PMC4942846

[B11] Anglade P. Vyas S. Javoy-Agid F. Herrero M. T. Michel P. P. Marquez J. (1997). Apoptosis and autophagy in nigral neurons of patients with Parkinson’s disease. *Histol. Histopathol.* 12 25–31. 9046040

[B12] Angot E. Steiner J. A. Lema Tome C. M. Ekstrom P. Mattsson B. Bjorklund A. (2012). Alpha-synuclein cell-to-cell transfer and seeding in grafted dopaminergic neurons *in vivo*. *PLoS One* 7:e39465. 10.1371/journal.pone.0039465 22737239 PMC3380846

[B13] Arias E. Koga H. Diaz A. Mocholi E. Patel B. Cuervo A. M. (2015). Lysosomal mTORC2/PHLPP1/Akt regulate chaperone-mediated autophagy. *Mol. Cell* 59 270–284. 10.1016/j.molcel.2015.05.030 26118642 PMC4506737

[B14] Arotcarena M. L. Bourdenx M. Dutheil N. Thiolat M. L. Doudnikoff E. Dovero S. (2019). Transcription factor EB overexpression prevents neurodegeneration in experimental synucleinopathies. *JCI Insight* 4:e129719. 10.1172/jci.insight.129719 31434803 PMC6777809

[B15] Asi Y. T. Simpson J. E. Heath P. R. Wharton S. B. Lees A. J. Revesz T. (2014). Alpha-synuclein mRNA expression in oligodendrocytes in MSA. *Glia* 62 964–970. 10.1002/glia.22653 24590631 PMC4238782

[B16] Aulic S. Masperone L. Narkiewicz J. Isopi E. Bistaffa E. Ambrosetti E. (2017). alpha-Synuclein Amyloids Hijack Prion Protein to Gain Cell Entry, Facilitate Cell-to-Cell Spreading and Block Prion Replication. *Sci. Rep.* 7:10050. 10.1038/s41598-017-10236-x 28855681 PMC5577263

[B17] Badiola N. de Oliveira R. M. Herrera F. Guardia-Laguarta C. Goncalves S. A. Pera M. (2011). Tau enhances alpha-synuclein aggregation and toxicity in cellular models of synucleinopathy. *PLoS One* 6:e26609. 10.1371/journal.pone.0026609 22039514 PMC3200341

[B18] Bae E. J. Lee H. J. Rockenstein E. Ho D. H. Park E. B. Yang N. Y. (2012). Antibody-aided clearance of extracellular alpha-synuclein prevents cell-to-cell aggregate transmission. *J. Neurosci.* 32 13454–13469. 10.1523/JNEUROSCI.1292-12.2012 23015436 PMC3752153

[B19] Bai X. Wey M. C. Fernandez E. Hart M. J. Gelfond J. Bokov A. F. (2015). Rapamycin improves motor function, reduces 4-hydroxynonenal adducted protein in brain, and attenuates synaptic injury in a mouse model of synucleinopathy. *Pathobiol. Aging Age Relat. Dis.* 5:28743. 10.3402/pba.v5.28743 26306821 PMC4549373

[B20] Barkholt P. Sanchez-Guajardo V. Kirik D. Romero-Ramos M. (2012). Long-term polarization of microglia upon alpha-synuclein overexpression in nonhuman primates. *Neuroscience* 208 85–96. 10.1016/j.neuroscience.2012.02.004 22342967

[B21] Barrett P. J. Timothy Greenamyre J. (2015). Post-translational modification of alpha-synuclein in Parkinson’s disease. *Brain Res.* 1628(Pt B), 247–253. 10.1016/j.brainres.2015.06.002 26080075

[B22] Bartels T. Choi J. G. Selkoe D. J. (2011). alpha-Synuclein occurs physiologically as a helically folded tetramer that resists aggregation. *Nature* 477 107–110. 10.1038/nature10324 21841800 PMC3166366

[B23] Bennett M. C. Bishop J. F. Leng Y. Chock P. B. Chase T. N. Mouradian M. M. (1999). Degradation of alpha-synuclein by proteasome. *J. Biol. Chem.* 274 33855–33858. 10.1074/jbc.274.48.33855 10567343

[B24] Benskey M. J. Sellnow R. C. Sandoval I. M. Sortwell C. E. Lipton J. W. Manfredsson F. P. (2018). Silencing alpha synuclein in mature Nigral neurons results in rapid neuroinflammation and subsequent toxicity. *Front. Mol. Neurosci.* 11:36. 10.3389/fnmol.2018.00036 29497361 PMC5819572

[B25] Bentea E. Verbruggen L. Massie A. (2017). The proteasome inhibition model of Parkinson’s disease. *J. Parkinsons Dis.* 7 31–63. 10.3233/JPD-160921 27802243 PMC5302045

[B26] Bernis M. E. Babila J. T. Breid S. Wusten K. A. Wullner U. Tamguney G. (2015). Prion-like propagation of human brain-derived alpha-synuclein in transgenic mice expressing human wild-type alpha-synuclein. *Acta Neuropathol. Commun.* 3:75. 10.1186/s40478-015-0254-7 26612754 PMC4660655

[B27] Bhatt M. A. Messer A. Kordower J. H. (2013). Can intrabodies serve as neuroprotective therapies for Parkinson’s disease? Beginning thoughts. *J. Parkinsons Dis.* 3 581–591. 10.3233/JPD-130252 24270241

[B28] Bhattacharyya D. Mohite G. M. Krishnamoorthy J. Gayen N. Mehra S. Navalkar A. (2019). Lipopolysaccharide from gut microbiota modulates alpha-synuclein aggregation and alters its biological function. *ACS Chem. Neurosci.* 10 2229–2236. 10.1021/acschemneuro.8b00733 30855940

[B29] Bieschke J. Russ J. Friedrich R. P. Ehrnhoefer D. E. Wobst H. Neugebauer K. (2010). EGCG remodels mature alpha-synuclein and amyloid-beta fibrils and reduces cellular toxicity. *Proc. Natl. Acad. Sci. U.S.A.* 107 7710–7715. 10.1073/pnas.0910723107 20385841 PMC2867908

[B30] Birol M. Wojcik S. P. Miranker A. D. Rhoades E. (2019). Identification of N-linked glycans as specific mediators of neuronal uptake of acetylated alpha-Synuclein. *PLoS Biol.* 17:e3000318. 10.1371/journal.pbio.3000318 31211781 PMC6599126

[B31] Blanz J. Saftig P. (2016). Parkinson’s disease: acid-glucocerebrosidase activity and alpha-synuclein clearance. *J. Neurochem.* 139(Suppl. 1), 198–215. 10.1111/jnc.13517 26860955

[B32] Bose A. Beal M. F. (2016). Mitochondrial dysfunction in Parkinson’s disease. *J. Neurochem.* 139(Suppl. 1), 216–231. 10.1111/jnc.13731 27546335

[B33] Bousset L. Pieri L. Ruiz-Arlandis G. Gath J. Jensen P. H. Habenstein B. (2013). Structural and functional characterization of two alpha-synuclein strains. *Nat. Commun.* 4:2575. 10.1038/ncomms3575 24108358 PMC3826637

[B34] Braak H. de Vos R. A. Bohl J. Del Tredici K. (2006). Gastric alpha-synuclein immunoreactive inclusions in Meissner’s and Auerbach’s plexuses in cases staged for Parkinson’s disease-related brain pathology. *Neurosci. Lett.* 396 67–72. 10.1016/j.neulet.2005.11.012 16330147

[B35] Braak H. Del Tredici K. Rub U. de Vos R. A. Jansen Steur E. N. Braak E. (2003). Staging of brain pathology related to sporadic Parkinson’s disease. *Neurobiol. Aging* 24 197–211. 10.1016/s0197-4580(02)00065-9 12498954

[B36] Brahmachari S. Ge P. Lee S. H. Kim D. Karuppagounder S. S. Kumar M. (2016). Activation of tyrosine kinase c-Abl contributes to alpha-synuclein-induced neurodegeneration. *J. Clin. Invest.* 126 2970–2988. 10.1172/JCI85456 27348587 PMC4966315

[B37] Brenner S. Wersinger C. Gasser T. (2015). Transcriptional regulation of the alpha-synuclein gene in human brain tissue. *Neurosci. Lett.* 599 140–145. 10.1016/j.neulet.2015.05.029 26002080

[B38] Bruening W. Giasson B. I. Klein-Szanto A. J. Lee V. M. Trojanowski J. Q. Godwin A. K. (2000). Synucleins are expressed in the majority of breast and ovarian carcinomas and in preneoplastic lesions of the ovary. *Cancer* 88 2154–2163. 10.1002/(sici)1097-0142(20000501)88:9<2154::aid-cncr23>3.0.co;2-9 10813729

[B39] Brys M. Fanning L. Hung S. Ellenbogen A. Penner N. Yang M. (2019). Randomized phase I clinical trial of anti-alpha-synuclein antibody BIIB054. *Mov. Disord.* 34 1154–1163. 10.1002/mds.27738 31211448 PMC6771554

[B40] Burn D. Emre M. McKeith I. De Deyn P. P. Aarsland D. Hsu C. (2006). Effects of rivastigmine in patients with and without visual hallucinations in dementia associated with Parkinson’s disease. *Mov. Disord.* 21 1899–1907. 10.1002/mds.21077 16960863

[B41] Burre J. Sharma M. Sudhof T. C. (2014). alpha-Synuclein assembles into higher-order multimers upon membrane binding to promote SNARE complex formation. *Proc. Natl. Acad. Sci. U.S.A.* 111 E4274–E4283. 10.1073/pnas.1416598111 25246573 PMC4210039

[B42] Burre J. Sharma M. Tsetsenis T. Buchman V. Etherton M. R. Sudhof T. C. (2010). Alpha-synuclein promotes SNARE-complex assembly *in vivo* and *in vitro*. *Science* 329 1663–1667. 10.1126/science.1195227 20798282 PMC3235365

[B43] Busch D. J. Oliphint P. A. Walsh R. B. Banks S. M. Woods W. S. George J. M. (2014). Acute increase of alpha-synuclein inhibits synaptic vesicle recycling evoked during intense stimulation. *Mol. Biol. Cell* 25 3926–3941. 10.1091/mbc.E14-02-0708 25273557 PMC4244201

[B44] Bussell R. Jr. Eliezer D. (2001). Residual structure and dynamics in Parkinson’s disease-associated mutants of alpha-synuclein. *J. Biol. Chem.* 276 45996–46003. 10.1074/jbc.M106777200 11590151

[B45] Butler B. Saha K. Rana T. Becker J. P. Sambo D. Davari P. (2015). Dopamine transporter activity is modulated by alpha-Synuclein. *J. Biol. Chem.* 290 29542–29554. 10.1074/jbc.M115.691592 26442590 PMC4705954

[B46] Butler B. Sambo D. Khoshbouei H. (2017). Alpha-synuclein modulates dopamine neurotransmission. *J. Chem. Neuroanat.* 83-84 41–49. 10.1016/j.jchemneu.2016.06.001 27334403 PMC5167661

[B47] Candelise N. Schmitz M. Llorens F. Villar-Pique A. Cramm M. Thom T. (2019). Seeding variability of different alpha synuclein strains in synucleinopathies. *Ann. Neurol.* 85 691–703. 10.1002/ana.25446 30805957

[B48] Casarejos M. J. Solano R. M. Gomez A. Perucho J. de Yebenes J. G. Mena M. A. (2011). The accumulation of neurotoxic proteins, induced by proteasome inhibition, is reverted by trehalose, an enhancer of autophagy, in human neuroblastoma cells. *Neurochem. Int.* 58 512–520. 10.1016/j.neuint.2011.01.008 21232572

[B49] Chandra S. Chen X. Rizo J. Jahn R. Sudhof T. C. (2003). A broken alpha -helix in folded alpha -Synuclein. *J. Biol. Chem.* 278 15313–15318. 10.1074/jbc.M213128200 12586824

[B50] Chandra S. Gallardo G. Fernandez-Chacon R. Schluter O. M. Sudhof T. C. (2005). Alpha-synuclein cooperates with CSPalpha in preventing neurodegeneration. *Cell* 123 383–396. 10.1016/j.cell.2005.09.028 16269331

[B51] Charles V. Mezey E. Reddy P. H. Dehejia A. Young T. A. Polymeropoulos M. H. (2000). Alpha-synuclein immunoreactivity of huntingtin polyglutamine aggregates in striatum and cortex of Huntington’s disease patients and transgenic mouse models. *Neurosci. Lett.* 289 29–32. 10.1016/s0304-3940(00)01247-7 10899401

[B52] Chartier-Harlin M. C. Kachergus J. Roumier C. Mouroux V. Douay X. Lincoln S. (2004). Alpha-synuclein locus duplication as a cause of familial Parkinson’s disease. *Lancet* 364 1167–1169. 10.1016/S0140-6736(04)17103-1 15451224

[B53] Chatterjee D. Bhatt M. Butler D. De Genst E. Dobson C. M. Messer A. (2018). Proteasome-targeted nanobodies alleviate pathology and functional decline in an alpha-synuclein-based Parkinson’s disease model. *NPJ Parkinsons Dis.* 4:25. 10.1038/s41531-018-0062-4 30155513 PMC6105584

[B54] Chau K. Y. Ching H. L. Schapira A. H. Cooper J. M. (2009). Relationship between alpha synuclein phosphorylation, proteasomal inhibition and cell death: relevance to Parkinson’s disease pathogenesis. *J. Neurochem.* 110 1005–1013. 10.1111/j.1471-4159.2009.06191.x 19493164

[B55] Chen H. Chan D. C. (2009). Mitochondrial dynamics–fusion, fission, movement, and mitophagy–in neurodegenerative diseases. *Hum. Mol. Genet.* 18 R169–R176. 10.1093/hmg/ddp326 19808793 PMC2758711

[B56] Chen J. Ren Y. Gui C. Zhao M. Wu X. Mao K. (2018). Phosphorylation of Parkin at serine 131 by p38 MAPK promotes mitochondrial dysfunction and neuronal death in mutant A53T alpha-synuclein model of Parkinson’s disease. *Cell Death Dis.* 9:700. 10.1038/s41419-018-0722-7 29899409 PMC5999948

[B57] Chen L. Feany M. B. (2005). Alpha-synuclein phosphorylation controls neurotoxicity and inclusion formation in a Drosophila model of Parkinson disease. *Nat. Neurosci.* 8 657–663. 10.1038/nn1443 15834418

[B58] Chen M. Yang W. Li X. Wang P. Yue F. Yang H. (2016). Age- and brain region-dependent alpha-synuclein oligomerization is attributed to alterations in intrinsic enzymes regulating alpha-synuclein phosphorylation in aging monkey brains. *Oncotarget* 7 8466–8480. 10.18632/oncotarget.6445 27032368 PMC4890980

[B59] Chen Q. Q. Haikal C. Li W. Li J. Y. (2019). Gut inflammation in association with pathogenesis of Parkinson’s disease. *Front. Mol. Neurosci.* 12:218. 10.3389/fnmol.2019.00218 31572126 PMC6753187

[B60] Chen S. G. Stribinskis V. Rane M. J. Demuth D. R. Gozal E. Roberts A. M. (2016). Exposure to the functional bacterial amyloid protein Curli enhances Alpha-synuclein aggregation in aged Fischer 344 Rats and *Caenorhabditis elegans*. *Sci. Rep.* 6:34477. 10.1038/srep34477 27708338 PMC5052651

[B61] Chen Y. Wei Q. Q. Ou R. Cao B. Chen X. Zhao B. (2015). Genetic Variants of SNCA are associated with susceptibility to Parkinson’s disease but not amyotrophic lateral sclerosis or multiple system atrophy in a Chinese population. *PLoS One* 10:e0133776. 10.1371/journal.pone.0133776 26208350 PMC4514852

[B62] Chiang H. L. Lin C. H. (2019). Altered gut microbiome and intestinal pathology in Parkinson’s disease. *J. Mov. Disord.* 12 67–83. 10.14802/jmd.18067 31158941 PMC6547039

[B63] Chiba Y. Takei S. Kawamura N. Kawaguchi Y. Sasaki K. Hasegawa-Ishii S. (2012). Immunohistochemical localization of aggresomal proteins in glial cytoplasmic inclusions in multiple system atrophy. *Neuropathol. Appl. Neurobiol.* 38 559–571. 10.1111/j.1365-2990.2011.01229.x 22013984

[B64] Choi B. K. Choi M. G. Kim J. Y. Yang Y. Lai Y. Kweon D. H. (2013). Large alpha-synuclein oligomers inhibit neuronal SNARE-mediated vesicle docking. *Proc. Natl. Acad. Sci. U.S.A.* 110 4087–4092. 10.1073/pnas.1218424110 23431141 PMC3593925

[B65] Choi D. C. Yoo M. Kabaria S. Junn E. (2018). MicroRNA-7 facilitates the degradation of alpha-synuclein and its aggregates by promoting autophagy. *Neurosci. Lett.* 678 118–123. 10.1016/j.neulet.2018.05.009 29738845 PMC5990033

[B66] Choi J. G. Kim N. Ju I. G. Eo H. Lim S. M. Jang S. E. (2018). Oral administration of *Proteus mirabilis* damages dopaminergic neurons and motor functions in mice. *Sci. Rep.* 8:1275. 10.1038/s41598-018-19646-x 29352191 PMC5775305

[B67] Choi K. C. Kim S. H. Ha J. Y. Kim S. T. Son J. H. (2010). A novel mTOR activating protein protects dopamine neurons against oxidative stress by repressing autophagy related cell death. *J. Neurochem.* 112 366–376. 10.1111/j.1471-4159.2009.06463.x 19878437

[B68] Choi Y. R. Cha S. H. Kang S. J. Kim J. B. Jou I. Park S. M. (2018). Prion-like propagation of alpha-synuclein is regulated by the FcgammaRIIB-SHP-1/2 signaling pathway in neurons. *Cell Rep.* 22 136–148. 10.1016/j.celrep.2017.12.009 29298416

[B69] Choubey V. Safiulina D. Vaarmann A. Cagalinec M. Wareski P. Kuum M. (2011). Mutant A53T alpha-synuclein induces neuronal death by increasing mitochondrial autophagy. *J. Biol. Chem.* 286 10814–10824. 10.1074/jbc.M110.132514 21252228 PMC3060532

[B70] Christiansen J. R. Olesen M. N. Otzen D. E. Romero-Ramos M. Sanchez-Guajardo V. (2016). alpha-Synuclein vaccination modulates regulatory T cell activation and microglia in the absence of brain pathology. *J. Neuroinflammation* 13:74. 10.1186/s12974-016-0532-8 27055651 PMC4825077

[B71] Clark L. N. Kartsaklis L. A. Wolf Gilbert R. Dorado B. Ross B. M. Kisselev S. (2009). Association of glucocerebrosidase mutations with dementia with lewy bodies. *Arch. Neurol.* 66 578–583. 10.1001/archneurol.2009.54 19433657 PMC2758782

[B72] Clough R. L. Dermentzaki G. Stefanis L. (2009). Functional dissection of the alpha-synuclein promoter: transcriptional regulation by ZSCAN21 and ZNF219. *J. Neurochem.* 110 1479–1490. 10.1111/j.1471-4159.2009.06250.x 19549071

[B73] Clough R. L. Stefanis L. (2007). A novel pathway for transcriptional regulation of alpha-synuclein. *FASEB J.* 21 596–607. 10.1096/fj.06-7111com 17167067

[B74] Colla E. (2019). Linking the endoplasmic reticulum to Parkinson’s disease and Alpha-Synucleinopathy. *Front. Neurosci.* 13:560. 10.3389/fnins.2019.00560 31191239 PMC6550095

[B75] Colla E. Coune P. Liu Y. Pletnikova O. Troncoso J. C. Iwatsubo T. (2012). Endoplasmic reticulum stress is important for the manifestations of alpha-synucleinopathy *in vivo*. *J. Neurosci.* 32 3306–3320. 10.1523/JNEUROSCI.5367-11.2012 22399753 PMC3461828

[B76] Collier T. J. Redmond D. E. Jr. Steece-Collier K. Lipton J. W. Manfredsson F. P. (2016). Is Alpha-synuclein loss-of-function a contributor to Parkinsonian pathology? Evidence from Non-human Primates. *Front. Neurosci.* 10:12. 10.3389/fnins.2016.00012 26858591 PMC4731516

[B77] Conway K. A. Harper J. D. Lansbury P. T. (1998). Accelerated *in vitro* fibril formation by a mutant alpha-synuclein linked to early-onset Parkinson disease. *Nat. Med.* 4 1318–1320. 10.1038/3311 9809558

[B78] Conway K. A. Lee S. J. Rochet J. C. Ding T. T. Williamson R. E. Lansbury P. T. Jr. (2000). Acceleration of oligomerization, not fibrillization, is a shared property of both alpha-synuclein mutations linked to early-onset Parkinson’s disease: implications for pathogenesis and therapy. *Proc. Natl. Acad. Sci. U.S.A.* 97 571–576. 10.1073/pnas.97.2.571 10639120 PMC15371

[B79] Conway K. A. Rochet J. C. Bieganski R. M. Lansbury P. T. Jr. (2001). Kinetic stabilization of the alpha-synuclein protofibril by a dopamine-alpha-synuclein adduct. *Science* 294 1346–1349. 10.1126/science.1063522 11701929

[B80] Cooper J. M. Wiklander P. B. Nordin J. Z. Al-Shawi R. Wood M. J. Vithlani M. (2014). Systemic exosomal siRNA delivery reduced alpha-synuclein aggregates in brains of transgenic mice. *Mov. Disord.* 29 1476–1485. 10.1002/mds.25978 25112864 PMC4204174

[B81] Couch Y. Alvarez-Erviti L. Sibson N. R. Wood M. J. Anthony D. C. (2011). The acute inflammatory response to intranigral alpha-synuclein differs significantly from intranigral lipopolysaccharide and is exacerbated by peripheral inflammation. *J. Neuroinflammation* 8:166. 10.1186/1742-2094-8-166 22122884 PMC3239418

[B82] Cremades N. Cohen S. I. Deas E. Abramov A. Y. Chen A. Y. Orte A. (2012). Direct observation of the interconversion of normal and toxic forms of alpha-synuclein. *Cell* 149 1048–1059. 10.1016/j.cell.2012.03.037 22632969 PMC3383996

[B83] Crews L. Spencer B. Desplats P. Patrick C. Paulino A. Rockenstein E. (2010). Selective molecular alterations in the autophagy pathway in patients with Lewy body disease and in models of alpha-synucleinopathy. *PLoS One* 5:e9313. 10.1371/journal.pone.0009313 20174468 PMC2824828

[B84] Cuervo A. M. Stefanis L. Fredenburg R. Lansbury P. T. Sulzer D. (2004). Impaired degradation of mutant alpha-synuclein by chaperone-mediated autophagy. *Science* 305 1292–1295. 10.1126/science.1101738 15333840

[B85] Dalfo E. Barrachina M. Rosa J. L. Ambrosio S. Ferrer I. (2004). Abnormal alpha-synuclein interactions with rab3a and rabphilin in diffuse Lewy body disease. *Neurobiol. Dis.* 16 92–97. 10.1016/j.nbd.2004.01.001 15207266

[B86] Daniele S. G. Beraud D. Davenport C. Cheng K. Yin H. Maguire-Zeiss K. A. (2015). Activation of MyD88-dependent TLR1/2 signaling by misfolded alpha-synuclein, a protein linked to neurodegenerative disorders. *Sci. Signal.* 8:ra45. 10.1126/scisignal.2005965 25969543 PMC4601639

[B87] Danzer K. M. Haasen D. Karow A. R. Moussaud S. Habeck M. Giese A. (2007). Different species of alpha-synuclein oligomers induce calcium influx and seeding. *J. Neurosci.* 27 9220–9232. 10.1523/JNEUROSCI.2617-07.2007 17715357 PMC6672196

[B88] Danzer K. M. Kranich L. R. Ruf W. P. Cagsal-Getkin O. Winslow A. R. Zhu L. (2012). Exosomal cell-to-cell transmission of alpha synuclein oligomers. *Mol. Neurodegener.* 7:42. 10.1186/1750-1326-7-42 22920859 PMC3483256

[B89] Davidson W. S. Jonas A. Clayton D. F. George J. M. (1998). Stabilization of alpha-synuclein secondary structure upon binding to synthetic membranes. *J. Biol. Chem.* 273 9443–9449. 10.1074/jbc.273.16.9443 9545270

[B90] Decressac M. Mattsson B. Weikop P. Lundblad M. Jakobsson J. Bjorklund A. (2013). TFEB-mediated autophagy rescues midbrain dopamine neurons from alpha-synuclein toxicity. *Proc. Natl. Acad. Sci. U.S.A.* 110 E1817–E1826. 10.1073/pnas.1305623110 23610405 PMC3651458

[B91] Deeg A. A. Reiner A. M. Schmidt F. Schueder F. Ryazanov S. Ruf V. C. (2015). Anle138b and related compounds are aggregation specific fluorescence markers and reveal high affinity binding to alpha-synuclein aggregates. *Biochim. Biophys. Acta* 1850 1884–1890. 10.1016/j.bbagen.2015.05.021 26028294

[B92] Dehay B. Bove J. Rodriguez-Muela N. Perier C. Recasens A. Boya P. (2010). Pathogenic lysosomal depletion in Parkinson’s disease. *J. Neurosci.* 30 12535–12544. 10.1523/JNEUROSCI.1920-10.2010 20844148 PMC6633458

[B93] Delenclos M. Trendafilova T. Mahesh D. Baine A. M. Moussaud S. Yan I. K. (2017). Investigation of endocytic pathways for the internalization of exosome-associated oligomeric Alpha-Synuclein. *Front. Neurosci.* 11:172. 10.3389/fnins.2017.00172 28424577 PMC5371652

[B94] Dermentzaki G. Paschalidis N. Politis P. K. Stefanis L. (2016). Complex Effects of the ZSCAN21 transcription factor on transcriptional regulation of alpha-synuclein in primary neuronal cultures and *in vivo*. *J. Biol. Chem.* 291 8756–8772. 10.1074/jbc.M115.704973 26907683 PMC4861444

[B95] Desplats P. Lee H. J. Bae E. J. Patrick C. Rockenstein E. Crews L. (2009). Inclusion formation and neuronal cell death through neuron-to-neuron transmission of alpha-synuclein. *Proc. Natl. Acad. Sci. U.S.A.* 106 13010–13015. 10.1073/pnas.0903691106 19651612 PMC2722313

[B96] Dettmer U. Newman A. J. Soldner F. Luth E. S. Kim N. C. von Saucken V. E. (2015). Parkinson-causing alpha-synuclein missense mutations shift native tetramers to monomers as a mechanism for disease initiation. *Nat. Commun.* 6:7314. 10.1038/ncomms8314 26076669 PMC4490410

[B97] Di Giovanni S. Eleuteri S. Paleologou K. E. Yin G. Zweckstetter M. Carrupt P. A. (2010). Entacapone and tolcapone, two catechol O-methyltransferase inhibitors, block fibril formation of alpha-synuclein and beta-amyloid and protect against amyloid-induced toxicity. *J. Biol. Chem.* 285 14941–14954. 10.1074/jbc.M109.080390 20150427 PMC2865316

[B98] Di Maio R. Hoffman E. K. Rocha E. M. Keeney M. T. Sanders L. H. De Miranda B. R. (2018). LRRK2 activation in idiopathic Parkinson’s disease. *Sci. Transl. Med.* 10:eaar5429. 10.1126/scitranslmed.aar5429 30045977 PMC6344941

[B99] Diao J. Burre J. Vivona S. Cipriano D. J. Sharma M. Kyoung M. (2013). Native alpha-synuclein induces clustering of synaptic-vesicle mimics via binding to phospholipids and synaptobrevin-2/VAMP2. *eLife* 2:e00592. 10.7554/eLife.00592 23638301 PMC3639508

[B100] Diaz E. F. Labra V. C. Alvear T. F. Mellado L. A. Inostroza C. A. Oyarzun J. E. (2019). Connexin 43 hemichannels and pannexin-1 channels contribute to the alpha-synuclein-induced dysfunction and death of astrocytes. *Glia* 67 1598–1619. 10.1002/glia.23631 31033038

[B101] Diepenbroek M. Casadei N. Esmer H. Saido T. C. Takano J. Kahle P. J. (2014). Overexpression of the calpain-specific inhibitor calpastatin reduces human alpha-Synuclein processing, aggregation and synaptic impairment in [A30P]alphaSyn transgenic mice. *Hum. Mol. Genet.* 23 3975–3989. 10.1093/hmg/ddu112 24619358 PMC4110482

[B102] Dikiy I. Eliezer D. (2014). N-terminal acetylation stabilizes N-terminal helicity in lipid- and micelle-bound alpha-synuclein and increases its affinity for physiological membranes. *J. Biol. Chem.* 289 3652–3665. 10.1074/jbc.M113.512459 24338013 PMC3916564

[B103] Doxakis E. (2010). Post-transcriptional regulation of alpha-synuclein expression by mir-7 and mir-153. *J. Biol. Chem.* 285 12726–12734. 10.1074/jbc.M109.086827 20106983 PMC2857101

[B104] Du T. T. Wang L. Duan C. L. Lu L. L. Zhang J. L. Gao G. (2015). GBA deficiency promotes SNCA/alpha-synuclein accumulation through autophagic inhibition by inactivated PPP2A. *Autophagy* 11 1803–1820. 10.1080/15548627.2015.1086055 26378614 PMC4824589

[B105] Dulovic M. Jovanovic M. Xilouri M. Stefanis L. Harhaji-Trajkovic L. Kravic-Stevovic T. (2014). The protective role of AMP-activated protein kinase in alpha-synuclein neurotoxicity *in vitro*. *Neurobiol. Dis.* 63 1–11. 10.1016/j.nbd.2013.11.002 24269733

[B106] Ebrahimi-Fakhari D. Cantuti-Castelvetri I. Fan Z. Rockenstein E. Masliah E. Hyman B. T. (2011). Distinct roles *in vivo* for the ubiquitin-proteasome system and the autophagy-lysosomal pathway in the degradation of alpha-synuclein. *J. Neurosci.* 31 14508–14520. 10.1523/JNEUROSCI.1560-11.2011 21994367 PMC3587176

[B107] El Turk F. De Genst E. Guilliams T. Fauvet B. Hejjaoui M. Di Trani J. (2018). Exploring the role of post-translational modifications in regulating alpha-synuclein interactions by studying the effects of phosphorylation on nanobody binding. *Protein Sci.* 27 1262–1274. 10.1002/pro.3412 29603451 PMC6032363

[B108] El-Agnaf O. Overk C. Rockenstein E. Mante M. Florio J. Adame A. (2017). Differential effects of immunotherapy with antibodies targeting alpha-synuclein oligomers and fibrils in a transgenic model of synucleinopathy. *Neurobiol. Dis.* 104 85–96. 10.1016/j.nbd.2017.05.002 28476636 PMC5954414

[B109] El-Agnaf O. M. Jakes R. Curran M. D. Middleton D. Ingenito R. Bianchi E. (1998). Aggregates from mutant and wild-type alpha-synuclein proteins and NAC peptide induce apoptotic cell death in human neuroblastoma cells by formation of beta-sheet and amyloid-like filaments. *FEBS Lett.* 440 71–75. 10.1016/s0014-5793(98)01418-5 9862428

[B110] El-Agnaf O. M. Paleologou K. E. Greer B. Abogrein A. M. King J. E. Salem S. A. (2004). A strategy for designing inhibitors of alpha-synuclein aggregation and toxicity as a novel treatment for Parkinson’s disease and related disorders. *FASEB J.* 18 1315–1317. 10.1096/fj.03-1346fje 15180968

[B111] El-Agnaf O. M. Salem S. A. Paleologou K. E. Cooper L. J. Fullwood N. J. Gibson M. J. (2003). Alpha-synuclein implicated in Parkinson’s disease is present in extracellular biological fluids, including human plasma. *FASEB J.* 17 1945–1947. 10.1096/fj.03-0098fje 14519670

[B112] Elkouris M. Kouroupi G. Vourvoukelis A. Papagiannakis N. Kaltezioti V. Matsas R. (2019). Long non-coding RNAs associated with neurodegeneration-linked genes are reduced in Parkinson’s disease patients. *Front. Cell. Neurosci.* 13:58. 10.3389/fncel.2019.00058 30853899 PMC6396023

[B113] Emadi S. Barkhordarian H. Wang M. S. Schulz P. Sierks M. R. (2007). Isolation of a human single chain antibody fragment against oligomeric alpha-synuclein that inhibits aggregation and prevents alpha-synuclein-induced toxicity. *J. Mol. Biol.* 368 1132–1144. 10.1016/j.jmb.2007.02.089 17391701 PMC2235820

[B114] Emadi S. Kasturirangan S. Wang M. S. Schulz P. Sierks M. R. (2009). Detecting morphologically distinct oligomeric forms of alpha-synuclein. *J. Biol. Chem.* 284 11048–11058. 10.1074/jbc.M806559200 19141614 PMC2670110

[B115] Emmanouilidou E. Melachroinou K. Roumeliotis T. Garbis S. D. Ntzouni M. Margaritis L. H. (2010a). Cell-produced alpha-synuclein is secreted in a calcium-dependent manner by exosomes and impacts neuronal survival. *J. Neurosci.* 30 6838–6851. 10.1523/JNEUROSCI.5699-09.2010 20484626 PMC3842464

[B116] Emmanouilidou E. Stefanis L. Vekrellis K. (2010b). Cell-produced alpha-synuclein oligomers are targeted to, and impair, the 26S proteasome. *Neurobiol. Aging* 31 953–968. 10.1016/j.neurobiolaging.2008.07.008 18715677

[B117] Ettle B. Reiprich S. Deusser J. Schlachetzki J. C. Xiang W. Prots I. (2014). Intracellular alpha-synuclein affects early maturation of primary oligodendrocyte progenitor cells. *Mol. Cell. Neurosci.* 62 68–78. 10.1016/j.mcn.2014.06.012 25019582 PMC4170075

[B118] Fahn S. Oakes D. Shoulson I. Kieburtz K. Rudolph A. Lang A. (2004). Levodopa and the progression of Parkinson’s disease. *N. Engl. J. Med.* 351 2498–2508. 10.1056/NEJMoa033447 15590952

[B119] Fanciulli A. Wenning G. K. (2015). Multiple-system atrophy. *N. Engl. J. Med.* 372 249–263. 10.1056/NEJMra1311488 25587949

[B120] Fasano A. Visanji N. P. Liu L. W. Lang A. E. Pfeiffer R. F. (2015). Gastrointestinal dysfunction in Parkinson’s disease. *Parkinsonism Relat. Disord.* 14 625–639. 10.1016/S1474-4422(15)00007-1 25987282

[B121] Fauvet B. Mbefo M. K. Fares M. B. Desobry C. Michael S. Ardah M. T. (2012). alpha-Synuclein in central nervous system and from erythrocytes, mammalian cells, and *Escherichia coli* exists predominantly as disordered monomer. *J. Biol. Chem.* 287 15345–15364. 10.1074/jbc.M111.318949 22315227 PMC3346117

[B122] Felice V. D. Quigley E. M. Sullivan A. M. O’Keeffe G. W. O’Mahony S. M. (2016). Microbiota-gut-brain signalling in Parkinson’s disease: implications for non-motor symptoms. *Parkinsonism Relat. Disord.* 27 1–8. 10.1016/j.parkreldis.2016.03.012 27013171

[B123] Fellner L. Buchinger E. Brueck D. Irschick R. Wenning G. K. Stefanova N. (2018). Limited effects of dysfunctional macroautophagy on the accumulation of extracellularly derived alpha-synuclein in oligodendroglia: implications for MSA pathogenesis. *BMC Neurosci.* 19:32. 10.1186/s12868-018-0431-2 29783943 PMC5963177

[B124] Fellner L. Kuzdas-Wood D. Levin J. Ryazanov S. Leonov A. Griesinger C. (2016). Anle138b partly ameliorates motor deficits despite failure of neuroprotection in a model of advanced multiple system atrophy. *Front. Neurosci.* 10:99. 10.3389/fnins.2016.00099 27013960 PMC4785146

[B125] Fellner L. Stefanova N. (2013). The role of glia in alpha-synucleinopathies. *Mol. Neurobiol.* 47 575–586. 10.1007/s12035-012-8340-3 22941028 PMC3589649

[B126] Ferreira D. G. Temido-Ferreira M. Vicente Miranda H. Batalha V. L. Coelho J. E. Szego E. M. (2017). alpha-synuclein interacts with PrP(C) to induce cognitive impairment through mGluR5 and NMDAR2B. *Nat. Neurosci.* 20 1569–1579. 10.1038/nn.4648 28945221

[B127] Ferreon A. C. Deniz A. A. (2007). Alpha-synuclein multistate folding thermodynamics: implications for protein misfolding and aggregation. *Biochemistry* 46 4499–4509. 10.1021/bi602461y 17378587

[B128] Ferretta A. Gaballo A. Tanzarella P. Piccoli C. Capitanio N. Nico B. (2014). Effect of resveratrol on mitochondrial function: implications in parkin-associated familiar Parkinson’s disease. *Biochim. Biophys. Acta* 1842 902–915. 10.1016/j.bbadis.2014.02.010 24582596

[B129] Fonseca-Ornelas L. Eisbach S. E. Paulat M. Giller K. Fernandez C. O. Outeiro T. F. (2014). Small molecule-mediated stabilization of vesicle-associated helical alpha-synuclein inhibits pathogenic misfolding and aggregation. *Nat. Commun.* 5:5857. 10.1038/ncomms6857 25524885

[B130] Fountaine T. M. Wade-Martins R. (2007). RNA interference-mediated knockdown of alpha-synuclein protects human dopaminergic neuroblastoma cells from MPP(+) toxicity and reduces dopamine transport. *J. Neurosci. Res.* 85 351–363. 10.1002/jnr.21125 17131421

[B131] Fredenburg R. A. Rospigliosi C. Meray R. K. Kessler J. C. Lashuel H. A. Eliezer D. (2007). The impact of the E46K mutation on the properties of alpha-synuclein in its monomeric and oligomeric states. *Biochemistry* 46 7107–7118. 10.1021/bi7000246 17530780

[B132] Friess E. Kuempfel T. Modell S. Winkelmann J. Holsboer F. Ising M. (2006). Paroxetine treatment improves motor symptoms in patients with multiple system atrophy. *Parkinsonism Relat. Disord.* 12 432–437. 10.1016/j.parkreldis.2006.04.002 16769235

[B133] Fu H. Subramanian R. R. Masters S. C. (2000). 14-3-3 proteins: structure, function, and regulation. *Annu. Rev. Pharmacol. Toxicol.* 40 617–647. 10.1146/annurev.pharmtox.40.1.617 10836149

[B134] Fujiwara H. Hasegawa M. Dohmae N. Kawashima A. Masliah E. Goldberg M. S. (2002). alpha-Synuclein is phosphorylated in synucleinopathy lesions. *Nat. Cell Biol.* 4 160–164. 10.1038/ncb748 11813001

[B135] Fusco G. Chen S. W. Williamson P. T. F. Cascella R. Perni M. Jarvis J. A. (2017). Structural basis of membrane disruption and cellular toxicity by alpha-synuclein oligomers. *Science* 358 1440–1443. 10.1126/science.aan6160 29242346

[B136] Fussi N. Hollerhage M. Chakroun T. Nykanen N. P. Rosler T. W. Koeglsperger T. (2018). Exosomal secretion of alpha-synuclein as protective mechanism after upstream blockage of macroautophagy. *Cell Death Dis.* 9:757. 10.1038/s41419-018-0816-2 29988147 PMC6037700

[B137] Games D. Valera E. Spencer B. Rockenstein E. Mante M. Adame A. (2014). Reducing C-terminal-truncated alpha-synuclein by immunotherapy attenuates neurodegeneration and propagation in Parkinson’s disease-like models. *J. Neurosci.* 34 9441–9454. 10.1523/JNEUROSCI.5314-13.2014 25009275 PMC4087215

[B138] Ganjam G. K. Bolte K. Matschke L. A. Neitemeier S. Dolga A. M. Hollerhage M. (2019). Mitochondrial damage by alpha-synuclein causes cell death in human dopaminergic neurons. *Cell Death Dis.* 10:865. 10.1038/s41419-019-2091-2 31727879 PMC6856124

[B139] Garcia-Reitbock P. Anichtchik O. Bellucci A. Iovino M. Ballini C. Fineberg E. (2010). SNARE protein redistribution and synaptic failure in a transgenic mouse model of Parkinson’s disease. *Brain* 133(Pt 7), 2032–2044. 10.1093/brain/awq132 20534649 PMC2892942

[B140] Gendelman H. E. Zhang Y. Santamaria P. Olson K. E. Schutt C. R. Bhatti D. (2017). Evaluation of the safety and immunomodulatory effects of sargramostim in a randomized, double-blind phase 1 clinical Parkinson’s disease trial. *NPJ Parkinsons Dis.* 3:10. 10.1038/s41531-017-0013-5 28649610 PMC5445595

[B141] George S. Rey N. L. Tyson T. Esquibel C. Meyerdirk L. Schulz E. (2019). Microglia affect alpha-synuclein cell-to-cell transfer in a mouse model of Parkinson’s disease. *Mol. Neurodegener.* 14:34. 10.1186/s13024-019-0335-3 31419995 PMC6697982

[B142] Giasson B. I. Duda J. E. Quinn S. M. Zhang B. Trojanowski J. Q. Lee V. M. (2002). Neuronal alpha-synucleinopathy with severe movement disorder in mice expressing A53T human alpha-synuclein. *Neuron* 34 521–533. 10.1016/s0896-6273(02)00682-7 12062037

[B143] Giasson B. I. Forman M. S. Higuchi M. Golbe L. I. Graves C. L. Kotzbauer P. T. (2003). Initiation and synergistic fibrillization of tau and alpha-synuclein. *Science* 300 636–640. 10.1126/science.1082324 12714745

[B144] Giasson B. I. Murray I. V. Trojanowski J. Q. Lee V. M. (2001). A hydrophobic stretch of 12 amino acid residues in the middle of alpha-synuclein is essential for filament assembly. *J. Biol. Chem.* 276 2380–2386. 10.1074/jbc.M008919200 11060312

[B145] Gitler A. D. Bevis B. J. Shorter J. Strathearn K. E. Hamamichi S. Su L. J. (2008). The Parkinson’s disease protein alpha-synuclein disrupts cellular Rab homeostasis. *Proc. Natl. Acad. Sci. U.S.A.* 105 145–150. 10.1073/pnas.0710685105 18162536 PMC2224176

[B146] Gonzalez N. Arcos-Lopez T. Konig A. Quintanar L. Menacho Marquez M. Outeiro T. F. (2019). Effects of alpha-synuclein post-translational modifications on metal binding. *J. Neurochem.* 150 507–521. 10.1111/jnc.14721 31099098

[B147] Gorbatyuk O. S. Li S. Nash K. Gorbatyuk M. Lewin A. S. Sullivan L. F. (2010). *In vivo* RNAi-mediated alpha-synuclein silencing induces nigrostriatal degeneration. *Mol. Ther.* 18 1450–1457. 10.1038/mt.2010.115 20551914 PMC2927065

[B148] Gould N. Mor D. E. Lightfoot R. Malkus K. Giasson B. Ischiropoulos H. (2014). Evidence of native alpha-synuclein conformers in the human brain. *J. Biol. Chem.* 289 7929–7934. 10.1074/jbc.C113.538249 24474688 PMC3953303

[B149] Grassi D. Howard S. Zhou M. Diaz-Perez N. Urban N. T. Guerrero-Given D. (2018). Identification of a highly neurotoxic alpha-synuclein species inducing mitochondrial damage and mitophagy in Parkinson’s disease. *Proc. Natl. Acad. Sci. U.S.A.* 115 E2634–E2643. 10.1073/pnas.1713849115 29487216 PMC5856519

[B150] Grathwohl S. A. Steiner J. A. Britschgi M. Brundin P. (2013). Mind the gut: secretion of alpha-synuclein by enteric neurons. *J. Neurochem.* 125 487–490. 10.1111/jnc.12191 23448303

[B151] Guardia-Laguarta C. Area-Gomez E. Rub C. Liu Y. Magrane J. Becker D. (2014). alpha-Synuclein is localized to mitochondria-associated ER membranes. *J. Neurosci.* 34 249–259. 10.1523/JNEUROSCI.2507-13.2014 24381286 PMC3866487

[B152] Guo J. L. Covell D. J. Daniels J. P. Iba M. Stieber A. Zhang B. (2013). Distinct alpha-synuclein strains differentially promote tau inclusions in neurons. *Cell* 154 103–117. 10.1016/j.cell.2013.05.057 23827677 PMC3820001

[B153] Guo Y. J. Dong S. Y. Cui X. X. Feng Y. Liu T. Yin M. (2016). Resveratrol alleviates MPTP-induced motor impairments and pathological changes by autophagic degradation of alpha-synuclein via SIRT1-deacetylated LC3. *Mol. Nutr. Food Res.* 60 2161–2175. 10.1002/mnfr.201600111 27296520 PMC6089356

[B154] Gustafsson G. Lindstrom V. Rostami J. Nordstrom E. Lannfelt L. Bergstrom J. (2017). Alpha-synuclein oligomer-selective antibodies reduce intracellular accumulation and mitochondrial impairment in alpha-synuclein exposed astrocytes. *J. Neuroinflammation* 14:241. 10.1186/s12974-017-1018-z 29228971 PMC5725978

[B155] Gustafsson G. Loov C. Persson E. Lazaro D. F. Takeda S. Bergstrom J. (2018). Secretion and uptake of alpha-synuclein via extracellular vesicles in cultured cells. *Cell. Mol. Neurobiol.* 38 1539–1550. 10.1007/s10571-018-0622-5 30288631 PMC6223723

[B156] Haj-Yahya M. Fauvet B. Herman-Bachinsky Y. Hejjaoui M. Bavikar S. N. Karthikeyan S. V. (2013). Synthetic polyubiquitinated alpha-Synuclein reveals important insights into the roles of the ubiquitin chain in regulating its pathophysiology. *Proc. Natl. Acad. Sci. U.S.A.* 110 17726–17731. 10.1073/pnas.1315654110 24043770 PMC3816408

[B157] Hall K. Yang S. Sauchanka O. Spillantini M. G. Anichtchik O. (2015). Behavioural deficits in transgenic mice expressing human truncated (1-120 amino acid) alpha-synuclein. *Exp. Neurol.* 264 8–13. 10.1016/j.expneurol.2014.11.003 25450466

[B158] Han Y. Khodr C. E. Sapru M. K. Pedapati J. Bohn M. C. (2011). A microRNA embedded AAV alpha-synuclein gene silencing vector for dopaminergic neurons. *Brain Res.* 1386 15–24. 10.1016/j.brainres.2011.02.041 21338582 PMC3076891

[B159] Hansen C. Angot E. Bergstrom A. L. Steiner J. A. Pieri L. Paul G. (2011). alpha-Synuclein propagates from mouse brain to grafted dopaminergic neurons and seeds aggregation in cultured human cells. *J. Clin. Invest.* 121 715–725. 10.1172/JCI43366 21245577 PMC3026723

[B160] Hassen G. W. Kesner L. Stracher A. Shulman A. Rockenstein E. Mante M. (2018). Effects of novel calpain inhibitors in transgenic animal model of Parkinson’s disease/dementia with Lewy bodies. *Sci. Rep.* 8:18083. 10.1038/s41598-018-35729-1 30591714 PMC6308237

[B161] Hayashita-Kinoh H. Yamada M. Yokota T. Mizuno Y. Mochizuki H. (2006). Down-regulation of alpha-synuclein expression can rescue dopaminergic cells from cell death in the substantia nigra of Parkinson’s disease rat model. *Biochem. Biophys. Res. Commun.* 341 1088–1095. 10.1016/j.bbrc.2006.01.057 16460685

[B162] He Q. Koprich J. B. Wang Y. Yu W. B. Xiao B. G. Brotchie J. M. (2016). Treatment with trehalose prevents behavioral and neurochemical deficits produced in an AAV alpha-synuclein rat model of Parkinson’s disease. *Mol. Neurobiol.* 53 2258–2268. 10.1007/s12035-015-9173-7 25972237

[B163] Hebron M. L. Lonskaya I. Moussa C. E. (2013). Nilotinib reverses loss of dopamine neurons and improves motor behavior via autophagic degradation of alpha-synuclein in Parkinson’s disease models. *Hum. Mol. Genet.* 22 3315–3328. 10.1093/hmg/ddt192 23666528 PMC3723316

[B164] Hellstrand E. Nowacka A. Topgaard D. Linse S. Sparr E. (2013). Membrane lipid co-aggregation with alpha-synuclein fibrils. *PLoS One* 8:e77235. 10.1371/journal.pone.0077235 24146972 PMC3795653

[B165] Heras-Garvin A. Weckbecker D. Ryazanov S. Leonov A. Griesinger C. Giese A. (2019). Anle138b modulates alpha-synuclein oligomerization and prevents motor decline and neurodegeneration in a mouse model of multiple system atrophy. *Mov. Disord.* 34 255–263. 10.1002/mds.27562 30452793 PMC6492169

[B166] Herrera-Vaquero M. Bouquio D. Kallab M. Biggs K. Nair G. Ochoa J. (2019). The molecular tweezer CLR01 reduces aggregated, pathologic, and seeding-competent alpha-synuclein in experimental multiple system atrophy. *Biochim. Biophys. Acta Mol. Basis Dis.* 1865:165513. 10.1016/j.bbadis.2019.07.007 31319154 PMC8425273

[B167] Ho P. W. Leung C. T. Liu H. Pang S. Y. Lam C. S. Xian J. (2020). Age-dependent accumulation of oligomeric SNCA/alpha-synuclein from impaired degradation in mutant LRRK2 knockin mouse model of Parkinson disease: role for therapeutic activation of chaperone-mediated autophagy (CMA). *Autophagy* 16 347–370. 10.1080/15548627.2019.1603545 30983487 PMC6984454

[B168] Holmes B. B. DeVos S. L. Kfoury N. Li M. Jacks R. Yanamandra K. (2013). Heparan sulfate proteoglycans mediate internalization and propagation of specific proteopathic seeds. *Proc. Natl. Acad. Sci. U.S.A.* 110 E3138–E3147. 10.1073/pnas.1301440110 23898162 PMC3746848

[B169] Holmqvist S. Chutna O. Bousset L. Aldrin-Kirk P. Li W. Bjorklund T. (2014). Direct evidence of Parkinson pathology spread from the gastrointestinal tract to the brain in rats. *Acta Neuropathol.* 128 805–820. 10.1007/s00401-014-1343-6 25296989

[B170] Horvath I. Sellstedt M. Weise C. Nordvall L. M. Krishna Prasad G. Olofsson A. (2013). Modulation of alpha-synuclein fibrillization by ring-fused 2-pyridones: templation and inhibition involve oligomers with different structure. *Arch. Biochem. Biophys.* 532 84–90. 10.1016/j.abb.2013.01.012 23399432

[B171] Houser M. C. Tansey M. G. (2017). The gut-brain axis: is intestinal inflammation a silent driver of Parkinson’s disease pathogenesis? *NPJ. Parkinsons Dis.* 3:3. 10.1038/s41531-016-0002-0 28649603 PMC5445611

[B172] Hughes C. D. Choi M. L. Ryten M. Hopkins L. Drews A. Botia J. A. (2019). Picomolar concentrations of oligomeric alpha-synuclein sensitizes TLR4 to play an initiating role in Parkinson’s disease pathogenesis. *Acta Neuropathol.* 137 103–120. 10.1007/s00401-018-1907-y 30225556 PMC6338693

[B173] Hung K. C. Huang H. J. Wang Y. T. Lin A. M. (2016). Baicalein attenuates alpha-synuclein aggregation, inflammasome activation and autophagy in the MPP(+)-treated nigrostriatal dopaminergic system *in vivo*. *J. Ethnopharmacol.* 194 522–529. 10.1016/j.jep.2016.10.040 27742410

[B174] Ihse E. Yamakado H. van Wijk X. M. Lawrence R. Esko J. D. Masliah E. (2017). Cellular internalization of alpha-synuclein aggregates by cell surface heparan sulfate depends on aggregate conformation and cell type. *Sci. Rep.* 7:9008. 10.1038/s41598-017-08720-5 28827536 PMC5566500

[B175] Ii M. Matsunaga N. Hazeki K. Nakamura K. Takashima K. Seya T. (2006). A novel cyclohexene derivative, ethyl (6R)-6-[N-(2-Chloro-4-fluorophenyl)sulfamoyl]cyclohex-1-ene-1-carboxylate (TAK-242), selectively inhibits toll-like receptor 4-mediated cytokine production through suppression of intracellular signaling. *Mol. Pharmacol.* 69 1288–1295. 10.1124/mol.105.019695 16373689

[B176] Ishizawa K. Komori T. Sasaki S. Arai N. Mizutani T. Hirose T. (2004). Microglial activation parallels system degeneration in multiple system atrophy. *J. Neuropathol. Exp. Neurol.* 63 43–52. 10.1093/jnen/63.1.43 14748560

[B177] Issa A. R. Sun J. Petitgas C. Mesquita A. Dulac A. Robin M. (2018). The lysosomal membrane protein LAMP2A promotes autophagic flux and prevents SNCA-induced Parkinson disease-like symptoms in the Drosophila brain. *Autophagy* 14 1898–1910. 10.1080/15548627.2018.1491489 29989488 PMC6152503

[B178] Iyer A. Roeters S. J. Kogan V. Woutersen S. Claessens M. Subramaniam V. (2017). C-terminal truncated alpha-synuclein fibrils contain strongly twisted beta-Sheets. *J. Am. Chem. Soc.* 139 15392–15400. 10.1021/jacs.7b07403 28968082 PMC5668890

[B179] Jakes R. Spillantini M. G. Goedert M. (1994). Identification of two distinct synucleins from human brain. *FEBS Lett.* 345 27–32. 10.1016/0014-5793(94)00395-5 8194594

[B180] Jang A. Lee H. J. Suk J. E. Jung J. W. Kim K. P. Lee S. J. (2010). Non-classical exocytosis of alpha-synuclein is sensitive to folding states and promoted under stress conditions. *J. Neurochem.* 113 1263–1274. 10.1111/j.1471-4159.2010.06695.x 20345754

[B181] Jankovic J. Goodman I. Safirstein B. Marmon T. K. Schenk D. B. Koller M. (2018). Safety and tolerability of multiple ascending doses of PRX002/RG7935, an anti-alpha-synuclein monoclonal antibody, in patients with Parkinson disease: a randomized clinical trial. *JAMA Neurol.* 75 1206–1214. 10.1001/jamaneurol.2018.1487 29913017 PMC6233845

[B182] Jao C. C. Hegde B. G. Chen J. Haworth I. S. Langen R. (2008). Structure of membrane-bound alpha-synuclein from site-directed spin labeling and computational refinement. *Proc. Natl. Acad. Sci. U.S.A.* 105 19666–19671. 10.1073/pnas.0807826105 19066219 PMC2605001

[B183] Javed H. Nagoor Meeran M. F. Azimullah S. Adem A. Sadek B. Ojha S. K. (2018). Plant extracts and phytochemicals targeting alpha-synuclein aggregation in Parkinson’s disease models. *Front. Pharmacol.* 9:1555. 10.3389/fphar.2018.01555 30941047 PMC6433754

[B184] Jeannotte A. M. Sidhu A. (2007). Regulation of the norepinephrine transporter by alpha-synuclein-mediated interactions with microtubules. *Eur. J. Neurosci.* 26 1509–1520. 10.1111/j.1460-9568.2007.05757.x 17714497

[B185] Jensen P. H. Nielsen M. S. Jakes R. Dotti C. G. Goedert M. (1998). Binding of alpha-synuclein to brain vesicles is abolished by familial Parkinson’s disease mutation. *J. Biol. Chem.* 273 26292–26294. 10.1074/jbc.273.41.26292 9756856

[B186] Ji H. Liu Y. E. Jia T. Wang M. Liu J. Xiao G. (1997). Identification of a breast cancer-specific gene. BCSG1, by direct differential cDNA sequencing. *Cancer Res.* 57 759–764. 9044857

[B187] Jiang P. Gan M. Yen S. H. McLean P. J. Dickson D. W. (2017). Impaired endo-lysosomal membrane integrity accelerates the seeding progression of alpha-synuclein aggregates. *Sci. Rep.* 7:7690. 10.1038/s41598-017-08149-w 28794446 PMC5550496

[B188] Jiang T. F. Zhang Y. J. Zhou H. Y. Wang H. M. Tian L. P. Liu J. (2013). Curcumin ameliorates the neurodegenerative pathology in A53T alpha-synuclein cell model of Parkinson’s disease through the downregulation of mTOR/p70S6K signaling and the recovery of macroautophagy. *J. Neuroimmune Pharmacol.* 8 356–369. 10.1007/s11481-012-9431-7 23325107

[B189] Jin H. Kanthasamy A. Ghosh A. Yang Y. Anantharam V. Kanthasamy A. G. (2011). alpha-Synuclein negatively regulates protein kinase Cdelta expression to suppress apoptosis in dopaminergic neurons by reducing p300 histone acetyltransferase activity. *J. Neurosci.* 31 2035–2051. 10.1523/JNEUROSCI.5634-10.2011 21307242 PMC3041642

[B190] Judith Peterschmitt M. Gasser T. Isaacson S. Kulisevsky J. Mir P. Simuni T. (2019). Safety, tolerability and pharmacokinetics of oral venglustat in Parkinson disease patients with a GBA mutation. *Mol. Genet. Metab.* 126:S117.

[B191] Junn E. Lee K. W. Jeong B. S. Chan T. W. Im J. Y. Mouradian M. M. (2009). Repression of alpha-synuclein expression and toxicity by microRNA-7. *Proc. Natl. Acad. Sci. U.S.A.* 106 13052–13057. 10.1073/pnas.0906277106 19628698 PMC2722353

[B192] Kahle P. J. Neumann M. Ozmen L. Muller V. Jacobsen H. Spooren W. (2002). Hyperphosphorylation and insolubility of alpha-synuclein in transgenic mouse oligodendrocytes. *EMBO Rep.* 3 583–588. 10.1093/embo-reports/kvf109 12034752 PMC1084143

[B193] Kaji S. Maki T. Kinoshita H. Uemura N. Ayaki T. Kawamoto Y. (2018). Pathological endogenous alpha-synuclein accumulation in oligodendrocyte precursor cells potentially induces inclusions in multiple system atrophy. *Stem Cell Rep.* 10 356–365. 10.1016/j.stemcr.2017.12.001 29337114 PMC5830961

[B194] Kallab M. Herrera-Vaquero M. Johannesson M. Eriksson F. Sigvardson J. Poewe W. (2018). Region-specific effects of immunotherapy with antibodies targeting alpha-synuclein in a transgenic model of synucleinopathy. *Front. Neurosci.* 12:452. 10.3389/fnins.2018.00452 30022929 PMC6039792

[B195] Kamp F. Exner N. Lutz A. K. Wender N. Hegermann J. Brunner B. (2010). Inhibition of mitochondrial fusion by alpha-synuclein is rescued by PINK1, Parkin and DJ-1. *EMBO J.* 29 3571–3589. 10.1038/emboj.2010.223 20842103 PMC2964170

[B196] Kanaan N. M. Manfredsson F. P. (2012). Loss of functional alpha-synuclein: A toxic event in Parkinson’s disease? *J. Parkinsons Dis.* 2 249–267. 10.3233/JPD-012138 23938255 PMC4736738

[B197] Karampetsou M. Ardah M. T. Semitekolou M. Polissidis A. Samiotaki M. Kalomoiri M. (2017). Phosphorylated exogenous alpha-synuclein fibrils exacerbate pathology and induce neuronal dysfunction in mice. *Sci. Rep.* 7:16533. 10.1038/s41598-017-15813-8 29184069 PMC5705684

[B198] Karpinar D. P. Balija M. B. Kugler S. Opazo F. Rezaei-Ghaleh N. Wender N. (2009). Pre-fibrillar alpha-synuclein variants with impaired beta-structure increase neurotoxicity in Parkinson’s disease models. *EMBO J.* 28 3256–3268. 10.1038/emboj.2009.257 19745811 PMC2771093

[B199] Karpowicz R. J. Jr. Haney C. M. Mihaila T. S. Sandler R. M. Petersson E. J. Lee V. M. (2017). Selective imaging of internalized proteopathic alpha-synuclein seeds in primary neurons reveals mechanistic insight into transmission of synucleinopathies. *J. Biol. Chem.* 292 13482–13497. 10.1074/jbc.M117.780296 28611062 PMC5555207

[B200] Karuppagounder S. S. Brahmachari S. Lee Y. Dawson V. L. Dawson T. M. Ko H. S. (2014). The c-Abl inhibitor, nilotinib, protects dopaminergic neurons in a preclinical animal model of Parkinson’s disease. *Sci. Rep.* 4:4874. 10.1038/srep04874 24786396 PMC4007078

[B201] Kaufman E. Hall S. Surova Y. Widner H. Hansson O. Lindqvist D. (2013). Proinflammatory cytokines are elevated in serum of patients with multiple system atrophy. *PLoS One* 8:e62354. 10.1371/journal.pone.0062354 23626805 PMC3633844

[B202] Kawamoto T. Ii M. Kitazaki T. Iizawa Y. Kimura H. (2008). TAK-242 selectively suppresses Toll-like receptor 4-signaling mediated by the intracellular domain. *Eur. J. Pharmacol.* 584 40–48. 10.1016/j.ejphar.2008.01.026 18299127

[B203] Kayed R. Sokolov Y. Edmonds B. McIntire T. M. Milton S. C. Hall J. E. (2004). Permeabilization of lipid bilayers is a common conformation-dependent activity of soluble amyloid oligomers in protein misfolding diseases. *J. Biol. Chem.* 279 46363–46366. 10.1074/jbc.C400260200 15385542

[B204] Kellie J. F. Higgs R. E. Ryder J. W. Major A. Beach T. G. Adler C. H. (2014). Quantitative measurement of intact alpha-synuclein proteoforms from post-mortem control and Parkinson’s disease brain tissue by intact protein mass spectrometry. *Sci. Rep.* 4:5797. 10.1038/srep05797 25052239 PMC4107347

[B205] Khalaf O. Fauvet B. Oueslati A. Dikiy I. Mahul-Mellier A. L. Ruggeri F. S. (2014). The H50Q mutation enhances alpha-synuclein aggregation, secretion, and toxicity. *J. Biol. Chem.* 289 21856–21876. 10.1074/jbc.M114.553297 24936070 PMC4139205

[B206] Khodr C. E. Becerra A. Han Y. Bohn M. C. (2014). Targeting alpha-synuclein with a microRNA-embedded silencing vector in the rat substantia nigra: positive and negative effects. *Brain Res.* 1550 47–60. 10.1016/j.brainres.2014.01.010 24463035 PMC3974902

[B207] Khodr C. E. Sapru M. K. Pedapati J. Han Y. West N. C. Kells A. P. (2011). An alpha-synuclein AAV gene silencing vector ameliorates a behavioral deficit in a rat model of Parkinson’s disease, but displays toxicity in dopamine neurons. *Brain Res.* 1395 94–107. 10.1016/j.brainres.2011.04.036 21565333 PMC3105182

[B208] Kiely A. P. Asi Y. T. Kara E. Limousin P. Ling H. Lewis P. (2013). alpha-Synucleinopathy associated with G51D SNCA mutation: a link between Parkinson’s disease and multiple system atrophy? *Acta Neuropathol.* 125 753–769. 10.1007/s00401-013-1096-7 23404372 PMC3681325

[B209] Kiely A. P. Ling H. Asi Y. T. Kara E. Proukakis C. Schapira A. H. (2015). Distinct clinical and neuropathological features of G51D SNCA mutation cases compared with SNCA duplication and H50Q mutation. *Mol. Neurodegener.* 10:41. 10.1186/s13024-015-0038-3 26306801 PMC4549856

[B210] Kim C. Ho D. H. Suk J. E. You S. Michael S. Kang J. (2013). Neuron-released oligomeric alpha-synuclein is an endogenous agonist of TLR2 for paracrine activation of microglia. *Nat. Commun.* 4:1562. 10.1038/ncomms2534 23463005 PMC4089961

[B211] Kim C. Lv G. Lee J. S. Jung B. C. Masuda-Suzukake M. Hong C. S. (2016). Exposure to bacterial endotoxin generates a distinct strain of alpha-synuclein fibril. *Sci. Rep.* 6:30891. 10.1038/srep30891 27488222 PMC4973277

[B212] Kim S. Kwon S. H. Kam T. I. Panicker N. Karuppagounder S. S. Lee S. (2019). Transneuronal propagation of pathologic alpha-synuclein from the gut to the brain models Parkinson’s disease. *Neuron* 103 627–641.e7. 10.1016/j.neuron.2019.05.035 31255487 PMC6706297

[B213] Kim T. Mehta S. L. Morris-Blanco K. C. Chokkalla A. K. Chelluboina B. Lopez M. (2018). The microRNA miR-7a-5p ameliorates ischemic brain damage by repressing alpha-synuclein. *Sci. Signal.* 11:eaat4285. 10.1126/scisignal.aat4285 30538177 PMC7005928

[B214] Kim T. D. Paik S. R. Yang C. H. (2002). Structural and functional implications of C-terminal regions of alpha-synuclein. *Biochemistry* 41 13782–13790. 10.1021/bi026284c 12427041

[B215] Kim Y. S. Lim D. Kim J. Y. Kang S. J. Kim Y. H. Im H. (2009). beta-Sheet-breaking peptides inhibit the fibrillation of human alpha-synuclein. *Biochem. Biophys. Res. Commun.* 387 682–687. 10.1016/j.bbrc.2009.07.083 19622344

[B216] Kirik D. Rosenblad C. Burger C. Lundberg C. Johansen T. E. Muzyczka N. (2002). Parkinson-like neurodegeneration induced by targeted overexpression of alpha-synuclein in the nigrostriatal system. *J. Neurosci.* 22 2780–2791. 10.1523/jneurosci.22-07-02780.2002 11923443 PMC6758323

[B217] Kisos H. Pukass K. Ben-Hur T. Richter-Landsberg C. Sharon R. (2012). Increased neuronal alpha-synuclein pathology associates with its accumulation in oligodendrocytes in mice modeling alpha-synucleinopathies. *PLoS One* 7:e46817. 10.1371/journal.pone.0046817 23077527 PMC3471961

[B218] Klegeris A. Pelech S. Giasson B. I. Maguire J. Zhang H. McGeer E. G. (2008). Alpha-synuclein activates stress signaling protein kinases in THP-1 cells and microglia. *Neurobiol. Aging* 29 739–752. 10.1016/j.neurobiolaging.2006.11.013 17166628

[B219] Klein A. D. Mazzulli J. R. (2018). Is Parkinson’s disease a lysosomal disorder? *Brain* 141 2255–2262. 10.1093/brain/awy147 29860491 PMC6061679

[B220] Kleinknecht A. Popova B. Lazaro D. F. Pinho R. Valerius O. Outeiro T. F. (2016). C-Terminal tyrosine residue modifications modulate the protective phosphorylation of serine 129 of alpha-synuclein in a yeast model of Parkinson’s disease. *PLoS Genet.* 12:e1006098. 10.1371/journal.pgen.1006098 27341336 PMC4920419

[B221] Konno M. Hasegawa T. Baba T. Miura E. Sugeno N. Kikuchi A. (2012). Suppression of dynamin GTPase decreases alpha-synuclein uptake by neuronal and oligodendroglial cells: a potent therapeutic target for synucleinopathy. *Mol. Neurodegener.* 7:38. 10.1186/1750-1326-7-38 22892036 PMC3479026

[B222] Kordower J. H. Chu Y. Hauser R. A. Freeman T. B. Olanow C. W. (2008). Lewy body-like pathology in long-term embryonic nigral transplants in Parkinson’s disease. *Nat. Med.* 14 504–506. 10.1038/nm1747 18391962

[B223] Kordower J. H. Dodiya H. B. Kordower A. M. Terpstra B. Paumier K. Madhavan L. (2011). Transfer of host-derived alpha synuclein to grafted dopaminergic neurons in rat. *Neurobiol. Dis.* 43 552–557. 10.1016/j.nbd.2011.05.001 21600984 PMC3430516

[B224] Kragh C. L. Lund L. B. Febbraro F. Hansen H. D. Gai W. P. El-Agnaf O. (2009). Alpha-synuclein aggregation and Ser-129 phosphorylation-dependent cell death in oligodendroglial cells. *J. Biol. Chem.* 284 10211–10222. 10.1074/jbc.M809671200 19203998 PMC2665075

[B225] Krishnan R. Tsubery H. Proschitsky M. Y. Asp E. Lulu M. Gilead S. (2014). A bacteriophage capsid protein provides a general amyloid interaction motif (GAIM) that binds and remodels misfolded protein assemblies. *J. Mol. Biol.* 426 2500–2519. 10.1016/j.jmb.2014.04.015 24768993

[B226] Kruger R. Kuhn W. Muller T. Woitalla D. Graeber M. Kosel S. (1998). Ala30Pro mutation in the gene encoding alpha-synuclein in Parkinson’s disease. *Nat. Genet.* 18 106–108. 10.1038/ng0298-106 9462735

[B227] Krumova P. Meulmeester E. Garrido M. Tirard M. Hsiao H. H. Bossis G. (2011). Sumoylation inhibits alpha-synuclein aggregation and toxicity. *J. Cell Biol.* 194 49–60. 10.1083/jcb.201010117 21746851 PMC3135405

[B228] Kurowska Z. Englund E. Widner H. Lindvall O. Li J. Y. Brundin P. (2011). Signs of degeneration in 12-22-year old grafts of mesencephalic dopamine neurons in patients with Parkinson’s disease. *J. Parkinsons Dis.* 1 83–92. 10.3233/JPD-2011-11004 23939259

[B229] Kuwahara T. Tonegawa R. Ito G. Mitani S. Iwatsubo T. (2012). Phosphorylation of alpha-synuclein protein at Ser-129 reduces neuronal dysfunction by lowering its membrane binding property in *Caenorhabditis elegans*. *J. Biol. Chem.* 287 7098–7109. 10.1074/jbc.M111.237131 22232559 PMC3293593

[B230] Lan D. M. Liu F. T. Zhao J. Chen Y. Wu J. J. Ding Z. T. (2012). Effect of trehalose on PC12 cells overexpressing wild-type or A53T mutant alpha-synuclein. *Neurochem. Res.* 37 2025–2032. 10.1007/s11064-012-0823-0 22707286

[B231] Lassot I. Mora S. Lesage S. Zieba B. A. Coque E. Condroyer C. (2018). The E3 Ubiquitin Ligases TRIM17 and TRIM41 modulate alpha-synuclein expression by regulating ZSCAN21. *Cell Rep.* 25 2484–2496.e9. 10.1016/j.celrep.2018.11.002 30485814

[B232] Lau A. So R. W. L. Lau H. H. C. Sang J. C. Ruiz-Riquelme A. Fleck S. C. (2020). alpha-Synuclein strains target distinct brain regions and cell types. *Nat. Neurosci.* 23 21–31. 10.1038/s41593-019-0541-x 31792467 PMC6930851

[B233] Lautenschlager J. Stephens A. D. Fusco G. Strohl F. Curry N. Zacharopoulou M. (2018). C-terminal calcium binding of alpha-synuclein modulates synaptic vesicle interaction. *Nat. Commun.* 9:712. 10.1038/s41467-018-03111-4 29459792 PMC5818535

[B234] Lazaro D. F. Rodrigues E. F. Langohr R. Shahpasandzadeh H. Ribeiro T. Guerreiro P. (2014). Systematic comparison of the effects of alpha-synuclein mutations on its oligomerization and aggregation. *PLoS Genet.* 10:e1004741. 10.1371/journal.pgen.1004741 25393002 PMC4230739

[B235] Lebouvier T. Neunlist M. Bruley des Varannes S. Coron E. Drouard A. N’Guyen J. M. (2010). Colonic biopsies to assess the neuropathology of Parkinson’s disease and its relationship with symptoms. *PLoS One* 5:e12728. 10.1371/journal.pone.0012728 20856865 PMC2939055

[B236] Lee H. J. Cho E. D. Lee K. W. Kim J. H. Cho S. G. Lee S. J. (2013). Autophagic failure promotes the exocytosis and intercellular transfer of alpha-synuclein. *Exp. Mol. Med.* 45:e22. 10.1038/emm.2013.45 23661100 PMC3674407

[B237] Lee H. J. Jung K. W. Chung S. J. Hong S. M. Kim J. Lee J. H. (2018). Relation of enteric alpha-synuclein to gastrointestinal dysfunction in patients with Parkinson’s Disease and in neurologically intact subjects. *J. Neurogastroenterol. Motil.* 24 469–478. 10.5056/jnm17141 29969861 PMC6034677

[B238] Lee H. J. Patel S. Lee S. J. (2005). Intravesicular localization and exocytosis of alpha-synuclein and its aggregates. *J. Neurosci.* 25 6016–6024. 10.1523/JNEUROSCI.0692-05.2005 15976091 PMC6724798

[B239] Lee H. J. Suk J. E. Bae E. J. Lee J. H. Paik S. R. Lee S. J. (2008a). Assembly-dependent endocytosis and clearance of extracellular alpha-synuclein. *Int. J. Biochem. Cell Biol.* 40 1835–1849. 10.1016/j.biocel.2008.01.017 18291704

[B240] Lee H. J. Suk J. E. Bae E. J. Lee S. J. (2008b). Clearance and deposition of extracellular alpha-synuclein aggregates in microglia. *Biochem. Biophys. Res. Commun.* 372 423–428. 10.1016/j.bbrc.2008.05.045 18492487

[B241] Lee H. J. Suk J. E. Patrick C. Bae E. J. Cho J. H. Rho S. (2010). Direct transfer of alpha-synuclein from neuron to astroglia causes inflammatory responses in synucleinopathies. *J. Biol. Chem.* 285 9262–9272. 10.1074/jbc.M109.081125 20071342 PMC2838344

[B242] Lee K. W. Chen W. Junn E. Im J. Y. Grosso H. Sonsalla P. K. (2011). Enhanced phosphatase activity attenuates alpha-synucleinopathy in a mouse model. *J. Neurosci.* 31 6963–6971. 10.1523/JNEUROSCI.6513-10.2011 21562258 PMC5038983

[B243] Lei Z. Cao G. Wei G. (2019). A30P mutant alpha-synuclein impairs autophagic flux by inactivating JNK signaling to enhance ZKSCAN3 activity in midbrain dopaminergic neurons. *Cell Death Dis.* 10:133. 10.1038/s41419-019-1364-0 30755581 PMC6372582

[B244] Levin J. Schmidt F. Boehm C. Prix C. Botzel K. Ryazanov S. (2014). The oligomer modulator anle138b inhibits disease progression in a Parkinson mouse model even with treatment started after disease onset. *Acta Neuropathol.* 127 779–780. 10.1007/s00401-014-1265-3 24615514 PMC3984662

[B245] Lewis J. Melrose H. Bumcrot D. Hope A. Zehr C. Lincoln S. (2008). *In vivo* silencing of alpha-synuclein using naked siRNA. *Mol. Neurodegener.* 3:19. 10.1186/1750-1326-3-19 18976489 PMC2612658

[B246] Li J. Zhu M. Rajamani S. Uversky V. N. Fink A. L. (2004). Rifampicin inhibits alpha-synuclein fibrillation and disaggregates fibrils. *Chem. Biol.* 11 1513–1521. 10.1016/j.chembiol.2004.08.025 15556002

[B247] Li J. Y. Englund E. Holton J. L. Soulet D. Hagell P. Lees A. J. (2008). Lewy bodies in grafted neurons in subjects with Parkinson’s disease suggest host-to-graft disease propagation. *Nat. Med.* 14 501–503. 10.1038/nm1746 18391963

[B248] Li J. Y. Englund E. Widner H. Rehncrona S. Bjorklund A. Lindvall O. (2010). Characterization of Lewy body pathology in 12- and 16-year-old intrastriatal mesencephalic grafts surviving in a patient with Parkinson’s disease. *Mov. Disord.* 25 1091–1096. 10.1002/mds.23012 20198645

[B249] Lin A. Zheng W. He Y. Tang W. Wei X. He R. (2018). Gut microbiota in patients with Parkinson’s disease in southern China. *Parkinsonism Relat. Disord.* 53 82–88. 10.1016/j.parkreldis.2018.05.007 29776865

[B250] Lin D. Liang Y. Jing X. Chen Y. Lei M. Zeng Z. (2018). Microarray analysis of an synthetic alpha-synuclein induced cellular model reveals the expression profile of long non-coding RNA in Parkinson’s disease. *Brain Res.* 1678 384–396. 10.1016/j.brainres.2017.11.007 29137975

[B251] Lin T. K. Chen S. D. Chuang Y. C. Lin H. Y. Huang C. R. Chuang J. H. (2014). Resveratrol partially prevents rotenone-induced neurotoxicity in dopaminergic SH-SY5Y cells through induction of heme oxygenase-1 dependent autophagy. *Int. J. Mol. Sci.* 15 1625–1646. 10.3390/ijms15011625 24451142 PMC3907890

[B252] Lindersson E. Lundvig D. Petersen C. Madsen P. Nyengaard J. R. Hojrup P. (2005). p25alpha Stimulates alpha-synuclein aggregation and is co-localized with aggregated alpha-synuclein in alpha-synucleinopathies. *J. Biol. Chem.* 280 5703–5715. 10.1074/jbc.M410409200 15590652

[B253] Liu C. W. Corboy M. J. DeMartino G. N. Thomas P. J. (2003). Endoproteolytic activity of the proteasome. *Science* 299 408–411. 10.1126/science.1079293 12481023 PMC3516294

[B254] Liu G. Chen M. Mi N. Yang W. Li X. Wang P. (2015). Increased oligomerization and phosphorylation of alpha-synuclein are associated with decreased activity of glucocerebrosidase and protein phosphatase 2A in aging monkey brains. *Neurobiol. Aging* 36 2649–2659. 10.1016/j.neurobiolaging.2015.06.004 26149921

[B255] Liu K. Shi N. Sun Y. Zhang T. Sun X. (2013). Therapeutic effects of rapamycin on MPTP-induced Parkinsonism in mice. *Neurochem. Res.* 38 201–207. 10.1007/s11064-012-0909-8 23117422

[B256] Lo Bianco C. Ridet J. L. Schneider B. L. Deglon N. Aebischer P. (2002). alpha -Synucleinopathy and selective dopaminergic neuron loss in a rat lentiviral-based model of Parkinson’s disease. *Proc. Natl. Acad. Sci. U.S.A.* 99 10813–10818. 10.1073/pnas.152339799 12122208 PMC125054

[B257] Logan T. Bendor J. Toupin C. Thorn K. Edwards R. H. (2017). alpha-Synuclein promotes dilation of the exocytotic fusion pore. *Nat. Neurosci.* 20 681–689. 10.1038/nn.4529 28288128 PMC5404982

[B258] Longhena F. Faustini G. Spillantini M. G. Bellucci A. (2019). Living in promiscuity: the multiple partners of Alpha-synuclein at the synapse in physiology and pathology. *Int. J. Mol. Sci.* 20:141. 10.3390/ijms20010141 30609739 PMC6337145

[B259] Loria F. Vargas J. Y. Bousset L. Syan S. Salles A. Melki R. (2017). alpha-Synuclein transfer between neurons and astrocytes indicates that astrocytes play a role in degradation rather than in spreading. *Acta Neuropathol.* 134 789–808. 10.1007/s00401-017-1746-2 28725967

[B260] Low P. A. Robertson D. Gilman S. Kaufmann H. Singer W. Biaggioni I. (2014). Efficacy and safety of rifampicin for multiple system atrophy: a randomised, double-blind, placebo-controlled trial. *Parkinsonism Relat. Disord.* 13 268–275. 10.1016/S1474-4422(13)70301-6 PMC4030757 24507091

[B261] Lu M. Su C. Qiao C. Bian Y. Ding J. Hu G. (2016). Metformin prevents dopaminergic neuron death in MPTP/P-induced mouse model of Parkinson’s disease via autophagy and mitochondrial ROS CLEARANCE. *Int. J. Neuropsychopharmacol.* 19:pyw047. 10.1093/ijnp/pyw047 27207919 PMC5043649

[B262] Luk K. C. Kehm V. Carroll J. Zhang B. O’Brien P. Trojanowski J. Q. (2012a). Pathological alpha-synuclein transmission initiates Parkinson-like neurodegeneration in nontransgenic mice. *Science* 338 949–953. 10.1126/science.1227157 23161999 PMC3552321

[B263] Luk K. C. Kehm V. M. Zhang B. O’Brien P. Trojanowski J. Q. Lee V. M. (2012b). Intracerebral inoculation of pathological alpha-synuclein initiates a rapidly progressive neurodegenerative alpha-synucleinopathy in mice. *J. Exp. Med.* 209 975–986. 10.1084/jem.20112457 22508839 PMC3348112

[B264] Luk K. C. Song C. O’Brien P. Stieber A. Branch J. R. Brunden K. R. (2009). Exogenous alpha-synuclein fibrils seed the formation of Lewy body-like intracellular inclusions in cultured cells. *Proc. Natl. Acad. Sci. U.S.A.* 106 20051–20056. 10.1073/pnas.0908005106 19892735 PMC2785290

[B265] Luna E. Decker S. C. Riddle D. M. Caputo A. Zhang B. Cole T. (2018). Differential alpha-synuclein expression contributes to selective vulnerability of hippocampal neuron subpopulations to fibril-induced toxicity. *Acta Neuropathol.* 135 855–875. 10.1007/s00401-018-1829-8 29502200 PMC5955788

[B266] Ly T. Julian R. R. (2008). Protein-metal interactions of calmodulin and alpha-synuclein monitored by selective noncovalent adduct protein probing mass spectrometry. *J. Am. Soc. Mass Spectrom.* 19 1663–1672. 10.1016/j.jasms.2008.07.006 18691903

[B267] Lynch S. M. Zhou C. Messer A. (2008). An scFv intrabody against the nonamyloid component of alpha-synuclein reduces intracellular aggregation and toxicity. *J. Mol. Biol.* 377 136–147. 10.1016/j.jmb.2007.11.096 18237741 PMC2359154

[B268] Ma L. Yang C. Zhang X. Li Y. Wang S. Zheng L. (2018). C-terminal truncation exacerbates the aggregation and cytotoxicity of alpha-Synuclein: a vicious cycle in Parkinson’s disease. *Biochim. Biophys. Acta Mol. Basis Dis.* 1864 3714–3725. 10.1016/j.bbadis.2018.10.003 30290273

[B269] Ma M. R. Hu Z. W. Zhao Y. F. Chen Y. X. Li Y. M. (2016). Phosphorylation induces distinct alpha-synuclein strain formation. *Sci. Rep.* 6:37130. 10.1038/srep37130 27853185 PMC5112567

[B270] Mahul-Mellier A. L. Fauvet B. Gysbers A. Dikiy I. Oueslati A. Georgeon S. (2014). c-Abl phosphorylates alpha-synuclein and regulates its degradation: implication for alpha-synuclein clearance and contribution to the pathogenesis of Parkinson’s disease. *Hum. Mol. Genet.* 23 2858–2879. 10.1093/hmg/ddt674 24412932 PMC4014189

[B271] Mak S. K. McCormack A. L. Manning-Bog A. B. Cuervo A. M. Di Monte D. A. (2010). Lysosomal degradation of alpha-synuclein *in vivo*. *J. Biol. Chem.* 285 13621–13629. 10.1074/jbc.M109.074617 20200163 PMC2859524

[B272] Malagelada C. Jin Z. H. Jackson-Lewis V. Przedborski S. Greene L. A. (2010). Rapamycin protects against neuron death in *in vitro* and *in vivo* models of Parkinson’s disease. *J. Neurosci.* 30 1166–1175. 10.1523/JNEUROSCI.3944-09.2010 20089925 PMC2880868

[B273] Malerba M. Ragnoli B. (2008). Ambroxol in the 21st century: pharmacological and clinical update. *Expert Opin. Drug Metab. Toxicol.* 4 1119–1129. 10.1517/17425255.4.8.1119 18680446

[B274] Mandler M. Valera E. Rockenstein E. Mante M. Weninger H. Patrick C. (2015). Active immunization against alpha-synuclein ameliorates the degenerative pathology and prevents demyelination in a model of multiple system atrophy. *Mol. Neurodegener.* 10:10. 10.1186/s13024-015-0008-9 25886309 PMC4411775

[B275] Mandler M. Valera E. Rockenstein E. Weninger H. Patrick C. Adame A. (2014). Next-generation active immunization approach for synucleinopathies: implications for Parkinson’s disease clinical trials. *Acta Neuropathol.* 127 861–879. 10.1007/s00401-014-1256-4 24525765 PMC4034750

[B276] Manfredsson F. P. Luk K. C. Benskey M. J. Gezer A. Garcia J. Kuhn N. C. (2018). Induction of alpha-synuclein pathology in the enteric nervous system of the rat and non-human primate results in gastrointestinal dysmotility and transient CNS pathology. *Neurobiol. Dis.* 112 106–118. 10.1016/j.nbd.2018.01.008 29341898 PMC5890443

[B277] Mao X. Ou M. T. Karuppagounder S. S. Kam T. I. Yin X. Xiong Y. (2016). Pathological alpha-synuclein transmission initiated by binding lymphocyte-activation gene 3. *Science* 353:aah3374. 10.1126/science.aah3374 27708076 PMC5510615

[B278] Maroteaux L. Campanelli J. T. Scheller R. H. (1988). Synuclein: a neuron-specific protein localized to the nucleus and presynaptic nerve terminal. *J. Neurosci.* 8 2804–2815. 10.1523/jneurosci.08-08-02804.1988 3411354 PMC6569395

[B279] Martin-Clemente B. Alvarez-Castelao B. Mayo I. Sierra A. B. Diaz V. Milan M. (2004). alpha-Synuclein expression levels do not significantly affect proteasome function and expression in mice and stably transfected PC12 cell lines. *J. Biol. Chem.* 279 52984–52990. 10.1074/jbc.M409028200 15466467

[B280] Martinez-Vicente M. Talloczy Z. Kaushik S. Massey A. C. Mazzulli J. Mosharov E. V. (2008). Dopamine-modified alpha-synuclein blocks chaperone-mediated autophagy. *J. Clin. Invest.* 118 777–788. 10.1172/JCI32806 18172548 PMC2157565

[B281] Masliah E. Rockenstein E. Adame A. Alford M. Crews L. Hashimoto M. (2005). Effects of alpha-synuclein immunization in a mouse model of Parkinson’s disease. *Neuron* 46 857–868. 10.1016/j.neuron.2005.05.010 15953415

[B282] Masliah E. Rockenstein E. Mante M. Crews L. Spencer B. Adame A. (2011). Passive immunization reduces behavioral and neuropathological deficits in an alpha-synuclein transgenic model of Lewy body disease. *PLoS One* 6:e19338. 10.1371/journal.pone.0019338 21559417 PMC3084838

[B283] Masliah E. Rockenstein E. Veinbergs I. Sagara Y. Mallory M. Hashimoto M. (2001). beta-amyloid peptides enhance alpha-synuclein accumulation and neuronal deficits in a transgenic mouse model linking Alzheimer’s disease and Parkinson’s disease. *Proc. Natl. Acad. Sci. U.S.A.* 98 12245–12250. 10.1073/pnas.211412398 11572944 PMC59799

[B284] Masuda M. Suzuki N. Taniguchi S. Oikawa T. Nonaka T. Iwatsubo T. (2006). Small molecule inhibitors of alpha-synuclein filament assembly. *Biochemistry* 45 6085–6094. 10.1021/bi0600749 16681381

[B285] Masuda-Suzukake M. Nonaka T. Hosokawa M. Kubo M. Shimozawa A. Akiyama H. (2014). Pathological alpha-synuclein propagates through neural networks. *Acta Neuropathol. Commun.* 2:88. 10.1186/s40478-014-0088-8 25095794 PMC4147188

[B286] Masuda-Suzukake M. Nonaka T. Hosokawa M. Oikawa T. Arai T. Akiyama H. (2013). Prion-like spreading of pathological alpha-synuclein in brain. *Brain* 136(Pt 4), 1128–1138. 10.1093/brain/awt037 23466394 PMC3613715

[B287] Mata I. F. Samii A. Schneer S. H. Roberts J. W. Griffith A. Leis B. C. (2008). Glucocerebrosidase gene mutations: a risk factor for Lewy body disorders. *Arch. Neurol.* 65 379–382. 10.1001/archneurol.2007.68 18332251 PMC2826203

[B288] Mavroeidi P. Arvanitaki F. Karakitsou A. K. Vetsi M. Kloukina I. Zweckstetter M. (2019). Endogenous oligodendroglial alpha-synuclein and TPPP/p25alpha orchestrate alpha-synuclein pathology in experimental multiple system atrophy models. *Acta Neuropathol.* 138 415–441. 10.1007/s00401-019-02014-y 31011860 PMC7289399

[B289] Mazzulli J. R. Xu Y. H. Sun Y. Knight A. L. McLean P. J. Caldwell G. A. (2011). Gaucher disease glucocerebrosidase and alpha-synuclein form a bidirectional pathogenic loop in synucleinopathies. *Cell* 146 37–52. 10.1016/j.cell.2011.06.001 21700325 PMC3132082

[B290] McCormack A. L. Mak S. K. Henderson J. M. Bumcrot D. Farrer M. J. Di Monte D. A. (2010). Alpha-synuclein suppression by targeted small interfering RNA in the primate substantia nigra. *PLoS One* 5:e12122. 10.1371/journal.pone.0012122 20711464 PMC2920329

[B291] McNeill A. Magalhaes J. Shen C. Chau K. Y. Hughes D. Mehta A. (2014). Ambroxol improves lysosomal biochemistry in glucocerebrosidase mutation-linked Parkinson disease cells. *Brain* 137(Pt 5), 1481–1495. 10.1093/brain/awu020 24574503 PMC3999713

[B292] Migdalska-Richards A. Daly L. Bezard E. Schapira A. H. (2016). Ambroxol effects in glucocerebrosidase and alpha-synuclein transgenic mice. *Ann. Neurol.* 80 766–775. 10.1002/ana.24790 27859541 PMC5132106

[B293] Migdalska-Richards A. Ko W. K. D. Li Q. Bezard E. Schapira A. H. V. (2017). Oral ambroxol increases brain glucocerebrosidase activity in a nonhuman primate. *Synapse* 71:e21967. 10.1002/syn.21967 28295625 PMC5485051

[B294] Minakaki G. Menges S. Kittel A. Emmanouilidou E. Schaeffner I. Barkovits K. (2018). Autophagy inhibition promotes SNCA/alpha-synuclein release and transfer via extracellular vesicles with a hybrid autophagosome-exosome-like phenotype. *Autophagy* 14 98–119. 10.1080/15548627.2017.1395992 29198173 PMC5846507

[B295] Mittal S. Bjornevik K. Im D. S. Flierl A. Dong X. Locascio J. J. (2017). beta2-Adrenoreceptor is a regulator of the alpha-synuclein gene driving risk of Parkinson’s disease. *Science* 357 891–898. 10.1126/science.aaf3934 28860381 PMC5761666

[B296] Mogi M. Harada M. Kondo T. Riederer P. Inagaki H. Minami M. (1994a). Interleukin-1 beta, interleukin-6, epidermal growth factor and transforming growth factor-alpha are elevated in the brain from parkinsonian patients. *Neurosci. Lett.* 180 147–150. 10.1016/0304-3940(94)90508-8 7700568

[B297] Mogi M. Harada M. Riederer P. Narabayashi H. Fujita K. Nagatsu T. (1994b). Tumor necrosis factor-alpha (TNF-alpha) increases both in the brain and in the cerebrospinal fluid from parkinsonian patients. *Neurosci. Lett.* 165 208–210. 10.1016/0304-3940(94)90746-3 8015728

[B298] Monzio Compagnoni G. Di Fonzo A. (2019). Understanding the pathogenesis of multiple system atrophy: state of the art and future perspectives. *Acta Neuropathol. Commun.* 7:113. 10.1186/s40478-019-0730-6 31300049 PMC6624923

[B299] Moore J. S. Gibson P. R. Burgell R. E. (2018). Neuromodulation via interferential electrical stimulation as a novel therapy in gastrointestinal motility disorders. *J. Neurogastroenterol. Motil.* 24 19–29. 10.5056/jnm17071 29291605 PMC5753900

[B300] Mosley R. L. Lu Y. Olson K. E. Machhi J. Yan W. Namminga K. L. (2019). A synthetic agonist to vasoactive intestinal peptide receptor-2 induces regulatory T cell neuroprotective activities in models of Parkinson’s disease. *Front. Cell. Neurosci.* 13:421. 10.3389/fncel.2019.00421 31619964 PMC6759633

[B301] Mougenot A. L. Nicot S. Bencsik A. Morignat E. Verchere J. Lakhdar L. (2012). Prion-like acceleration of a synucleinopathy in a transgenic mouse model. *Neurobiol. Aging* 33 2225–2228. 10.1016/j.neurobiolaging.2011.06.022 21813214

[B302] Mullin S. Smith L. Lee K. D’Souza G. Woodgate P. Elflein J. (2020). Ambroxol for the treatment of patients with Parkinson disease with and without glucocerebrosidase gene mutations: a nonrandomized, noncontrolled trial. *JAMA Neurol.* 77 427–434. 10.1001/jamaneurol.2019.4611 31930374 PMC6990847

[B303] Murphy K. E. Gysbers A. M. Abbott S. K. Spiro A. S. Furuta A. Cooper A. (2015). Lysosomal-associated membrane protein 2 isoforms are differentially affected in early Parkinson’s disease. *Mov. Disord.* 30 1639–1647. 10.1002/mds.26141 25594542

[B304] Murphy K. E. Gysbers A. M. Abbott S. K. Tayebi N. Kim W. S. Sidransky E. (2014). Reduced glucocerebrosidase is associated with increased alpha-synuclein in sporadic Parkinson’s disease. *Brain* 137(Pt 3), 834–848. 10.1093/brain/awt367 24477431 PMC3927701

[B305] Myohanen T. T. Hannula M. J. Van Elzen R. Gerard M. Van Der Veken P. Garcia-Horsman J. A. (2012). A prolyl oligopeptidase inhibitor, KYP-2047, reduces alpha-synuclein protein levels and aggregates in cellular and animal models of Parkinson’s disease. *Br. J. Pharmacol.* 166 1097–1113. 10.1111/j.1476-5381.2012.01846.x 22233220 PMC3417432

[B306] Nagatsu T. Mogi M. Ichinose H. Togari A. (2000). “Cytokines in Parkinson’s disease,” in *Advances in Research on Neurodegeneration*, eds Mizuno Y. Calne D. B. Horowski R. Poewe W. Riederer P. Youdim M. B. H. (Vienna: Springer).

[B307] Nagatsu T. Sawada M. (2005). Inflammatory process in Parkinson’s disease: role for cytokines. *Curr. Pharm. Des.* 11 999–1016. 10.2174/1381612053381620 15777250

[B308] Nakamura K. Mori F. Kon T. Tanji K. Miki Y. Tomiyama M. (2015). Filamentous aggregations of phosphorylated alpha-synuclein in Schwann cells (Schwann cell cytoplasmic inclusions) in multiple system atrophy. *Acta Neuropathol. Commun.* 3:29. 10.1186/s40478-015-0208-0 25990096 PMC4438578

[B309] Nakamura K. Nemani V. M. Azarbal F. Skibinski G. Levy J. M. Egami K. (2011). Direct membrane association drives mitochondrial fission by the Parkinson disease-associated protein alpha-synuclein. *J. Biol. Chem.* 286 20710–20726. 10.1074/jbc.M110.213538 21489994 PMC3121472

[B310] Nakayama K. Suzuki Y. Yazawa I. (2012). Binding of neuronal alpha-synuclein to beta-III tubulin and accumulation in a model of multiple system atrophy. *Biochem. Biophys. Res. Commun.* 417 1170–1175. 10.1016/j.bbrc.2011.12.092 22227187

[B311] Nalls M. A. Pankratz N. Lill C. M. Do C. B. Hernandez D. G. Saad M. (2014). Large-scale meta-analysis of genome-wide association data identifies six new risk loci for Parkinson’s disease. *Nat. Genet.* 46 989–993. 10.1038/ng.3043 25064009 PMC4146673

[B312] Nemani V. M. Lu W. Berge V. Nakamura K. Onoa B. Lee M. K. (2010). Increased expression of alpha-synuclein reduces neurotransmitter release by inhibiting synaptic vesicle reclustering after endocytosis. *Neuron* 65 66–79. 10.1016/j.neuron.2009.12.023 20152114 PMC3119527

[B313] Ng C. H. Guan M. S. Koh C. Ouyang X. Yu F. Tan E. K. (2012). AMP kinase activation mitigates dopaminergic dysfunction and mitochondrial abnormalities in Drosophila models of Parkinson’s disease. *J. Neurosci.* 32 14311–14317. 10.1523/JNEUROSCI.0499-12.2012 23055502 PMC6622371

[B314] Nishie M. Mori F. Fujiwara H. Hasegawa M. Yoshimoto M. Iwatsubo T. (2004). Accumulation of phosphorylated alpha-synuclein in the brain and peripheral ganglia of patients with multiple system atrophy. *Acta Neuropathol.* 107 292–298. 10.1007/s00401-003-0811-1 14722716

[B315] Norris E. H. Giasson B. I. Ischiropoulos H. Lee V. M. (2003). Effects of oxidative and nitrative challenges on alpha-synuclein fibrillogenesis involve distinct mechanisms of protein modifications. *J. Biol. Chem.* 278 27230–27240. 10.1074/jbc.M212436200 12857790

[B316] Obergasteiger J. Frapporti G. Pramstaller P. P. Hicks A. A. Volta M. (2018). A new hypothesis for Parkinson’s disease pathogenesis: GTPase-p38 MAPK signaling and autophagy as convergence points of etiology and genomics. *Mol. Neurodegener.* 13:40. 10.1186/s13024-018-0273-5 30071902 PMC6090926

[B317] Odagiri S. Tanji K. Mori F. Kakita A. Takahashi H. Wakabayashi K. (2012). Autophagic adapter protein NBR1 is localized in Lewy bodies and glial cytoplasmic inclusions and is involved in aggregate formation in alpha-synucleinopathy. *Acta Neuropathol.* 124 173–186. 10.1007/s00401-012-0975-7 22484440

[B318] Okun M. S. (2012). Deep-brain stimulation for Parkinson’s disease. *N. Engl. J. Med.* 367 1529–1538. 10.1056/NEJMct1208070 23075179

[B319] Olson K. E. Bade A. N. Schutt C. R. Dong J. Shandler S. J. Boska M. D. (2016). Manganese-enhanced magnetic resonance imaging for detection of vasoactive intestinal peptide receptor 2 agonist therapy in a model of Parkinson’s disease. *Neurotherapeutics* 13 635–646. 10.1007/s13311-016-0449-z 27329163 PMC4965412

[B320] Olson K. E. Kosloski-Bilek L. M. Anderson K. M. Diggs B. J. Clark B. E. Gledhill J. M. (2015). Selective VIP receptor agonists facilitate immune transformation for dopaminergic neuroprotection in MPTP-intoxicated mice. *J. Neurosci.* 35 16463–16478. 10.1523/JNEUROSCI.2131-15.2015 26674871 PMC4679826

[B321] Onfelt B. Nedvetzki S. Yanagi K. Davis D. M. (2004). Cutting edge: membrane nanotubes connect immune cells. *J. Immunol.* 173 1511–1513. 10.4049/jimmunol.173.3.1511 15265877

[B322] Orme T. Guerreiro R. Bras J. (2018). The genetics of dementia with Lewy bodies: current understanding and future directions. *Curr. Neurol. Neurosci. Rep.* 18:67. 10.1007/s11910-018-0874-y 30097731 PMC6097049

[B323] Osellame L. D. Rahim A. A. Hargreaves I. P. Gegg M. E. Richard-Londt A. Brandner S. (2013). Mitochondria and quality control defects in a mouse model of Gaucher disease–links to Parkinson’s disease. *Cell Metab.* 17 941–953. 10.1016/j.cmet.2013.04.014 23707074 PMC3678026

[B324] Oueslati A. (2016). Implication of Alpha-synuclein phosphorylation at S129 in synucleinopathies: What have we learned in the last decade? *J. Parkinsons Dis.* 6 39–51. 10.3233/JPD-160779 27003784 PMC4927808

[B325] Oueslati A. Fournier M. Lashuel H. A. (2010). Role of post-translational modifications in modulating the structure, function and toxicity of alpha-synuclein: implications for Parkinson’s disease pathogenesis and therapies. *Prog. Brain Res.* 183 115–145. 10.1016/S0079-6123(10)83007-9 20696318

[B326] Paillusson S. Clairembault T. Biraud M. Neunlist M. Derkinderen P. (2013). Activity-dependent secretion of alpha-synuclein by enteric neurons. *J. Neurochem.* 125 512–517. 10.1111/jnc.12131 23278133

[B327] Paleologou K. E. Oueslati A. Shakked G. Rospigliosi C. C. Kim H. Y. Lamberto G. R. (2010). Phosphorylation at S87 is enhanced in synucleinopathies, inhibits alpha-synuclein oligomerization, and influences synuclein-membrane interactions. *J. Neurosci.* 30 3184–3198. 10.1523/JNEUROSCI.5922-09.2010 20203178 PMC2947449

[B328] Paleologou K. E. Schmid A. W. Rospigliosi C. C. Kim H. Y. Lamberto G. R. Fredenburg R. A. (2008). Phosphorylation at Ser-129 but not the phosphomimics S129E/D inhibits the fibrillation of alpha-synuclein. *J. Biol. Chem.* 283 16895–16905. 10.1074/jbc.M800747200 18343814 PMC2423264

[B329] Pandey N. Strider J. Nolan W. C. Yan S. X. Galvin J. E. (2008). Curcumin inhibits aggregation of alpha-synuclein. *Acta Neuropathol.* 115 479–489. 10.1007/s00401-007-0332-4 18189141

[B330] Park J. Y. Kim K. S. Lee S. B. Ryu J. S. Chung K. C. Choo Y. K. (2009). On the mechanism of internalization of alpha-synuclein into microglia: roles of ganglioside GM1 and lipid raft. *J. Neurochem.* 110 400–411. 10.1111/j.1471-4159.2009.06150.x 19457104

[B331] Park S. M. Jung H. Y. Kim T. D. Park J. H. Yang C. H. Kim J. (2002). Distinct roles of the N-terminal-binding domain and the C-terminal-solubilizing domain of alpha-synuclein, a molecular chaperone. *J. Biol. Chem.* 277 28512–28520. 10.1074/jbc.M111971200 12032141

[B332] Pasanen P. Myllykangas L. Siitonen M. Raunio A. Kaakkola S. Lyytinen J. (2014). Novel alpha-synuclein mutation A53E associated with atypical multiple system atrophy and Parkinson’s disease-type pathology. *Neurobiol. Aging* 35 2180.e1–2180.e5. 10.1016/j.neurobiolaging.2014.03.024 24746362

[B333] Patil S. P. Jain P. D. Ghumatkar P. J. Tambe R. Sathaye S. (2014). Neuroprotective effect of metformin in MPTP-induced Parkinson’s disease in mice. *Neuroscience* 277 747–754. 10.1016/j.neuroscience.2014.07.046 25108167

[B334] Paumier K. L. Luk K. C. Manfredsson F. P. Kanaan N. M. Lipton J. W. Collier T. J. (2015). Intrastriatal injection of pre-formed mouse alpha-synuclein fibrils into rats triggers alpha-synuclein pathology and bilateral nigrostriatal degeneration. *Neurobiol. Dis.* 82 185–199. 10.1016/j.nbd.2015.06.003 26093169 PMC4640952

[B335] Peelaerts W. Bousset L. Van der Perren A. Moskalyuk A. Pulizzi R. Giugliano M. (2015). alpha-Synuclein strains cause distinct synucleinopathies after local and systemic administration. *Nature* 522 340–344. 10.1038/nature14547 26061766

[B336] Peng C. Gathagan R. J. Covell D. J. Medellin C. Stieber A. Robinson J. L. (2018a). Cellular milieu imparts distinct pathological alpha-synuclein strains in alpha-synucleinopathies. *Nature* 557 558–563. 10.1038/s41586-018-0104-4 29743672 PMC5970994

[B337] Peng C. Gathagan R. J. Lee V. M. (2018b). Distinct alpha-Synuclein strains and implications for heterogeneity among alpha-Synucleinopathies. *Neurobiol. Dis.* 109(Pt B), 209–218. 10.1016/j.nbd.2017.07.018 28751258 PMC5735026

[B338] Peng X. Tehranian R. Dietrich P. Stefanis L. Perez R. G. (2005). Alpha-synuclein activation of protein phosphatase 2A reduces tyrosine hydroxylase phosphorylation in dopaminergic cells. *J. Cell Sci.* 118(Pt 15), 3523–3530. 10.1242/jcs.02481 16030137

[B339] Pieri L. Madiona K. Melki R. (2016). Structural and functional properties of prefibrillar alpha-synuclein oligomers. *Sci. Rep.* 6:24526. 10.1038/srep24526 27075649 PMC4830946

[B340] Pirc K. Ulrih N. P. (2015). alpha-Synuclein interactions with phospholipid model membranes: key roles for electrostatic interactions and lipid-bilayer structure. *Biochim. Biophys. Acta* 1848(10 Pt A), 2002–2012. 10.1016/j.bbamem.2015.06.021 26119565

[B341] Pletnikova O. West N. Lee M. K. Rudow G. L. Skolasky R. L. Dawson T. M. (2005). Abeta deposition is associated with enhanced cortical alpha-synuclein lesions in Lewy body diseases. *Neurobiol. Aging* 26 1183–1192. 10.1016/j.neurobiolaging.2004.10.006 15917102

[B342] Politis M. Su P. Piccini P. (2012). Imaging of microglia in patients with neurodegenerative disorders. *Front. Pharmacol.* 3:96. 10.3389/fphar.2012.00096 22661951 PMC3361961

[B343] Polymeropoulos M. H. Lavedan C. Leroy E. Ide S. E. Dehejia A. Dutra A. (1997). Mutation in the alpha-synuclein gene identified in families with Parkinson’s disease. *Science* 276 2045–2047. 10.1126/science.276.5321.2045 9197268

[B344] Pouclet H. Lebouvier T. Coron E. Des Varannes S. B. Neunlist M. Derkinderen P. (2012). A comparison between colonic submucosa and mucosa to detect Lewy pathology in Parkinson’s disease. *Neurogastroenterol. Motil.* 24 e202–e205. 10.1111/j.1365-2982.2012.01887.x 22292943

[B345] Prabhudesai S. Sinha S. Attar A. Kotagiri A. Fitzmaurice A. G. Lakshmanan R. (2012). A novel “molecular tweezer” inhibitor of alpha-synuclein neurotoxicity *in vitro* and *in vivo*. *Neurotherapeutics* 9 464–476. 10.1007/s13311-012-0105-1 22373667 PMC3337029

[B346] Price D. L. Koike M. A. Khan A. Wrasidlo W. Rockenstein E. Masliah E. (2018). The small molecule alpha-synuclein misfolding inhibitor, NPT200-11, produces multiple benefits in an animal model of Parkinson’s disease. *Sci. Rep.* 8:16165. 10.1038/s41598-018-34490-9 30385782 PMC6212487

[B347] Proukakis C. Dudzik C. G. Brier T. MacKay D. S. Cooper J. M. Millhauser G. L. (2013). A novel alpha-synuclein missense mutation in Parkinson disease. *Neurology* 80 1062–1064. 10.1212/WNL.0b013e31828727ba 23427326 PMC3653201

[B348] Prusiner S. B. Woerman A. L. Mordes D. A. Watts J. C. Rampersaud R. Berry D. B. (2015). Evidence for alpha-synuclein prions causing multiple system atrophy in humans with parkinsonism. *Proc. Natl. Acad. Sci. U.S.A.* 112 E5308–E5317. 10.1073/pnas.1514475112 26324905 PMC4586853

[B349] Pukass K. Goldbaum O. Richter-Landsberg C. (2015). Mitochondrial impairment and oxidative stress compromise autophagosomal degradation of alpha-synuclein in oligodendroglial cells. *J. Neurochem.* 135 194–205. 10.1111/jnc.13256 26212128

[B350] Pukass K. Richter-Landsberg C. (2014). Oxidative stress promotes uptake, accumulation, and oligomerization of extracellular alpha-synuclein in oligodendrocytes. *J. Mol. Neurosci.* 52 339–352. 10.1007/s12031-013-0154-x 24217795

[B351] Ravina B. Putt M. Siderowf A. Farrar J. T. Gillespie M. Crawley A. (2005). Donepezil for dementia in Parkinson’s disease: a randomised, double blind, placebo controlled, crossover study. *J. Neurol. Neurosurg. Psychiatry* 76 934–939. 10.1136/jnnp.2004.050682 15965198 PMC1739697

[B352] Recasens A. Dehay B. Bove J. Carballo-Carbajal I. Dovero S. Perez-Villalba A. (2014). Lewy body extracts from Parkinson disease brains trigger alpha-synuclein pathology and neurodegeneration in mice and monkeys. *Ann. Neurol.* 75 351–362. 10.1002/ana.24066 24243558

[B353] Rey N. L. Petit G. H. Bousset L. Melki R. Brundin P. (2013). Transfer of human alpha-synuclein from the olfactory bulb to interconnected brain regions in mice. *Acta Neuropathol.* 126 555–573. 10.1007/s00401-013-1160-3 23925565 PMC3789892

[B354] Rey N. L. Steiner J. A. Maroof N. Luk K. C. Madaj Z. Trojanowski J. Q. (2016). Widespread transneuronal propagation of alpha-synucleinopathy triggered in olfactory bulb mimics prodromal Parkinson’s disease. *J. Exp. Med.* 213 1759–1778. 10.1084/jem.20160368 27503075 PMC4995088

[B355] Reyes J. F. Rey N. L. Bousset L. Melki R. Brundin P. Angot E. (2014). Alpha-synuclein transfers from neurons to oligodendrocytes. *Glia* 62 387–398. 10.1002/glia.22611 24382629

[B356] Reyes J. F. Sackmann C. Hoffmann A. Svenningsson P. Winkler J. Ingelsson M. (2019). Binding of alpha-synuclein oligomers to Cx32 facilitates protein uptake and transfer in neurons and oligodendrocytes. *Acta Neuropathol.* 138 23–47. 10.1007/s00401-019-02007-x 30976973 PMC6570706

[B357] Reynolds A. D. Stone D. K. Hutter J. A. Benner E. J. Mosley R. L. Gendelman H. E. (2010). Regulatory T cells attenuate Th17 cell-mediated nigrostriatal dopaminergic neurodegeneration in a model of Parkinson’s disease. *J. Immunol.* 184 2261–2271. 10.4049/jimmunol.0901852 20118279 PMC2824790

[B358] Rice T. W. Wheeler A. P. Bernard G. R. Vincent J. L. Angus D. C. Aikawa N. (2010). A randomized, double-blind, placebo-controlled trial of TAK-242 for the treatment of severe sepsis. *Crit. Care Med.* 38 1685–1694. 10.1097/CCM.0b013e3181e7c5c9 20562702

[B359] Richter F. Subramaniam S. R. Magen I. Lee P. Hayes J. Attar A. (2017). A molecular Tweezer ameliorates motor deficits in mice overexpressing alpha-Synuclein. *Neurotherapeutics* 14 1107–1119. 10.1007/s13311-017-0544-9 28585223 PMC5722755

[B360] Rideout H. J. Larsen K. E. Sulzer D. Stefanis L. (2001). Proteasomal inhibition leads to formation of ubiquitin/alpha-synuclein-immunoreactive inclusions in PC12 cells. *J. Neurochem.* 78 899–908. 10.1046/j.1471-4159.2001.00474.x 11520910

[B361] Rideout H. J. Stefanis L. (2002). Proteasomal inhibition-induced inclusion formation and death in cortical neurons require transcription and ubiquitination. *Mol. Cell. Neurosci.* 21 223–238. 10.1006/mcne.2002.1173 12401444

[B362] Rocha E. M. De Miranda B. Sanders L. H. (2018). Alpha-synuclein: pathology, mitochondrial dysfunction and neuroinflammation in Parkinson’s disease. *Neurobiol. Dis.* 109(Pt B), 249–257. 10.1016/j.nbd.2017.04.004 28400134

[B363] Rocha E. M. Smith G. A. Park E. Cao H. Graham A. R. Brown E. (2015). Sustained systemic glucocerebrosidase inhibition induces brain alpha-synuclein aggregation, microglia and complement C1q activation in Mice. *Antioxid. Redox Signal.* 23 550–564. 10.1089/ars.2015.6307 26094487 PMC4544823

[B364] Rockenstein E. Ostroff G. Dikengil F. Rus F. Mante M. Florio J. (2018). Combined active humoral and cellular immunization approaches for the treatment of synucleinopathies. *J. Neurosci.* 38 1000–1014. 10.1523/JNEUROSCI.1170-17.2017 29246926 PMC5783958

[B365] Rockenstein E. Ubhi K. Inglis C. Mante M. Patrick C. Adame A. (2012). Neuronal to oligodendroglial alpha-synuclein redistribution in a double transgenic model of multiple system atrophy. *Neuroreport* 23 259–264. 10.1097/WNR.0b013e3283509842 22314685 PMC3289254

[B366] Rodriguez J. A. Ivanova M. I. Sawaya M. R. Cascio D. Reyes F. E. Shi D. (2015). Structure of the toxic core of alpha-synuclein from invisible crystals. *Nature* 525 486–490. 10.1038/nature15368 26352473 PMC4791177

[B367] Roodveldt C. Labrador-Garrido A. Gonzalez-Rey E. Lachaud C. C. Guilliams T. Fernandez-Montesinos R. (2013). Preconditioning of microglia by alpha-synuclein strongly affects the response induced by toll-like receptor (TLR) stimulation. *PLoS One* 8:e79160. 10.1371/journal.pone.0079160 24236103 PMC3827304

[B368] Rospigliosi C. C. McClendon S. Schmid A. W. Ramlall T. F. Barre P. Lashuel H. A. (2009). E46K Parkinson’s-linked mutation enhances C-terminal-to-N-terminal contacts in alpha-synuclein. *J. Mol. Biol.* 388 1022–1032. 10.1016/j.jmb.2009.03.065 19345692 PMC2719283

[B369] Rott R. Szargel R. Haskin J. Bandopadhyay R. Lees A. J. Shani V. (2011). alpha-Synuclein fate is determined by USP9X-regulated monoubiquitination. *Proc. Natl. Acad. Sci. U.S.A.* 108 18666–18671. 10.1073/pnas.1105725108 22065755 PMC3219120

[B370] Sacino A. N. Brooks M. McGarvey N. H. McKinney A. B. Thomas M. A. Levites Y. (2013a). Induction of CNS alpha-synuclein pathology by fibrillar and non-amyloidogenic recombinant alpha-synuclein. *Acta Neuropathol. Commun.* 1:38. 10.1186/2051-5960-1-38 24252149 PMC3893388

[B371] Sacino A. N. Brooks M. McKinney A. B. Thomas M. A. Shaw G. Golde T. E. (2014a). Brain injection of alpha-synuclein induces multiple proteinopathies, gliosis, and a neuronal injury marker. *J. Neurosci.* 34 12368–12378. 10.1523/JNEUROSCI.2102-14.2014 25209277 PMC6615494

[B372] Sacino A. N. Brooks M. Thomas M. A. McKinney A. B. Lee S. Regenhardt R. W. (2014b). Intramuscular injection of alpha-synuclein induces CNS alpha-synuclein pathology and a rapid-onset motor phenotype in transgenic mice. *Proc. Natl. Acad. Sci. U.S.A.* 111 10732–10737. 10.1073/pnas.1321785111 25002524 PMC4115570

[B373] Sacino A. N. Thomas M. A. Ceballos-Diaz C. Cruz P. E. Rosario A. M. Lewis J. (2013b). Conformational templating of alpha-synuclein aggregates in neuronal-glial cultures. *Mol. Neurodegener.* 8:17. 10.1186/1750-1326-8-17 23714769 PMC3671973

[B374] Sahin C. Lorenzen N. Lemminger L. Christiansen G. Moller I. M. Vesterager L. B. (2017). Antibodies against the C-terminus of alpha-synuclein modulate its fibrillation. *Biophys. Chem.* 220 34–41. 10.1016/j.bpc.2016.11.002 27863716

[B375] Sailer A. Scholz S. W. Nalls M. A. Schulte C. Federoff M. Price T. R. (2016). A genome-wide association study in multiple system atrophy. *Neurology* 87 1591–1598. 10.1212/WNL.0000000000003221 27629089 PMC5067544

[B376] Saito Y. Kawashima A. Ruberu N. N. Fujiwara H. Koyama S. Sawabe M. (2003). Accumulation of phosphorylated alpha-synuclein in aging human brain. *J. Neuropathol. Exp. Neurol.* 62 644–654. 10.1093/jnen/62.6.644 12834109

[B377] Sampson T. R. Debelius J. W. Thron T. Janssen S. Shastri G. G. Ilhan Z. E. (2016). Gut microbiota regulate motor deficits and neuroinflammation in a model of Parkinson’s disease. *Cell* 167 1469–1480.e12. 10.1016/j.cell.2016.11.018 27912057 PMC5718049

[B378] Sanchez-Guajardo V. Febbraro F. Kirik D. Romero-Ramos M. (2010). Microglia acquire distinct activation profiles depending on the degree of alpha-synuclein neuropathology in a rAAV based model of Parkinson’s disease. *PLoS One* 5:e8784. 10.1371/journal.pone.0008784 20098715 PMC2808388

[B379] Sapru M. K. Yates J. W. Hogan S. Jiang L. Halter J. Bohn M. C. (2006). Silencing of human alpha-synuclein *in vitro* and in rat brain using lentiviral-mediated RNAi. *Exp. Neurol.* 198 382–390. 10.1016/j.expneurol.2005.12.024 16455076

[B380] Sardi S. P. Cedarbaum J. M. Brundin P. (2018). Targeted therapies for Parkinson’s disease: from genetics to the clinic. *Mov. Disord.* 33 684–696. 10.1002/mds.27414 29704272 PMC6282975

[B381] Sardi S. P. Clarke J. Kinnecom C. Tamsett T. J. Li L. Stanek L. M. (2011). CNS expression of glucocerebrosidase corrects alpha-synuclein pathology and memory in a mouse model of Gaucher-related synucleinopathy. *Proc. Natl. Acad. Sci. U.S.A.* 108 12101–12106. 10.1073/pnas.1108197108 21730160 PMC3141921

[B382] Sardi S. P. Clarke J. Viel C. Chan M. Tamsett T. J. Treleaven C. M. (2013). Augmenting CNS glucocerebrosidase activity as a therapeutic strategy for parkinsonism and other Gaucher-related synucleinopathies. *Proc. Natl. Acad. Sci. U.S.A.* 110 3537–3542. 10.1073/pnas.1220464110 23297226 PMC3587272

[B383] Sardi S. P. Viel C. Clarke J. Treleaven C. M. Richards A. M. Park H. (2017). Glucosylceramide synthase inhibition alleviates aberrations in synucleinopathy models. *Proc. Natl. Acad. Sci. U.S.A.* 114 2699–2704. 10.1073/pnas.1616152114 28223512 PMC5347608

[B384] Sarkar S. Chigurupati S. Raymick J. Mann D. Bowyer J. F. Schmitt T. (2014). Neuroprotective effect of the chemical chaperone, trehalose in a chronic MPTP-induced Parkinson’s disease mouse model. *Neurotoxicology* 44 250–262. 10.1016/j.neuro.2014.07.006 25064079

[B385] Sarkar S. Davies J. E. Huang Z. Tunnacliffe A. Rubinsztein D. C. (2007). Trehalose, a novel mTOR-independent autophagy enhancer, accelerates the clearance of mutant huntingtin and alpha-synuclein. *J. Biol. Chem.* 282 5641–5652. 10.1074/jbc.M609532200 17182613

[B386] Satake W. Nakabayashi Y. Mizuta I. Hirota Y. Ito C. Kubo M. (2009). Genome-wide association study identifies common variants at four loci as genetic risk factors for Parkinson’s disease. *Nat. Genet.* 41 1303–1307. 10.1038/ng.485 19915576

[B387] Satish Bollimpelli V. Kondapi A. K. (2015). Differential sensitivity of immature and mature ventral mesencephalic neurons to rotenone induced neurotoxicity *in vitro*. *Toxicol In Vitro* 30(1 Pt B), 545–551. 10.1016/j.tiv.2015.09.006 26363201

[B388] Sato H. Arawaka S. Hara S. Fukushima S. Koga K. Koyama S. (2011). Authentically phosphorylated alpha-synuclein at Ser129 accelerates neurodegeneration in a rat model of familial Parkinson’s disease. *J. Neurosci.* 31 16884–16894. 10.1523/JNEUROSCI.3967-11.2011 22090514 PMC6633319

[B389] Saunders J. A. Estes K. A. Kosloski L. M. Allen H. E. Dempsey K. M. Torres-Russotto D. R. (2012). CD4+ regulatory and effector/memory T cell subsets profile motor dysfunction in Parkinson’s disease. *J. Neuroimmune Pharmacol.* 7 927–938. 10.1007/s11481-012-9402-z 23054369 PMC3515774

[B390] Schapira A. H. Gegg M. E. (2013). Glucocerebrosidase in the pathogenesis and treatment of Parkinson disease. *Proc. Natl. Acad. Sci. U.S.A.* 110 3214–3215. 10.1073/pnas.1300822110 23412333 PMC3587236

[B391] Schenk D. B. Koller M. Ness D. K. Griffith S. G. Grundman M. Zago W. (2017). First-in-human assessment of PRX002, an anti-alpha-synuclein monoclonal antibody, in healthy volunteers. *Mov. Disord.* 32 211–218. 10.1002/mds.26878 27886407 PMC5324684

[B392] Scheperjans F. Aho V. Pereira P. A. Koskinen K. Paulin L. Pekkonen E. (2015). Gut microbiota are related to Parkinson’s disease and clinical phenotype. *Mov. Disord.* 30 350–358. 10.1002/mds.26069 25476529

[B393] Scherzer C. R. Grass J. A. Liao Z. Pepivani I. Zheng B. Eklund A. C. (2008). GATA transcription factors directly regulate the Parkinson’s disease-linked gene alpha-synuclein. *Proc. Natl. Acad. Sci. U.S.A.* 105 10907–10912. 10.1073/pnas.0802437105 18669654 PMC2504800

[B394] Schofield D. J. Irving L. Calo L. Bogstedt A. Rees G. Nuccitelli A. (2019). Preclinical development of a high affinity alpha-synuclein antibody, MEDI1341, that can enter the brain, sequester extracellular alpha-synuclein and attenuate alpha-synuclein spreading *in vivo*. *Neurobiol. Dis.* 132:104582. 10.1016/j.nbd.2019.104582 31445162

[B395] Scholz S. W. Houlden H. Schulte C. Sharma M. Li A. Berg D. (2009). SNCA variants are associated with increased risk for multiple system atrophy. *Ann. Neurol.* 65 610–614. 10.1002/ana.21685 19475667 PMC3520128

[B396] Schreiber K. H. Ortiz D. Academia E. C. Anies A. C. Liao C. Y. Kennedy B. K. (2015). Rapamycin-mediated mTORC2 inhibition is determined by the relative expression of FK506-binding proteins. *Aging Cell* 14 265–273. 10.1111/acel.12313 25652038 PMC4364838

[B397] Schwab K. Frahm S. Horsley D. Rickard J. E. Melis V. Goatman E. A. (2017). A protein aggregation inhibitor, Leuco-Methylthioninium Bis(Hydromethanesulfonate), decreases alpha-synuclein inclusions in a transgenic mouse model of synucleinopathy. *Front. Mol. Neurosci.* 10:447. 10.3389/fnmol.2017.00447 29375308 PMC5767730

[B398] Schwarz L. Goldbaum O. Bergmann M. Probst-Cousin S. Richter-Landsberg C. (2012). Involvement of macroautophagy in multiple system atrophy and protein aggregate formation in oligodendrocytes. *J. Mol. Neurosci.* 47 256–266. 10.1007/s12031-012-9733-5 22411133

[B399] Schweighauser M. Bacioglu M. Fritschi S. K. Shimshek D. R. Kahle P. J. Eisele Y. S. (2015). Formaldehyde-fixed brain tissue from spontaneously ill alpha-synuclein transgenic mice induces fatal alpha-synucleinopathy in transgenic hosts. *Acta Neuropathol.* 129 157–159. 10.1007/s00401-014-1360-5 25367384

[B400] Shahaduzzaman M. Nash K. Hudson C. Sharif M. Grimmig B. Lin X. (2015). Anti-human alpha-synuclein N-terminal peptide antibody protects against dopaminergic cell death and ameliorates behavioral deficits in an AAV-alpha-synuclein rat model of Parkinson’s disease. *PLoS One* 10:e0116841. 10.1371/journal.pone.0116841 25658425 PMC4319932

[B401] Shahnawaz M. Mukherjee A. Pritzkow S. Mendez N. Rabadia P. Liu X. (2020). Discriminating alpha-synuclein strains in Parkinson’s disease and multiple system atrophy. *Nature* 578 273–277. 10.1038/s41586-020-1984-7 32025029 PMC7066875

[B402] Shahpasandzadeh H. Popova B. Kleinknecht A. Fraser P. E. Outeiro T. F. Braus G. H. (2014). Interplay between sumoylation and phosphorylation for protection against alpha-synuclein inclusions. *J. Biol. Chem.* 289 31224–31240. 10.1074/jbc.M114.559237 25231978 PMC4223324

[B403] Shaltiel-Karyo R. Davidi D. Frenkel-Pinter M. Ovadia M. Segal D. Gazit E. (2012). Differential inhibition of alpha-synuclein oligomeric and fibrillar assembly in parkinson’s disease model by cinnamon extract. *Biochim. Biophys. Acta* 1820 1628–1635. 10.1016/j.bbagen.2012.04.021 22575665

[B404] Shaltiel-Karyo R. Frenkel-Pinter M. Egoz-Matia N. Frydman-Marom A. Shalev D. E. Segal D. (2010). Inhibiting alpha-synuclein oligomerization by stable cell-penetrating beta-synuclein fragments recovers phenotype of Parkinson’s disease model flies. *PLoS One* 5:e13863. 10.1371/journal.pone.0013863 21085664 PMC2978097

[B405] Shaltiel-Karyo R. Frenkel-Pinter M. Rockenstein E. Patrick C. Levy-Sakin M. Schiller A. (2013). A blood-brain barrier (BBB) disrupter is also a potent alpha-synuclein (alpha-syn) aggregation inhibitor: a novel dual mechanism of mannitol for the treatment of Parkinson disease (PD). *J. Biol. Chem.* 288 17579–17588. 10.1074/jbc.M112.434787 23637226 PMC3682557

[B406] Shrivastava A. N. Redeker V. Fritz N. Pieri L. Almeida L. G. Spolidoro M. (2015). alpha-synuclein assemblies sequester neuronal alpha3-Na+/K+-ATPase and impair Na+ gradient. *EMBO J.* 34 2408–2423. 10.15252/embj.201591397 26323479 PMC4601662

[B407] Shults C. W. Rockenstein E. Crews L. Adame A. Mante M. Larrea G. (2005). Neurological and neurodegenerative alterations in a transgenic mouse model expressing human alpha-synuclein under oligodendrocyte promoter: implications for multiple system atrophy. *J. Neurosci.* 25 10689–10699. 10.1523/JNEUROSCI.3527-05.2005 16291942 PMC6725840

[B408] Silveira C. R. A. MacKinley J. Coleman K. Li Z. Finger E. Bartha R. (2019). Ambroxol as a novel disease-modifying treatment for Parkinson’s disease dementia: protocol for a single-centre, randomized, double-blind, placebo-controlled trial. *BMC Neurol.* 19:20. 10.1186/s12883-019-1252-3 30738426 PMC6368728

[B409] Simon-Sanchez J. Schulte C. Bras J. M. Sharma M. Gibbs J. R. Berg D. (2009). Genome-wide association study reveals genetic risk underlying Parkinson’s disease. *Nat. Genet.* 41 1308–1312. 10.1038/ng.487 19915575 PMC2787725

[B410] Singleton A. B. Farrer M. Johnson J. Singleton A. Hague S. Kachergus J. (2003). alpha-Synuclein locus triplication causes Parkinson’s disease. *Science* 302:841. 10.1126/science.1090278 14593171

[B411] Smith W. W. Margolis R. L. Li X. Troncoso J. C. Lee M. K. Dawson V. L. (2005). Alpha-synuclein phosphorylation enhances eosinophilic cytoplasmic inclusion formation in SH-SY5Y cells. *J. Neurosci.* 25 5544–5552. 10.1523/JNEUROSCI.0482-05.2005 15944382 PMC6724982

[B412] Sode K. Ochiai S. Kobayashi N. Usuzaka E. (2006). Effect of reparation of repeat sequences in the human alpha-synuclein on fibrillation ability. *Int. J. Biol. Sci.* 3 1–7. 10.7150/ijbs.3.1 17200685 PMC1622890

[B413] Song J. X. Sun Y. R. Peluso I. Zeng Y. Yu X. Lu J. H. (2016). A novel curcumin analog binds to and activates TFEB *in vitro* and *in vivo* independent of MTOR inhibition. *Autophagy* 12 1372–1389. 10.1080/15548627.2016.1179404 27172265 PMC4968239

[B414] Spencer B. Emadi S. Desplats P. Eleuteri S. Michael S. Kosberg K. (2014). ESCRT-mediated uptake and degradation of brain-targeted alpha-synuclein single chain antibody attenuates neuronal degeneration *in vivo*. *Mol. Ther.* 22 1753–1767. 10.1038/mt.2014.129 25008355 PMC4428402

[B415] Spillantini M. G. Crowther R. A. Jakes R. Hasegawa M. Goedert M. (1998). alpha-Synuclein in filamentous inclusions of Lewy bodies from Parkinson’s disease and dementia with lewy bodies. *Proc. Natl. Acad. Sci. U.S.A.* 95 6469–6473. 10.1073/pnas.95.11.6469 9600990 PMC27806

[B416] Spillantini M. G. Goedert M. (2000). The alpha-synucleinopathies: Parkinson’s disease, dementia with Lewy bodies, and multiple system atrophy. *Ann. N. Y. Acad. Sci.* 920 16–27. 10.1111/j.1749-6632.2000.tb06900.x 11193145

[B417] Spillantini M. G. Schmidt M. L. Lee V. M. Trojanowski J. Q. Jakes R. Goedert M. (1997). Alpha-synuclein in Lewy bodies. *Nature* 388 839–840. 10.1038/42166 9278044

[B418] Staats K. A. Hernandez S. Schonefeldt S. Bento-Abreu A. Dooley J. Van Damme P. (2013). Rapamycin increases survival in ALS mice lacking mature lymphocytes. *Mol. Neurodegener.* 8:31. 10.1186/1750-1326-8-31 24025516 PMC3847677

[B419] Stefanis L. Larsen K. E. Rideout H. J. Sulzer D. Greene L. A. (2001). Expression of A53T mutant but not wild-type alpha-synuclein in PC12 cells induces alterations of the ubiquitin-dependent degradation system, loss of dopamine release, and autophagic cell death. *J. Neurosci.* 21 9549–9560. 10.1523/jneurosci.21-24-09549.2001 11739566 PMC6763041

[B420] Stefanova N. Fellner L. Reindl M. Masliah E. Poewe W. Wenning G. K. (2011). Toll-like receptor 4 promotes alpha-synuclein clearance and survival of nigral dopaminergic neurons. *Am. J. Pathol.* 179 954–963. 10.1016/j.ajpath.2011.04.013 21801874 PMC3157205

[B421] Stefanova N. Reindl M. Neumann M. Kahle P. J. Poewe W. Wenning G. K. (2007). Microglial activation mediates neurodegeneration related to oligodendroglial alpha-synucleinopathy: implications for multiple system atrophy. *Mov. Disord.* 22 2196–2203. 10.1002/mds.21671 17853477

[B422] Stefanova N. Reindl M. Poewe W. Wenning G. K. (2005). *In vitro* models of multiple system atrophy. *Mov. Disord.* 20(Suppl. 12), S53–S56. 10.1002/mds.20540 16092091

[B423] Stefanova N. Wenning G. K. (2016). Review: multiple system atrophy: emerging targets for interventional therapies. *Neuropathol. Appl. Neurobiol.* 42 20–32. 10.1111/nan.12304 26785838 PMC4788141

[B424] Stemberger S. Poewe W. Wenning G. K. Stefanova N. (2010). Targeted overexpression of human alpha-synuclein in oligodendroglia induces lesions linked to MSA-like progressive autonomic failure. *Exp. Neurol.* 224 459–464. 10.1016/j.expneurol.2010.05.008 20493840 PMC2913120

[B425] Strohaker T. Jung B. C. Liou S. H. Fernandez C. O. Riedel D. Becker S. (2019). Structural heterogeneity of alpha-synuclein fibrils amplified from patient brain extracts. *Nat. Commun.* 10:5535. 10.1038/s41467-019-13564-w 31797870 PMC6893031

[B426] Stuendl A. Kunadt M. Kruse N. Bartels C. Moebius W. Danzer K. M. (2016). Induction of alpha-synuclein aggregate formation by CSF exosomes from patients with Parkinson’s disease and dementia with Lewy bodies. *Brain* 139(Pt 2), 481–494. 10.1093/brain/awv346 26647156 PMC4805087

[B427] Su X. Maguire-Zeiss K. A. Giuliano R. Prifti L. Venkatesh K. Federoff H. J. (2008). Synuclein activates microglia in a model of Parkinson’s disease. *Neurobiol. Aging* 29 1690–1701. 10.1016/j.neurobiolaging.2007.04.006 17537546 PMC2621109

[B428] Sung J. Y. Kim J. Paik S. R. Park J. H. Ahn Y. S. Chung K. C. (2001). Induction of neuronal cell death by Rab5A-dependent endocytosis of alpha-synuclein. *J. Biol. Chem.* 276 27441–27448. 10.1074/jbc.M101318200 11316809

[B429] Surgucheva I. Gunewardena S. Rao H. S. Surguchov A. (2013). Cell-specific post-transcriptional regulation of gamma-synuclein gene by micro-RNAs. *PLoS One* 8:e73786. 10.1371/journal.pone.0073786 24040069 PMC3770685

[B430] Svensson E. Horvath-Puho E. Thomsen R. W. Djurhuus J. C. Pedersen L. Borghammer P. (2015). Vagotomy and subsequent risk of Parkinson’s disease. *Ann. Neurol.* 78 522–529. 10.1002/ana.24448 26031848

[B431] Swant J. Goodwin J. S. North A. Ali A. A. Gamble-George J. Chirwa S. (2011). alpha-Synuclein stimulates a dopamine transporter-dependent chloride current and modulates the activity of the transporter. *J. Biol. Chem.* 286 43933–43943. 10.1074/jbc.M111.241232 21990355 PMC3243541

[B432] Tan S. Yu C. Y. Sim Z. W. Low Z. S. Lee B. See F. (2019). Pomegranate activates TFEB to promote autophagy-lysosomal fitness and mitophagy. *Sci. Rep.* 9:727. 10.1038/s41598-018-37400-1 30679718 PMC6346015

[B433] Tanaka Y. Engelender S. Igarashi S. Rao R. K. Wanner T. Tanzi R. E. (2001). Inducible expression of mutant alpha-synuclein decreases proteasome activity and increases sensitivity to mitochondria-dependent apoptosis. *Hum. Mol. Genet.* 10 919–926. 10.1093/hmg/10.9.919 11309365

[B434] Tang Y. Le W. (2016). Differential roles of M1 and M2 microglia in neurodegenerative diseases. *Mol. Neurobiol.* 53 1181–1194. 10.1007/s12035-014-9070-5 25598354

[B435] Tanji K. Miki Y. Maruyama A. Mimura J. Matsumiya T. Mori F. (2015). Trehalose intake induces chaperone molecules along with autophagy in a mouse model of Lewy body disease. *Biochem. Biophys. Res. Commun.* 465 746–752. 10.1016/j.bbrc.2015.08.076 26299928

[B436] Tanji K. Odagiri S. Maruyama A. Mori F. Kakita A. Takahashi H. (2013). Alteration of autophagosomal proteins in the brain of multiple system atrophy. *Neurobiol. Dis.* 49 190–198. 10.1016/j.nbd.2012.08.017 22959883

[B437] Tansey M. G. Romero-Ramos M. (2019). Immune system responses in Parkinson’s disease: early and dynamic. *Eur. J. Neurosci.* 49 364–383. 10.1111/ejn.14290 30474172 PMC6391192

[B438] Teki S. Griffiths T. D. (2016). Brain bases of working memory for time intervals in rhythmic sequences. *Front. Neurosci.* 10:239. 10.3389/fnins.2016.00239 27313506 PMC4888525

[B439] Tenreiro S. Reimao-Pinto M. M. Antas P. Rino J. Wawrzycka D. Macedo D. (2014). Phosphorylation modulates clearance of alpha-synuclein inclusions in a yeast model of Parkinson’s disease. *PLoS Genet.* 10:e1004302. 10.1371/journal.pgen.1004302 24810576 PMC4014446

[B440] Terada M. Suzuki G. Nonaka T. Kametani F. Tamaoka A. Hasegawa M. (2018). The effect of truncation on prion-like properties of alpha-synuclein. *J. Biol. Chem.* 293 13910–13920. 10.1074/jbc.RA118.001862 30030380 PMC6130941

[B441] Thayanidhi N. Helm J. R. Nycz D. C. Bentley M. Liang Y. Hay J. C. (2010). Alpha-synuclein delays endoplasmic reticulum (ER)-to-Golgi transport in mammalian cells by antagonizing ER/Golgi SNAREs. *Mol. Biol. Cell* 21 1850–1863. 10.1091/mbc.E09-09-0801 20392839 PMC2877643

[B442] Tofaris G. K. Layfield R. Spillantini M. G. (2001). alpha-synuclein metabolism and aggregation is linked to ubiquitin-independent degradation by the proteasome. *FEBS Lett.* 509 22–26. 10.1016/s0014-5793(01)03115-5 11734199

[B443] Tomas-Zapico C. Diez-Zaera M. Ferrer I. Gomez-Ramos P. Moran M. A. Miras-Portugal M. T. (2012). alpha-Synuclein accumulates in huntingtin inclusions but forms independent filaments and its deficiency attenuates early phenotype in a mouse model of Huntington’s disease. *Hum. Mol. Genet.* 21 495–510. 10.1093/hmg/ddr507 22045698

[B444] Tran H. T. Chung C. H. Iba M. Zhang B. Trojanowski J. Q. Luk K. C. (2014). Alpha-synuclein immunotherapy blocks uptake and templated propagation of misfolded alpha-synuclein and neurodegeneration. *Cell Rep.* 7 2054–2065. 10.1016/j.celrep.2014.05.033 24931606 PMC4410967

[B445] Tsigelny I. F. Sharikov Y. Wrasidlo W. Gonzalez T. Desplats P. A. Crews L. (2012). Role of alpha-synuclein penetration into the membrane in the mechanisms of oligomer pore formation. *FEBS J.* 279 1000–1013. 10.1111/j.1742-4658.2012.08489.x 22251432 PMC3925782

[B446] Tufekci K. U. Meuwissen R. Genc S. Genc K. (2012). Inflammation in Parkinson’s disease. *Adv. Protein Chem. Struct. Biol.* 88 69–132. 10.1016/B978-0-12-398314-5.00004-0 22814707

[B447] Ubhi K. Rockenstein E. Mante M. Inglis C. Adame A. Patrick C. (2010). Neurodegeneration in a transgenic mouse model of multiple system atrophy is associated with altered expression of oligodendroglial-derived neurotrophic factors. *J. Neurosci.* 30 6236–6246. 10.1523/JNEUROSCI.0567-10.2010 20445049 PMC2896284

[B448] Ubhi K. Rockenstein E. Mante M. Patrick C. Adame A. Thukral M. (2008). Rifampicin reduces alpha-synuclein in a transgenic mouse model of multiple system atrophy. *Neuroreport* 19 1271–1276. 10.1097/WNR.0b013e32830b3661 18695506 PMC2885900

[B449] Ueda K. Fukushima H. Masliah E. Xia Y. Iwai A. Yoshimoto M. (1993). Molecular cloning of cDNA encoding an unrecognized component of amyloid in Alzheimer disease. *Proc. Natl. Acad. Sci. U.S.A.* 90 11282–11286. 10.1073/pnas.90.23.11282 8248242 PMC47966

[B450] Uehara T. Choong C. J. Nakamori M. Hayakawa H. Nishiyama K. Kasahara Y. (2019). Amido-bridged nucleic acid (AmNA)-modified antisense oligonucleotides targeting alpha-synuclein as a novel therapy for Parkinson’s disease. *Sci. Rep.* 9:7567. 10.1038/s41598-019-43772-9 31110191 PMC6527855

[B451] Uemura N. Yagi H. Uemura M. T. Hatanaka Y. Yamakado H. Takahashi R. (2018). Inoculation of alpha-synuclein preformed fibrils into the mouse gastrointestinal tract induces Lewy body-like aggregates in the brainstem via the vagus nerve. *Mol. Neurodegener.* 13:21. 10.1186/s13024-018-0257-5 29751824 PMC5948849

[B452] Ulmer T. S. Bax A. Cole N. B. Nussbaum R. L. (2005). Structure and dynamics of micelle-bound human alpha-synuclein. *J. Biol. Chem.* 280 9595–9603. 10.1074/jbc.M411805200 15615727

[B453] Ulusoy A. Febbraro F. Jensen P. H. Kirik D. Romero-Ramos M. (2010). Co-expression of C-terminal truncated alpha-synuclein enhances full-length alpha-synuclein-induced pathology. *Eur J. Neurosci.* 32 409–422. 10.1111/j.1460-9568.2010.07284.x 20704592

[B454] Ulusoy A. Musgrove R. E. Rusconi R. Klinkenberg M. Helwig M. Schneider A. (2015). Neuron-to-neuron alpha-synuclein propagation *in vivo* is independent of neuronal injury. *Acta Neuropathol. Commun.* 3:13. 10.1186/s40478-015-0198-y 25853980 PMC4369863

[B455] Unger M. M. Spiegel J. Dillmann K. U. Grundmann D. Philippeit H. Burmann J. (2016). Short chain fatty acids and gut microbiota differ between patients with Parkinson’s disease and age-matched controls. *Parkinsonism Relat. Disord.* 32 66–72. 10.1016/j.parkreldis.2016.08.019 27591074

[B456] Ur Rasheed M. S. Tripathi M. K. Mishra A. K. Shukla S. Singh M. P. (2016). Resveratrol protects from toxin-induced parkinsonism: plethora of proofs hitherto petty translational value. *Mol. Neurobiol.* 53 2751–2760. 10.1007/s12035-015-9124-3 25691456

[B457] Uversky V. N. Li J. Fink A. L. (2001). Evidence for a partially folded intermediate in alpha-synuclein fibril formation. *J. Biol. Chem.* 276 10737–10744. 10.1074/jbc.M010907200 11152691

[B458] Valente T. Dentesano G. Ezquerra M. Fernandez-Santiago R. Martinez-Martin J. Gallastegui E. (2020). CCAAT/enhancer binding protein delta is a transcriptional repressor of alpha-synuclein. *Cell Death Differ.* 27 509–524. 10.1038/s41418-019-0368-8 31209363 PMC7206087

[B459] Valera E. Spencer B. Fields J. A. Trinh I. Adame A. Mante M. (2017). Combination of alpha-synuclein immunotherapy with anti-inflammatory treatment in a transgenic mouse model of multiple system atrophy. *Acta Neuropathol. Commun.* 5:2. 10.1186/s40478-016-0409-1 28057080 PMC5217191

[B460] Van Den Berge N. Ferreira N. Gram H. Mikkelsen T. W. Alstrup A. K. O. Casadei N. (2019). Evidence for bidirectional and trans-synaptic parasympathetic and sympathetic propagation of alpha-synuclein in rats. *Acta Neuropathol.* 138 535–550. 10.1007/s00401-019-02040-w 31254094 PMC6778265

[B461] Van der Perren A. Gelders G. Fenyi A. Bousset L. Brito F. Peelaerts W. (2020). The structural differences between patient-derived alpha-synuclein strains dictate characteristics of Parkinson’s disease, multiple system atrophy and dementia with Lewy bodies. *Acta Neuropathol.* 139 977–1000. 10.1007/s00401-020-02157-3 32356200 PMC7244622

[B462] Vawter M. P. Dillon-Carter O. Tourtellotte W. W. Carvey P. Freed W. J. (1996). TGFbeta1 and TGFbeta2 concentrations are elevated in Parkinson’s disease in ventricular cerebrospinal fluid. *Exp. Neurol.* 142 313–322. 10.1006/exnr.1996.0200 8934562

[B463] Vekrellis K. Stefanis L. (2012). Targeting intracellular and extracellular alpha-synuclein as a therapeutic strategy in Parkinson’s disease and other synucleinopathies. *Expert Opin. Ther. Targets* 16 421–432. 10.1517/14728222.2012.674111 22480256

[B464] Vekrellis K. Xilouri M. Emmanouilidou E. Rideout H. J. Stefanis L. (2011). Pathological roles of alpha-synuclein in neurological disorders. *Parkinsonism Relat. Disord.* 10 1015–1025. 10.1016/S1474-4422(11)70213-7 22014436

[B465] Vekrellis K. Xilouri M. Emmanouilidou E. Stefanis L. (2009). Inducible over-expression of wild type alpha-synuclein in human neuronal cells leads to caspase-dependent non-apoptotic death. *J. Neurochem.* 109 1348–1362. 10.1111/j.1471-4159.2009.06054.x 19476547

[B466] Vieira B. D. Radford R. A. Chung R. S. Guillemin G. J. Pountney D. L. (2015). Neuroinflammation in multiple system atrophy: response to and cause of alpha-synuclein aggregation. *Front. Cell. Neurosci.* 9:437. 10.3389/fncel.2015.00437 26778958 PMC4700780

[B467] Vilarino-Guell C. Soto-Ortolaza A. I. Rajput A. Mash D. C. Papapetropoulos S. Pahwa R. (2011). MAPT H1 haplotype is a risk factor for essential tremor and multiple system atrophy. *Neurology* 76 670–672. 10.1212/WNL.0b013e31820c30c1 21321341 PMC3053340

[B468] Vinueza-Gavilanes R. Inigo-Marco I. Larrea L. Lasa M. Carte B. Santamaria E. (2020). N-terminal acetylation mutants affect alpha-synuclein stability, protein levels and neuronal toxicity. *Neurobiol. Dis.* 137:104781. 10.1016/j.nbd.2020.104781 31991248

[B469] Vogiatzi T. Xilouri M. Vekrellis K. Stefanis L. (2008). Wild type alpha-synuclein is degraded by chaperone-mediated autophagy and macroautophagy in neuronal cells. *J. Biol. Chem.* 283 23542–23556. 10.1074/jbc.M801992200 18566453 PMC2527094

[B470] Volles M. J. Lansbury P. T. Jr. (2002). Vesicle permeabilization by protofibrillar alpha-synuclein is sensitive to Parkinson’s disease-linked mutations and occurs by a pore-like mechanism. *Biochemistry* 41 4595–4602. 10.1021/bi0121353 11926821

[B471] Volpicelli-Daley L. A. Luk K. C. Lee V. M. (2014). Addition of exogenous alpha-synuclein preformed fibrils to primary neuronal cultures to seed recruitment of endogenous alpha-synuclein to Lewy body and Lewy neurite-like aggregates. *Nat. Protoc.* 9 2135–2146. 10.1038/nprot.2014.143 25122523 PMC4372899

[B472] Volpicelli-Daley L. A. Luk K. C. Patel T. P. Tanik S. A. Riddle D. M. Stieber A. (2011). Exogenous alpha-synuclein fibrils induce Lewy body pathology leading to synaptic dysfunction and neuron death. *Neuron* 72 57–71. 10.1016/j.neuron.2011.08.033 21982369 PMC3204802

[B473] Wagner J. Ryazanov S. Leonov A. Levin J. Shi S. Schmidt F. (2013). Anle138b: a novel oligomer modulator for disease-modifying therapy of neurodegenerative diseases such as prion and Parkinson’s disease. *Acta Neuropathol.* 125 795–813. 10.1007/s00401-013-1114-9 23604588 PMC3661926

[B474] Wakabayashi K. Hayashi S. Kakita A. Yamada M. Toyoshima Y. Yoshimoto M. (1998a). Accumulation of alpha-synuclein/NACP is a cytopathological feature common to Lewy body disease and multiple system atrophy. *Acta Neuropathol.* 96 445–452. 10.1007/s004010050918 9829807

[B475] Wakabayashi K. Yoshimoto M. Tsuji S. Takahashi H. (1998b). Alpha-synuclein immunoreactivity in glial cytoplasmic inclusions in multiple system atrophy. *Neurosci. Lett.* 249 180–182. 10.1016/s0304-3940(98)00407-8 9682846

[B476] Wang B. Underwood R. Kamath A. Britain C. McFerrin M. B. McLean P. J. (2018). 14-3-3 proteins reduce cell-to-cell transfer and propagation of pathogenic alpha-synuclein. *J. Neurosci.* 38 8211–8232. 10.1523/JNEUROSCI.1134-18.2018 30093536 PMC6146494

[B477] Wang T. Hay J. C. (2015). Alpha-synuclein toxicity in the early secretory pathway: how it drives neurodegeneration in Parkinsons disease. *Front. Neurosci.* 9:433. 10.3389/fnins.2015.00433 26617485 PMC4641903

[B478] Wang W. Nguyen L. T. Burlak C. Chegini F. Guo F. Chataway T. (2016). Caspase-1 causes truncation and aggregation of the Parkinson’s disease-associated protein alpha-synuclein. *Proc. Natl. Acad. Sci. U.S.A.* 113 9587–9592. 10.1073/pnas.1610099113 27482083 PMC5003239

[B479] Wang W. Perovic I. Chittuluru J. Kaganovich A. Nguyen L. T. Liao J. (2011). A soluble alpha-synuclein construct forms a dynamic tetramer. *Proc. Natl. Acad. Sci. U.S.A.* 108 17797–17802. 10.1073/pnas.1113260108 22006323 PMC3203798

[B480] Wang X. Becker K. Levine N. Zhang M. Lieberman A. P. Moore D. J. (2019). Pathogenic alpha-synuclein aggregates preferentially bind to mitochondria and affect cellular respiration. *Acta Neuropathol. Commun.* 7:41. 10.1186/s40478-019-0696-4 30871620 PMC6419482

[B481] Watson M. B. Richter F. Lee S. K. Gabby L. Wu J. Masliah E. (2012). Regionally-specific microglial activation in young mice over-expressing human wildtype alpha-synuclein. *Exp. Neurol.* 237 318–334. 10.1016/j.expneurol.2012.06.025 22750327 PMC3443323

[B482] Watts J. C. Giles K. Oehler A. Middleton L. Dexter D. T. Gentleman S. M. (2013). Transmission of multiple system atrophy prions to transgenic mice. *Proc. Natl. Acad. Sci. U.S.A.* 110 19555–19560. 10.1073/pnas.1318268110 24218576 PMC3845125

[B483] Waxman E. A. Mazzulli J. R. Giasson B. I. (2009). Characterization of hydrophobic residue requirements for alpha-synuclein fibrillization. *Biochemistry* 48 9427–9436. 10.1021/bi900539p 19722699 PMC2758333

[B484] Webb J. L. Ravikumar B. Atkins J. Skepper J. N. Rubinsztein D. C. (2003). Alpha-Synuclein is degraded by both autophagy and the proteasome. *J. Biol. Chem.* 278 25009–25013. 10.1074/jbc.M300227200 12719433

[B485] Weihofen A. Liu Y. Arndt J. W. Huy C. Quan C. Smith B. A. (2019). Development of an aggregate-selective, human-derived alpha-synuclein antibody BIIB054 that ameliorates disease phenotypes in Parkinson’s disease models. *Neurobiol. Dis.* 124 276–288. 10.1016/j.nbd.2018.10.016 30381260

[B486] Weinreb P. H. Zhen W. Poon A. W. Conway K. A. Lansbury P. T. Jr. (1996). NACP, a protein implicated in Alzheimer’s disease and learning, is natively unfolded. *Biochemistry* 35 13709–13715. 10.1021/bi961799n 8901511

[B487] Wersinger C. Jeannotte A. Sidhu A. (2006a). Attenuation of the norepinephrine transporter activity and trafficking via interactions with alpha-synuclein. *Eur J. Neurosci.* 24 3141–3152. 10.1111/j.1460-9568.2006.05181.x 17156375

[B488] Wersinger C. Rusnak M. Sidhu A. (2006b). Modulation of the trafficking of the human serotonin transporter by human alpha-synuclein. *Eur. J. Neurosci.* 24 55–64. 10.1111/j.1460-9568.2006.04900.x 16882008

[B489] Whittaker H. T. Qui Y. Bettencourt C. Houlden H. (2017). Multiple system atrophy: genetic risks and alpha-synuclein mutations. *F1000Res.* 6:2072. 10.12688/f1000research.12193.1 29225795 PMC5710304

[B490] Winner B. Jappelli R. Maji S. K. Desplats P. A. Boyer L. Aigner S. (2011). *In vivo* demonstration that alpha-synuclein oligomers are toxic. *Proc. Natl. Acad. Sci. U.S.A.* 108 4194–4199. 10.1073/pnas.1100976108 21325059 PMC3053976

[B491] Winslow A. R. Chen C. W. Corrochano S. Acevedo-Arozena A. Gordon D. E. Peden A. A. (2010). alpha-Synuclein impairs macroautophagy: implications for Parkinson’s disease. *J. Cell Biol.* 190 1023–1037. 10.1083/jcb.201003122 20855506 PMC3101586

[B492] Witt S. N. (2013). Molecular chaperones, alpha-synuclein, and neurodegeneration. *Mol. Neurobiol.* 47 552–560. 10.1007/s12035-012-8325-2 22923346 PMC3537861

[B493] Woerman A. L. Stohr J. Aoyagi A. Rampersaud R. Krejciova Z. Watts J. C. (2015). Propagation of prions causing synucleinopathies in cultured cells. *Proc. Natl. Acad. Sci. U.S.A.* 112 E4949–E4958. 10.1073/pnas.1513426112 26286986 PMC4568231

[B494] Wrasidlo W. Tsigelny I. F. Price D. L. Dutta G. Rockenstein E. Schwarz T. C. (2016). A de novo compound targeting alpha-synuclein improves deficits in models of Parkinson’s disease. *Brain* 139(Pt 12), 3217–3236. 10.1093/brain/aww238 27679481 PMC5840882

[B495] Wu B. Liu Q. Duan C. Li Y. Yu S. Chan P. (2011). Phosphorylation of alpha-synuclein upregulates tyrosine hydroxylase activity in MN9D cells. *Acta Histochem.* 113 32–35. 10.1016/j.acthis.2009.07.007 19683335

[B496] Wu F. Xu H. D. Guan J. J. Hou Y. S. Gu J. H. Zhen X. C. (2015). Rotenone impairs autophagic flux and lysosomal functions in Parkinson’s disease. *Neuroscience* 284 900–911. 10.1016/j.neuroscience.2014.11.004 25446361

[B497] Wu Q. Takano H. Riddle D. M. Trojanowski J. Q. Coulter D. A. Lee V. M. (2019). alpha-Synuclein (alphaSyn) preformed fibrils induce endogenous alphaSyn aggregation, compromise synaptic activity and enhance synapse loss in cultured excitatory hippocampal neurons. *J. Neurosci.* 39 5080–5094. 10.1523/JNEUROSCI.0060-19.2019 31036761 PMC6595958

[B498] Wu Y. Li X. Zhu J. X. Xie W. Le W. Fan Z. (2011). Resveratrol-activated AMPK/SIRT1/autophagy in cellular models of Parkinson’s disease. *Neurosignals* 19 163–174. 10.1159/000328516 21778691 PMC3699815

[B499] Xilouri M. Brekk O. R. Landeck N. Pitychoutis P. M. Papasilekas T. Papadopoulou-Daifoti Z. (2013a). Boosting chaperone-mediated autophagy *in vivo* mitigates alpha-synuclein-induced neurodegeneration. *Brain* 136(Pt 7), 2130–2146. 10.1093/brain/awt131 23757764

[B500] Xilouri M. Brekk O. R. Polissidis A. Chrysanthou-Piterou M. Kloukina I. Stefanis L. (2016a). Impairment of chaperone-mediated autophagy induces dopaminergic neurodegeneration in rats. *Autophagy* 12 2230–2247. 10.1080/15548627.2016.1214777 27541985 PMC5103347

[B501] Xilouri M. Brekk O. R. Stefanis L. (2013b). alpha-Synuclein and protein degradation systems: a reciprocal relationship. *Mol. Neurobiol.* 47 537–551. 10.1007/s12035-012-8341-2 22941029

[B502] Xilouri M. Brekk O. R. Stefanis L. (2016b). Autophagy and alpha-synuclein: relevance to Parkinson’s disease and related synucleopathies. *Mov. Disord.* 31 178–192. 10.1002/mds.26477 26813776

[B503] Xilouri M. Vogiatzi T. Vekrellis K. Park D. Stefanis L. (2009). Abberant alpha-synuclein confers toxicity to neurons in part through inhibition of chaperone-mediated autophagy. *PLoS One* 4:e5515. 10.1371/journal.pone.0005515 19436756 PMC2677735

[B504] Xu J. Wu X. S. Sheng J. Zhang Z. Yue H. Y. Sun L. (2016). alpha-synuclein mutation inhibits endocytosis at mammalian central nerve terminals. *J. Neurosci.* 36 4408–4414. 10.1523/JNEUROSCI.3627-15.2016 27098685 PMC4837680

[B505] Xu Y. Liu C. Chen S. Ye Y. Guo M. Ren Q. (2014). Activation of AMPK and inactivation of Akt result in suppression of mTOR-mediated S6K1 and 4E-BP1 pathways leading to neuronal cell death in *in vitro* models of Parkinson’s disease. *Cell. Signal.* 26 1680–1689. 10.1016/j.cellsig.2014.04.009 24726895 PMC4039615

[B506] Xu Z. Graham K. Foote M. Liang F. Rizkallah R. Hurt M. (2013). 14-3-3 protein targets misfolded chaperone-associated proteins to aggresomes. *J. Cell Sci.* 126(Pt 18), 4173–4186. 10.1242/jcs.126102 23843611 PMC3772389

[B507] Yan J. Q. Yuan Y. H. Gao Y. N. Huang J. Y. Ma K. L. Gao Y. (2014). Overexpression of human E46K mutant alpha-synuclein impairs macroautophagy via inactivation of JNK1-Bcl-2 pathway. *Mol. Neurobiol.* 50 685–701. 10.1007/s12035-014-8738-1 24833599

[B508] Yang S. Y. Beavan M. Chau K. Y. Taanman J. W. Schapira A. H. V. (2017). A human neural crest stem cell-derived dopaminergic neuronal model recapitulates biochemical abnormalities in GBA1 mutation carriers. *Stem Cell Rep.* 8 728–742. 10.1016/j.stemcr.2017.01.011 28216145 PMC5355624

[B509] Yang Y. Fukui K. Koike T. Zheng X. (2007). Induction of autophagy in neurite degeneration of mouse superior cervical ganglion neurons. *Eur. J. Neurosci.* 26 2979–2988. 10.1111/j.1460-9568.2007.05914.x 18001292

[B510] Yazawa I. Giasson B. I. Sasaki R. Zhang B. Joyce S. Uryu K. (2005). Mouse model of multiple system atrophy alpha-synuclein expression in oligodendrocytes causes glial and neuronal degeneration. *Neuron* 45 847–859. 10.1016/j.neuron.2005.01.032 15797547

[B511] Zarranz J. J. Alegre J. Gomez-Esteban J. C. Lezcano E. Ros R. Ampuero I. (2004). The new mutation, E46K, of alpha-synuclein causes Parkinson and Lewy body dementia. *Ann. Neurol.* 55 164–173. 10.1002/ana.10795 14755719

[B512] Zavodszky E. Seaman M. N. Moreau K. Jimenez-Sanchez M. Breusegem S. Y. Harbour M. E. (2014). Mutation in VPS35 associated with Parkinson’s disease impairs WASH complex association and inhibits autophagy. *Nat. Commun.* 5:3828. 10.1038/ncomms4828 24819384 PMC4024763

[B513] Zhang Q. Xu Y. Lee J. Jarnik M. Wu X. Bonifacino J. S. (2020). A myosin-7B-dependent endocytosis pathway mediates cellular entry of alpha-synuclein fibrils and polycation-bearing cargos. *Proc. Natl. Acad. Sci. U.S.A.* 117 10865–10875. 10.1073/pnas.1918617117 32366666 PMC7245082

[B514] Zharikov A. D. Cannon J. R. Tapias V. Bai Q. Horowitz M. P. Shah V. (2015). shRNA targeting alpha-synuclein prevents neurodegeneration in a Parkinson’s disease model. *J. Clin. Invest.* 125 2721–2735. 10.1172/JCI64502 26075822 PMC4563670

[B515] Zhu M. Rajamani S. Kaylor J. Han S. Zhou F. Fink A. L. (2004). The flavonoid baicalein inhibits fibrillation of alpha-synuclein and disaggregates existing fibrils. *J. Biol. Chem.* 279 26846–26857. 10.1074/jbc.M403129200 15096521

[B516] Zondler L. Kostka M. Garidel P. Heinzelmann U. Hengerer B. Mayer B. (2017). Proteasome impairment by alpha-synuclein. *PLoS One* 12:e0184040. 10.1371/journal.pone.0184040 28945746 PMC5612461

[B517] Zou J. Guo Y. Wei L. Yu F. Yu B. Xu A. (2020). Long noncoding RNA POU3F3 and alpha-Synuclein in Plasma L1CAM exosomes combined with beta-Glucocerebrosidase activity: potential predictors of Parkinson’s disease. *Neurotherapeutics.* 10.1007/s13311-020-00842-5 [Epub ahead of print]. 32236821 PMC7609611

